# Associated factors and global adherence of cervical cancer screening in 2019: a systematic analysis and modelling study

**DOI:** 10.1186/s12992-022-00890-w

**Published:** 2022-12-09

**Authors:** Wanting Zhang, Kai Gao, Freya J. I. Fowkes, Davies Adeloye, Igor Rudan, Peige Song, Mingjuan Jin, Kun Chen

**Affiliations:** 1 Department of Public Health, Second Affiliated Hospital, Zhejiang University School of Medicine, Hangzhou, 310058 China; 2 Burnet Institute, Melbourne, VIC Australia; 3 Centre for Global Health Research, Usher Institute of Population Health Sciences and Informatics, University of Edinburgh, Edinburgh, UK

**Keywords:** Cervical cancer, Screening, Adherence, Associated factors, Systematic review, Meta-analysis, Modelling study

## Abstract

**Background:**

Cervical cancer screening is vital for its prevention. Adherence is a crucial indicator that implies the individual willingness to take cervical cancer screening. We aimed to estimate the global and regional adherence rates of cervical cancer screening in 2019 and identify its associated factors among general women.

**Method:**

We searched studies in PubMed, Web of Science, Embase, China National Knowledge Infrastructure, Wanfang Database, ProQuest theses database and Google Web, without a lower time limit and until 23 June, 2021. Survey studies were considered eligible if they investigated cervical cancer screening adherence among general women, with data on sample size, the number of adherent subjects, and/or adherence rate. Random-effects were used to pool the odds ratios (ORs) of associated factors of adherence. Using modelling analysis, we estimated 2019 overall and age-specific adherence rates at the global and regional levels in women aged 20–69 years.

**Results:**

Eight thousand two hundred ninety records were identified, and 153 articles were included. Being married (vs not married: OR, 1.34; 95% confidence interval [CI]: 1.23–1.46), higher educational attainment (higher than high school vs less than high school: OR, 1.44; 95% CI: 1.35–1.53), having healthcare (OR, 1.64; 95% CI: 1.43–1.88), former smoking (OR, 1.20; 95% CI: 1.07–1.34), physical activity (OR, 1.19; 95% CI: 1.05–1.36), parity (OR, 1.07; 95% CI: 1.01–1.12), and chronic disease (OR, 1.17; 95% CI: 1.04–1.32) were associated with better adherence, whereas obesity (vs normal: OR, 0.85; 95% CI: 0.74–0.97) and current smoking (vs former/never: OR, 0.64; 95% CI: 0.54–0.76) were associated with worse adherence. In 2019, the adherence was at 33.66% (95% CI: 23.34–39.30%) worldwide, and was higher in high-income countries (HICs) (75.66, 95% CI: 66.74–82.81%) than in low and middle-income countries (LMICs) (24.91, 95% CI: 14.30–30.24%). It varied across regions, the highest in the European region (65.36, 95% CI: 55.40–74.19%), but the lowest in the African region (5.28, 95% CI: 3.43–8.03%).

**Conclusions:**

Cervical cancer screening adherence remained low globally, exhibiting geographical discrepancy with HICs higher than LMICs. Further implementations of screening programs should comprehensively consider the local economy, social benefits, and demographic structure to adapt delivery for vulnerable or underserved women to boost screening adherence.

**Supplementary Information:**

The online version contains supplementary material available at 10.1186/s12992-022-00890-w.

## Background

Cervical cancer is the fourth most common cancer among women worldwide, with approximately 604,000 new cases diagnosed and 342,000 deaths reported in 2020 [1]. Globally, geographical disparities in cervical cancer burden are conspicuous, with low and middle-income countries (LMICs) suffering higher incidences and mortalities compared with high-income countries (HICs) [2]. These disparities largely result from the higher prevalence of cervical cancer risk factors in LMICs, such as high-risk human papillomavirus (HPV) infection, human immunodeficiency virus (HIV) infection, smoking, and a lack of medical infrastructure and healthcare resources [3–5].

In May 2018, the World Health Organization (WHO) issued an ambitious call for all countries to make a commitment to ending the suffering from cervical cancer, by which the proposed key prevention strategy includes HPV vaccination for girls aged 9–14 years and screening for women from 30 years of age [6, 7]. The latter could reduce both incidence and mortality by removal of precancerous lesions and treatment of early-stage cancer, is still needed to be strengthened worldwide, even in countries where HPV vaccine has already been introduced [2, 3, 8]. Since women are constantly at risk of cervical carcinogenesis cross lifespan, receiving regular screening is crucial to ensure screening effectiveness. Adherence rate, the proportion of participants who are adherent to guidelines for regular screening, is a key indicator reflecting the actual willingness to take cervical cancer screening at individual level. It is influenced by multiple factors, among which social determinants of health (SDH) might have important impacts as they are typically non-medical factors including healthcare access and quality, education access and quality, social and community context, economic stability, and neighborhood and built environment [9]. A comprehensive understanding of screening adherence is essential for policymakers and stakeholders to develop effective management and intervention policies, and to optimize health resource allocations. To the best of our knowledge, there is no estimation of screening adherence available at the global level, inadequate evaluation of regional variations, and a dearth of studies exploring the factors that influence screening adherence.

To fill this knowledge gap and to promote actions across world regions, this study aimed to quantify the associations of the main influencing factors with cervical cancer screening adherence, to estimate the overall and age-specific adherence rates at the global and regional levels in 2019.

## Methods

### Protocol and information sources

The systematic analysis and modelling study complied with the Preferred Reporting Items for Systematic Reviews and Meta-analyses (PRISMA) reporting guideline [10], and the Guidelines for Accurate and Transparent Health Estimates Reporting (GATHER) [11]. The study protocol was prospectively registered on PROSPERO (number CRD42020215140).

### Search strategy and selection criteria

We searched five databases (PubMed, Web of Science, Embase, China National Knowledge Infrastructure, and Wanfang Database) without a lower time limit and until 23 June, 2021, to identify observational studies that investigated cervical cancer screening adherence among general women. A combination of search terms relating to cervical cancer screening and adherence was adopted, without language restriction. Additionally, we searched ProQuest theses database and Google Web for potentially eligible grey literature. Reference lists of related systematic reviews and the included articles were screened to identify additional relevant studies. Full details of the search strategies are listed in the Appendix (Table S1).

Studies were retained if they reported data on adherence to cervical cancer screening, including sample size, the number of adherent subjects, and/or adherence rate. Studies that were conducted in hospitals or in special population (e.g. HIV-infected women) were excluded. Reviews, conference abstracts, commentaries, and case series were also excluded.

Against the selection criteria, two authors (WZ and KG) independently reviewed all titles, abstracts, and full-text articles that were potentially relevant. Inconsistencies during the review process were resolved through discussion with a third author if necessary (MJ).

### Data extraction

The following data were independently extracted from the included articles by two authors (WZ and KG): author, year published, year investigated, country, region (WHO, World Bank [WB] region), place (rural, urban, both), Socio-demographic Index (SDI), data source (self-reporting, objective-recording), screening guideline information (guideline name, definition of adherence, screening methods and intervals), sample size, the number of participants who were adherent to cervical cancer screening guideline, age and education of participants. As cervical cancer screening strategies and programs were generally adapted and implemented by the local health care system, in full considerations of country- and area-specific contexts (e.g., differences in cervical cancer prevalence, economical level, social and cultural factors), it was anticipated that the recommended methods and intervals would not be completely consistent across studies. Therefore, we defined the adherence rate of cervical cancer screening as the proportion of participants who were adherent to local guidelines. The geographical regions of study were designated as African Region (AFR), Region of the Americas (AMR), Southeast Asia Region (SEAR), European Region (EUR), Eastern Mediterranean Region (EMR), and Western Pacific Region (WPR) using the WHO categorization, and the regions were also categorized into HICs and LMICs according to the WB criteria. Furthermore, SDI was also obtained from Global Burden of Disease Study 2019, which is a composite indicator of total fertility rate under the age of 25 years, mean education for those aged 15 years and older, and lag distributed income per capita, reflecting the overall socioeconomic-development of a country [12]. The data sources were defined as self-reporting (by survey to subjects) and objective-recording (by checking the records in health care systems and hospitals, etc.). Wherever applicable, data on age-specific or investigation year-specific adherence rates were separately extracted. For studies in which the year investigated was not provided, we imputed the investigation year by subtracting five years from the year published (Table S2). For studies where reported censored age ranges, we imputed the missing age band by taking the same width reported in other age ranges on the same study.

For studies, where associated factors of adherence to cervical cancer screening were explored using multivariable logistic regressions, the definitions of each factor and the corresponding effect size estimates (odds ratio [OR] and 95% confidence interval [CI]) were extracted.

### Quality assessment

We evaluated the risk of bias of included studies using a quality scale based on the Strengthening the Reporting of Observational Studies in Epidemiology statement (STROBE) [13]. This scale assesses sources of bias from five dimensions: sample population, sample size, participation, outcome assessment, and analytical methods (Table S3). The total score ranges from 0 to 10, and studies with a score greater than 6 points were considered high quality. Any discrepancies were resolved by consensus through group discussion.

### Statistical analysis

For meta-analysis, the effects of associated factors on adherence to cervical cancer screening were synthesized by random-effects (DerSimonian) model [14]. Only factors that shared similar definitions and had at least three individual multivariate-adjusted effect sizes from different studies were included [15].

For modelling analysis, one study might provide two or more data points about cervical cancer screening adherence in different age groups or investigation years. To make full use of this information, we used a multilevel mixed-effects meta-regression approach. To take the country-specific context of screening guidelines and policies into account, the country identification number (*μ*_*i*_) was employed as the random effect in models. Given that adherence rate (*p*) = number of participants adherent to guideline / number of participants, we stabilized the rate with the logit link as follows:

$$\textrm{logit}(p)=\ln \left(\frac{p}{1-p}\right)=\ln (odds)=\alpha +{\beta}_1{x}_1+{\beta}_2{x}_2+\cdots +{\mu}_i$$

Where *α* is the intercept term, *β* is the coefficient, and *x* is the variable.

The effects of cluster-level variables, including age, place, year investigated, SDI (per 1%), and WHO region, were assessed by univariable and multivariable meta-regression (Table S4). Age, SDI, and year investigated were revealed to be significantly associated with cervical screening adherence based on those analyses. We only included age and SDI in our final model since there was considerable collinearity between SDI and year investigated, and SDI may have a broader interpretation on social health status than chronological years. Therefore,

$$\textrm{logit}(p)=\alpha +{\beta}_1\ast \textrm{age}+{\beta}_2\ast \textrm{SDI}+{\mu}_i$$

then,

$$p=\frac{\exp \left(\alpha +{\beta}_1\ast \textrm{age}+{\beta}_2\ast \textrm{SDI}+{\mu}_i\right)}{1+\exp \left(\alpha +{\beta}_1\ast \textrm{age}+{\beta}_2\ast \textrm{SDI}+{\mu}_i\right)}$$

We only estimated the adherence rates for women aged 20–69 years, considering this is a proper age range for cervical cancer screening. To address both the features of geography and income, we classified the world into ten regions, namely AMR (HIC), EUR (HIC), EMR (HIC), WPR (HIC), AFR (LMIC), AMR (LMIC), SEAR (LMIC), EUR (LMIC), EMR (LMIC) and WPR (LMIC). The overall and age-specific adherence rates for these ten regions were generated for the year 2019 based on the above model. The SDI value for each region was calculated as the weighted-average of country-specific SDI in this region, and the weight was population size in 2019.

For meta-analysis of national adherence rate, we pooled the rates using random-effects model for countries with adequate adherence data.

A two-sided *P* value of less than 0.05 was considered statistically significant. All analyses were performed with R version 4.0.2 (R Foundation for Statistical Computing) and Stata version 14.0 (College Station, TX, USA).

## Results

**Fig. 1:**
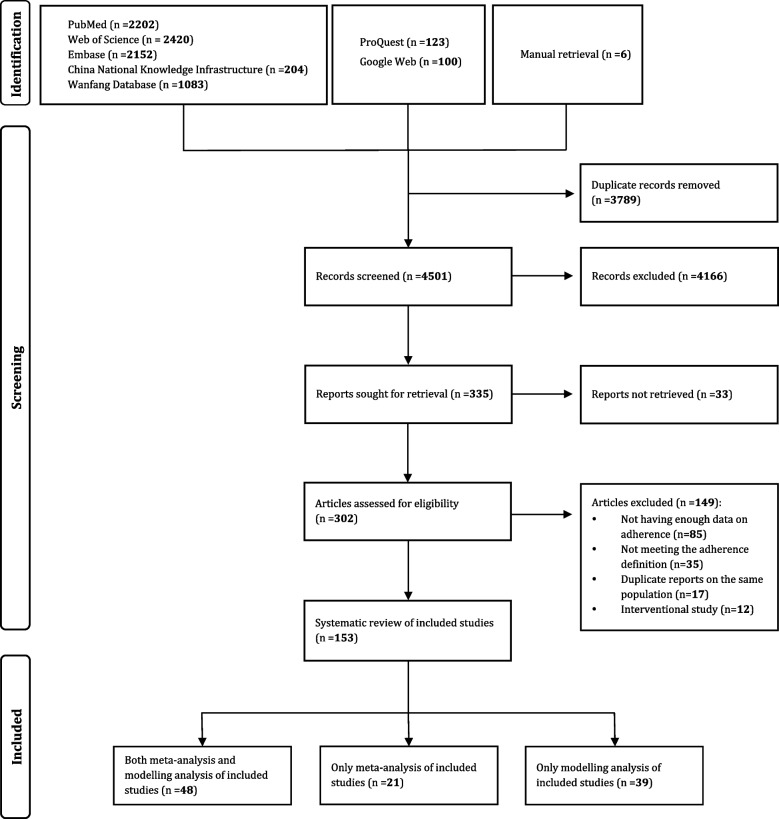
PRISMA flow diagram of study selection

A total of 8284 records were identified in the initial literature research. After removing duplicates and irrelevant records that lacked information on cervical cancer screening adherence, and adding 6 articles manually, 302 potentially relevant articles were evaluated in full-text, further 149 were excluded due to lack of enough data, having imprecise definition, duplicate reports based on the same population and interventional studies (Fig. 1). Finally, 153 studies were considered eligible and included into the current systematic review, among which 69 reported potential associated factors, and 87 provided age-specific adherence rates of cervical cancer screening.

The characteristics of the included studies were shown in the Appendix (Table S5 and Table S6). Among the included studies, 87 were investigated in AMR, 39 in EUR, 25 in WPR, 1 in SEAR, and 1 in AFR (Fig. S1). The data sources of these studies were self-reporting in 133 studies and objective-recording in 20 studies. As for the screening characteristics, the methods used were Pap test in 138 studies, alternative choices (Pap test, or combinative use of Pap test and HPV test) in 5 studies, co-testing (Pap test and HPV test) in 1 study, and unspecified in 9 studies; and the intervals used were less than 3 years in 32 studies, 3 years in 96 studies, longer than 3 years in 9 studies, and differential interval (e.g., only cytology every 3 years, and co-testing with an HPV test and cytology every 5 years) in 16 studies. The sample size of these studies were 60 studies recruited less than 1000 women, 40 studies recruited 1000 to 4999 women, and 53 studies recruited 5000 and more women. Quality scores of each eligible study were demonstrated in the Appendix (Table S7).

### Associated factors of cervical cancer screening adherence

Eleven potentially associated factors for cervical cancer screening adherence, including demographic characteristics (marital status), social determinants of health (education level, healthcare, and employment status), lifestyle factors (body-mass index [BMI], smoking, alcohol drinking, and physical activity), and personal history (parity, mental illness, and chronic disease), were summarized in the Table 1. Better adherence was positively associated with being married (vs not married: OR, 1.34; 95% CI: 1.23–1.46; vs single: OR, 1.62; 95% CI, 1.36–1.93) and being partnered (vs not partnered: OR, 1.41; 95% CI:1.13–1.75) in demographic characteristics, higher educational attainment (high school vs less than high school: OR, 1.31; 95% CI: 1.24–1.38; higher than high school vs less than high school: OR, 1.44; 95% CI: 1.35–1.53) and having healthcare (insurance: OR, 1.64; 95% CI: 1.43–1.88; healthcare coverage: OR, 2.07; 95% CI: 1.59–2.69) in social determinants of health, former smoking (vs never: OR, 1.20; 95% CI: 1.07–1.34) and physical activity (OR, 1.19; 95% CI: 1.05–1.36) in lifestyle factors, and having parity (OR, 1.07; 95% CI: 1.01–1.12) and chronic disease (OR, 1.17; 95% CI: 1.04–1.32) in personal history. However, worse adherence was associated with obesity (vs normal/BMI 18.5–24.9 kg/m^2^: OR, 0.85; 95% CI: 0.74–0.97) and current smoking (vs former/never smoking: OR, 0.64; 95% CI: 0.54–0.76) in lifestyle factors. Details of meta-analyses for each associated factor are provided in the Appendix (Table S8).

**Table 1:** Synthesized effect size of associated factors for adherence to cervical cancer screening

	No. of studies	Odds ratio (95%CI)	Heterogeneity	
			***I***^***2***^, %	Q test***-P*** value
**Demographic characteristics**				
Associated factor 1: Marital status				
Married vs Not married	15	1.34 (1.23–1.46)	98.3	< 0.001
Married vs Single	12	1.62 (1.36–1.93)	92.8	< 0.001
Partnered vs Not partnered	9	1.41 (1.13–1.75)	78.6	< 0.001
Currently married vs Never married	3	3.76 (2.61–5.44)	0.0	0.548
Previously married vs Never married	3	2.63 (1.66–4.15)	0.0	0.403
Married vs Divorced/Widowed/Separated	5	1.14 (1.02–1.28)	98.7	< 0.001
Divorced/Widowed/Separated vs Single	5	1.51 (1.22–1.87)	91.5	< 0.001
**Social determinants of health**				
Associated factor 2: Education level				
High school vs less than high school	53	1.31 (1.24–1.38)	93.8	< 0.001
Higher than high school vs less than high school	47	1.44 (1.35–1.53)	95.1	< 0.001
Associated factor 3a: Healthcare-Insurance	8	1.64 (1.43–1.88)	3.9	0.400
Associated factor 3b: Healthcare-Healthcare coverage	4	2.07 (1.59–2.69)	83.5	< 0.001
Associated factor 4: Employment status	20	1.05 (0.97–1.13)	95.3	< 0.001
**Lifestyle factors**				
Associated factor 5: Body-mass index				
Obesity: BMI ≥30.0 kg/m^2^ vs Non-obesity	9	0.78 (0.72–0.83)	0.0	0.920
Underweight: BMI < 18.5 kg/m^2^ vs Normal: BMI 18.5–24.9 kg/m^2^	8	0.76 (0.58–1.00)	73.9	< 0.001
Overweight: BMI 25.0–30.0 kg/m^2^ vs Normal: BMI 18.5–24.9 kg/m^2^	12	0.98 (0.90–1.06)	52.6	0.017
Obesity: BMI ≥30.0 kg/m^2^ vs Normal: BMI 18.5–24.9 kg/m^2^	12	0.85 (0.74–0.97)	84.1	< 0.001
Associated factor 6: Smoking				
Smoker vs Non-smoker	14	0.87 (0.75–1.01)	84.6	< 0.001
Current vs Former/Never	5	0.64 (0.54–0.76)	32.8	0.203
Current vs Never	11	0.89 (0.78–1.02)	90.4	< 0.001
Former vs Never	7	1.20 (1.07–1.34)	84.9	< 0.001
Associated factor 7: Alcohol drinking	12	1.02 (0.86–1.21)	80.5	< 0.001
Associated factor 8: Physical activity	11	1.19 (1.05–1.36)	88.8	< 0.001
**Personal history**				
Associated factor 9: Parity	11	1.07 (1.01–1.12)	92.5	< 0.001
Associated factor 10: Mental illness	3	1.07 (0.92–1.25)	90.0	< 0.001
Associated factor 11: Chronic disease	9	1.17 (1.04–1.32)	80.2	< 0.001

*Abbreviations*: *BMI* body-mass index, *CI* confidence interval. The definitions of some associated factors varied slightly across studies. Odds ratios for binary variable associated factors indicated better adherence to cervical cancer screening compared with those without the associated factor, except for married (vs not married), married (vs single), partnered (vs not partnered), currently married (vs never married), previously married (vs never married), married (vs divorced/widowed/separated), divorced/widowed/separated (vs single), high school (vs less than high school), higher than high school (vs less than high school), underweight/BMI < 18.5 kg/m^2^ (vs normal/BMI 18.5–24.9 kg/m^2^), overweight/BMI 25.0–30.0 kg/m^2^ (vs normal/BMI 18.5–24.9 kg/m^2^), obesity/BMI ≥ 30.0 kg/m^2^ (vs normal/BMI 18.5–24.9 kg/m^2^), and smoker (vs non-smoker), current smoker (vs former/never), current smoker (vs never), former smoker (vs never)

### Global and regional cervical cancer screening adherence

**Fig. 2:**
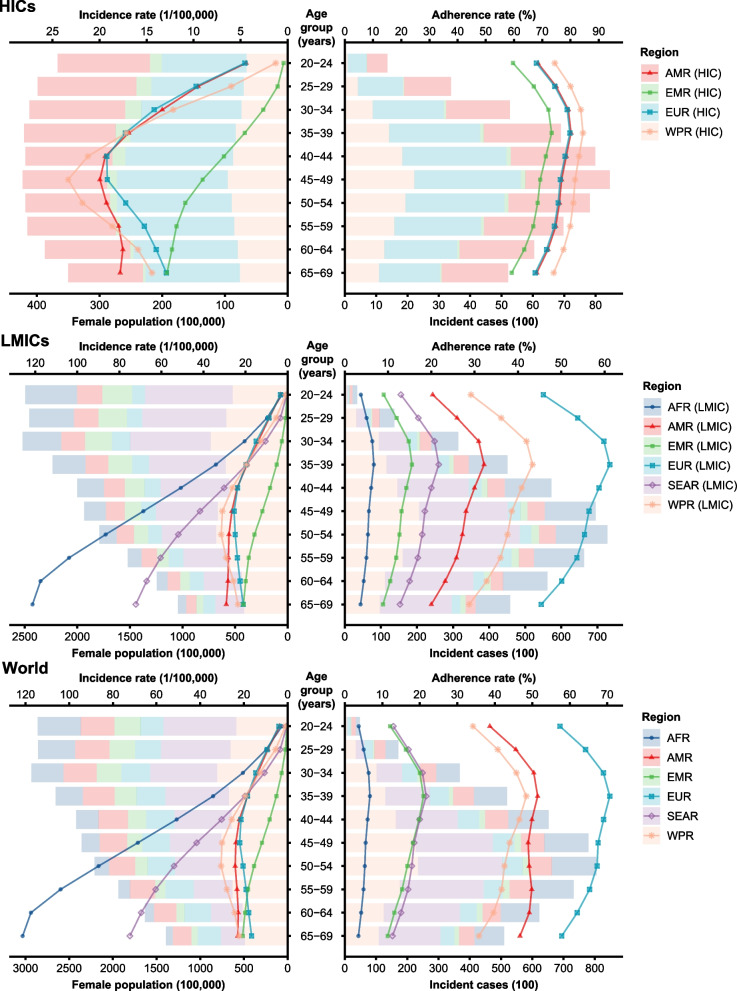
The estimated adherence rates of cervical cancer screening in 2019 across regions. Abbreviations: AFR: African Region; AMR: Region of the Americas; EMR: Eastern Mediterranean Region; EUR: European Region; HICs: high-income countries; LMICs: low and middle-income countries; SEAR: South-East Asia Region; WPR: Western Pacific Region. The female population in 2019 was adopted from the United Nations Population Division, and the age-specific cervical cancer incidence was adopted from the Global Cancer Observatory

Based on 327 data points extracted from the included articles, meta-regression analysis showed an inverted sluggish U-shaped dose-response relationship of the adherence rates with age increase (an evident increase from 20 to 35 years old, a very weak decrease from 35 to 55 years old, and a relatively obvious decrease after 55 years old) in all three SDI groups (Fig. S2). Moreover, adherence rates were always higher in more developed regions with a higher SDI. After applying age structure and SDI distribution in 2019, the adherence rate was 33.66% (95% CI, 23.34–39.30%) worldwide in women aged 20–69 years, being 2-fold higher in HICs than in LMICs (75.66, 95% CI, 66.74–82.81% vs 24.91, 95% CI, 14.30–30.24%) (Table 2 and Fig. 2). Generally, the region- and age-specific adherence rates were relatively high in women aged 30–39, 49–49, and 50–59 years, but relatively low in those aged 20–29 and 60–69 years. The adherence rates of cervical cancer screening varied extensively across regions, being the highest in EUR (65.36, 95% CI, 55.40–74.19%), but the lowest in AFR (5.28, 95% CI, 3.43–8.03%), which partly explained why the incidence of cervical cancer in EUR peaked at the age of 40–49 years and then gradually decreased, but kept increasing with age increase in AFR regions.

**Table 2:** The regional adherence rates of cervical cancer screening in 2019, by age

Region	Adherence rate, %					
	20–29 years	30–39 years	40–49 years	50–59 years	60–69 years	Overall (20–69 years)
HICs	72.17 (62.58–80.09)	79.96 (71.97–86.12)	78.18 (69.74–84.78)	76.11 (67.20–83.21)	71.04 (61.22–79.22)	75.66 (66.74–82.81)
AMR	71.80 (62.11–79.83)	79.70 (71.60–85.95)	77.67 (69.07–84.41)	75.41 (66.32–82.68)	70.17 (60.19–78.55)	75.03 (65.95–82.35)
EUR	71.35 (61.58–79.47)	79.33 (71.14–85.67)	77.22 (68.52–84.07)	74.97 (65.80–82.35)	69.63 (59.57–78.11)	74.66 (65.51–82.06)
EMR	63.70 (53.05–73.16)	72.74 (63.15–80.60)	70.40 (60.44–78.74)	67.73 (57.41–76.57)	61.73 (50.91–71.51)	68.53 (58.41–77.16)
WPR	77.31 (68.67–84.13)	84.07 (77.20–89.15)	82.29 (74.90–87.87)	80.46 (72.55–86.51)	75.84 (66.84–83.01)	80.15 (72.23–86.25)
LMICs	19.14 (13.64–26.01)	28.15 (20.84–36.66)	27.29 (20.07–35.78)	26.99 (19.74–35.53)	23.60 (17.02–31.59)	24.91 (14.30–30.24)
AFR	4.33 (2.81–6.63)	6.52 (4.26–9.86)	5.86 (3.81–8.89)	5.20 (3.38–7.92)	4.07 (2.63–6.23)	5.28 (3.43–8.03)
AMR	23.09 (16.17–31.85)	31.54 (22.80–41.80)	29.02 (20.78–38.93)	26.51 (18.79–36.00)	21.77 (15.15–30.25)	26.81 (19.07–36.27)
SEAR	14.92 (10.11–21.47)	21.20 (14.70–29.58)	19.29 (13.28–27.17)	17.42 (11.90–24.77)	14.02 (9.46–20.29)	17.71 (12.14–25.10)
EUR	50.14 (39.30–60.97)	60.49 (49.58–70.45)	57.59 (46.59–67.89)	54.44 (43.42–65.04)	47.89 (37.14–58.85)	54.54 (43.63–65.04)
EMR	10.43 (6.94–15.39)	15.14 (10.24–21.81)	13.72 (9.23–19.90)	12.29 (8.22–17.96)	9.78 (6.49–14.48)	12.54 (8.41–18.28)
WPR	32.82 (23.91–43.16)	42.59 (32.25–53.61)	39.60 (29.62–50.53)	36.84 (27.24–47.62)	30.81 (22.24–40.94)	37.09 (27.52–47.77)
Worldwide	26.24 (20.19–33.25)	35.87 (28.45–44.02)	36.25 (28.81–44.41)	36.86 (29.28–45.11)	35.16 (27.78–43.19)	33.66 (23.34–39.30)
AFR	4.33 (2.81–6.63)	6.52 (4.26–9.86)	5.86 (3.81–8.89)	5.20 (3.38–7.92)	4.07 (2.63–6.23)	5.28 (3.43–8.03)
AMR	42.18 (34.17–50.65)	50.91 (42.43–59.56)	49.43 (41.03–58.01)	49.51 (41.14–57.96)	48.08 (39.63–56.50)	47.84 (39.52–56.36)
SEAR	14.92 (10.11–21.47)	21.20 (14.70–29.58)	19.29 (13.28–27.17)	17.42 (11.90–24.77)	14.02 (9.46–20.29)	17.71 (12.14–25.10)
EUR	61.01 (50.72–70.45)	69.82 (60.25–77.99)	68.30 (58.55–76.72)	66.28 (56.32–75.02)	60.04 (49.67–69.61)	65.36 (55.40–74.19)
EMR	14.20 (10.20–19.47)	20.49 (15.15–27.26)	19.08 (14.08–25.47)	16.06 (11.57–21.95)	12.48 (8.79–17.44)	16.99 (12.39–22.96)
WPR	37.68 (28.80–47.63)	46.97 (37.00–57.37)	45.16 (35.51–55.39)	42.25 (32.86–52.44)	37.78 (29.14–47.45)	42.39 (33.02–52.51)

*Abbreviations:* *AFR* African Region, *AMR* Region of the Americas, *EMR* Eastern Mediterranean Region, *EUR* European Region, *HICs* high-income countries, *LMICs* low and middle-income countries, *SEAR* South-East Asia Region, *WPR* Western Pacific Region

### National cervical cancer screening adherence

The national adherence rates of cervical cancer screening varied considerably, being as low as 11.70% (95% CI, 10.45–12.95%) in South Africa and as high as 82.57% (95% CI, 82.49–82.64%) in Denmark (Fig. S3). The adherence rates among women in the general population in the 28 countries with available data are reported in the Supplement (Table S9).

## Discussion

The current systematic analysis and modelling study based on 153 studies allowed for a comprehensive estimation of the global and regional cervical cancer screening adherence. It is also the first to extensively explore possible factors associated with screening adherence and to quantitatively synthesize their effect estimates among general women. The findings demonstrated that screening adherence was positively associated with being married, higher educational attainment, having healthcare, former smoking, physical activity, parity, and chronic disease, and negatively associated with obesity and current smoking. The global adherence rate for women aged 20–69 years in 2019 was 33.66%, with 75.66% in HICs and 24.91% in LMICs. Besides, the proportion of women who complied with regular screening ranged from less than 6% in AFR (LMIC) to 80% or higher in EUR (HIC).

Our results exhibit the global cervical cancer screening adherence is still low, falling far from the WHO target which states 70% of mid-adult women being screened [7]. Although most of the included studies used Pap test at 3-year interval, the effective screening methods include conventional cytology, liquid-based cytology, visual inspection with acetic acid, and HPV testing. Among them, HPV testing developed since 2000s, is currently recommended in priority due to its high sensitivity in detecting pre-neoplastic lesions, safe prolongation of screening intervals, and convenience of sampling [16, 17]. Moreover, cervical cancer screening might be performed together with other public health programs efficiently, e.g., the simultaneous scale-up of cervical cancer screening to adult women and HPV vaccination to adolescent girls, the combinative screening of cervical cancer and breast cancer, the conjunction of cervical cancer screening service with HIV prevention and treatment services, and the integration of cervical cancer screening into primary care service.

Furthermore, remarkable geographical variations in adherence were highlighted, particularly when comparing the adherence rates in HICs with those in LMICs. In general, HICs had high screening adherence and low cervical cancer incidence. Additionally, the incidence hits its ceiling at the age of 50 mainly contributed by the removal of precancerous lesions found by screening [18]. In contrast, LMICs usually had low adherence and tolerated a massive burden of cervical cancer with incidence increasing with age increase, accompanied by increasing time tendencies in incidence among typical LMICs [18, 19]. This disparity captures the significant obstacles that practitioners and policymakers face when striving to eliminate cervical cancer in regions with poor economic stability and heavy disease burden. It also underlines the critical need to scale up cervical cancer screening in LMICs. This is especially true in EMR, where the incidence of cervical cancer is lower than that of other LMICs, which may be explained by societal variables associated with sexual behavior [20, 21]. The low adherence observed in LMICs might be resulted from the relatively un-robust economic stability, limited medical infrastructure, prioritization of other public health problems, and poor cervical cancer screening programs at area level [7]. Adherence might be also hindered by some social determinants of health at individual level, including poor health literacy, insufficient awareness about screening benefit [22], lower education attainment [23], cultural and religious barriers (e.g., fear of losing virginity, stigma and embarrassment to screening, a lack of permission from husbands to have testing) [22, 24, 25]. Nevertheless, country-specific adherence may occasionally deviate from the regional estimate, mainly due to variations in cultural and behavioral factors, financial investment, policy support, national health care system, and local cervical cancer screening strategy.

Beyond the geographical disparities, there were noticeable differences in screening adherence by age, which has implications for incidence and mortality levels [18]. Our results showed that the adherence rate was relatively higher in women aged 30–39 and 40–49 years, who are also the screening-target population recommended by WHO [7].

With regard to marital status in demographic characteristics, this study showed that married or partnered women were more likely to undergo regular cervical cancer screening compared with unmarried or single women. One possible reason is that the most commonly used screening method, Pap test, was often provided by pre-and post-natal services to married women [26]. Women with partners were also more apt to take healthy behaviors through spousal monitoring and then received more frequently obstetric and gynecological care including cervical cancer screening [27].

Several key social determinants of health, including education level and healthcare were found to be associated with adherence to cervical cancer screening. Our results showed that women with a higher education level had a higher adherence. These higher-educated women have more health-related knowledge and better health literacy to be more aware of their health risks [28], which may actively influence individual screening participation, particularly in opportunistic screening programs than their lower-educated counterparts [29]. Moreover, education is closely related to an individual’s socioeconomic status [30]. Women with higher education usually have better socioeconomic status, and the latter further leads to better access to health-related information and healthcare resources [24].

This study explored unhealthy lifestyle factors negatively associated with adherence to cervical cancer screening. Obese women were 15% less likely than those non-obese to get regular screening. One potential reason for this is that obese women are more likely to have negative opinions about their appearance, and a reluctance to obtain pelvic examinations due to higher anxiety about physical privacy, weight embarrassment, and increased pain and discomfort from screening [31]. Smoking is another well-recognized lifestyle risk factor. Our study showed that smokers were 36% less likely than former/never smokers, but ex-smokers were 20% more likely than never smokers to comply with cervical examinations. Smokers show exaggerated optimism about their health status, which may lead to an overall reduced acceptance of health-promoting practices [32]. Ex-smokers, on the other hand, typically have enhanced overall health awareness and are especially driven to embrace a healthy lifestyle [33].

It is worth noting that cervical cancer screening has been paid much more attention recently. Lemp and colleagues successfully estimated the lifetime prevalence (having ever undergone a screening test) of cervical cancer screening in women aged 30–49 years in 55 LMICs [34], and Bruni and colleagues estimated the global, regional, and national age-specific coverage of cervical cancer screening also for women aged 30–49 years [35]. However, screening adherence is a rather complex concept, which could be influenced markedly by both individual factors (e.g., demographic characteristics, and behavioral and educational factors) and public factors (e.g., policy advocacy and health care resource support). In the current study, we systematically estimated global and regional adherence rates of cervical cancer screening in women across a broad age range of 20–69 years. Our included studies were all surveys, which serve a reliable source to reflect the uptake of screening implementation from individual perspective. Further, we extensively examined possible associated factors which are meaningful for policymakers, community leaders, and health practitioners to provide more customized methods. Our study is a meaningful supplement to the current understanding of cervical cancer screening.

COVID-19 pandemic has brought new challenges to cervical cancer screening, such as reduced access to health-care services, the delay or suspension of screening, and accelerated health inequities, especially among women living in LMICs [36]. Therefore, our study was timed before the pandemic in order to reflect the general level. Nonetheless, the current findings in screening adherence status are very important for perfecting post-pandemic efforts to achieve screening goals, and the COVID-19 crisis may serve as an alarm bell and remind us to rethink and reform the availability and convenience of screening.

This study has several limitations. First, although we examined eleven associated factors for cervical cancer screening adherence, there is still a lack of data on several other factors such as the age of first sexual activity, sexual risk behaviors, and oral hormonal contraception. The lack of information on these factors for screening restricted our ability to conduct a more comprehensive meta-analysis assessment for this condition. Second, we only accounted for age and SDI in different geographical regions based on the model. Additionally, there is evidence that sexually transmittable infections (e.g., HIV) can lead to a notably increased risk of cervical cancer [4], but we were unable to account for these effects in our regional modeling due to a lack of relevant data. This limitation might have resulted in partially biased adherence for each geographical region. Third, the available data is still relatively limited in the world, and therefore the estimations might not reflect the actual situation of countries with scarce or no data. This may be the case for Asia and Africa where data was especially limited, and it might ultimately lead to a relatively rough global adherence estimation. Finally, our screening adherence estimates may also be influenced by the delayed reporting on the results to the screened women, especially in LMICs. Further efforts will be needed in continual financial and human supports, screening practice scale-up, high-quality and timely reporting, and policy optimization and corresponding strategy perfection to improve cervical cancer screening for at-risk women worldwide.

## Conclusions

Cervical cancer screening adherence remained low globally, exhibiting geographical discrepancy with HICs higher than LMICs. Further implementations of screening programs can comprehensively consider the local economy, social benefits, and demographic structure, and provide adequately customized methods to vulnerable or underserved women who are obese, currently smoke, do not have a partner, have low educational attainment, and do not have access to healthcare. Our study is expected to prompt further targeted scale-up of cervical cancer screening and accelerate the declines in cervical cancer and eventual elimination substantially.

## Supplementary Information

**Additional file 1.** PRISMA 2020 Checklist.

**Additional file 2.** GATHER Checklist.

**Additional file 3: Table S1.** Searching strategy to identify studies focusing on adherence to cervical cancer screening. **Table S2.** The time lag between investigation and publication in the included studies reporting the adherence rate of cervical cancer screening (Mean = 5.40 years). **Table S3.** Quality assessment scale for rating the risk of bias. **Table S4.** Univariable and multivariable meta-regression models of cluster-level factors related to the adherence rate of cervical cancer screening (logit form). **Table S5.** Reference list of the included studies. **Table S6.** Basic characteristics of the included studies. **Table S7.** Quality scores for assessing the risk of bias for the included studies. **Table S8.** Meta-analyses of associated factors for adherence to cervical cancer screening. **Table S9.** Pooled or reported adherence rate of cervical cancer screening in 28 countries with available data. **Fig. S1.** Geographic distribution of the included studies. **Fig. S2.** The estimated relation between age and adherence rates of cervical cancer screening based on informative data points from the included articles, by SDI. **Fig. S3.** Adherence rate of cervical cancer screening by country.

## Data Availability

All data analyzed during this study are included in this manuscript and its supplementary information files. Additional data are available from the corresponding author on request.

## Abbreviations

AFR: African Region
AMR: Region of the Americas
BMI: Body-mass index
CI: Confidence interval
EMR: Eastern Mediterranean Region
EUR: European Region
GATHER: Guidelines for Accurate and Transparent Health Estimates Reporting
HICs: High-income countries
HIV: Human immunodeficiency virus
HPV: Human papillomavirus
LMICs: Low and middle-income countries
ORs: Odds ratios
PRISMA: Preferred Reporting Items for Systematic Reviews and Meta-Analysis
SDH: Social determinants of health
SDI: Socio-demographic Index
SEAR: Southeast Asia Region
STROBE: Strengthening the Reporting of Observational studies in Epidemiology
WB: World Bank
WHO: World Health Organization
WPR: Western Pacific Region
