# Supplementary material for: Associated factors and global adherence of cervical cancer screening in 2019: a systematic analysis and modelling study
Source: Global Health. 2022 Dec 9;18:101. doi: 10.1186/s12992-022-00890-w (PMC9733311; doi:10.1186/s12992-022-00890-w)
Supplement: Supplementary file 2 — Additional file 2. GATHER Checklist. [file 12992_2022_890_MOESM2_ESM.pdf]

GATHER Checklist

Item number	Checklist item	Location where item is reported
**Objectives and funding**		
1	Define the indicator(s), populations (including age, sex, and geographic entities), and time period(s) for which estimates were made.	4
2	List the funding sources for the work.	18
**Data inputs**		
For all data inputs from multiple sources that are synthesised as part of the study:		
3	Describe how the data were identified and how the data were accessed.	5-6
4	Specify the inclusion and exclusion criteria. Identify all ad-hoc exclusions.	5
5	Provide information about all included data sources and their main characteristics. For each data source used, report reference information or contact name/institution, population represented, data collection method, year(s) of data collection, sex and age range, diagnostic criteria or measurement method, and sample size, as relevant.	Table S6
6	Identify and describe any categories of input data that have potentially important biases (eg, based on characteristics listed in item 5).	Table S3, Table S7
For data inputs that contribute to the analysis but were not synthesised as part of the study:		
7	Describe and give sources for any other data inputs.	6-7
For all data inputs:		
8	Provide all data inputs in a file format from which data can be efficiently extracted (eg, a spreadsheet rather than a PDF), including all relevant meta-data listed in item 5. For any data inputs that cannot be shared because of ethical or legal reasons, such as third-party ownership, provide a contact name or the name of the institution that retains the right to the data.	NA
**Data analysis**		
9	Provide a conceptual overview of the data analysis method. A diagram may be helpful.	7-8
10	Provide a detailed description of all steps of the analysis, including mathematical formulae. This description should cover, as relevant, data cleaning, data pre-processing, data adjustments and weighting of data sources, and mathematical or statistical model(s).	7-8
11	Describe how candidate models were evaluated and how the final model(s) were selected.	7-8
12	Provide the results of an evaluation of model performance, if done, as well as the results of any relevant sensitivity analysis.	NA

Item number	Checklist item	Location where item is reported
13	Describe methods of calculating uncertainty of the estimates. State which sources of uncertainty were, and were not, accounted for in the uncertainty analysis.	NA
14	State how analytical or statistical source code used to generate estimates can be accessed.	NA
**Results and discussion**		
15	Provide published estimates in a file format from which data can be efficiently extracted.	Table S6
16	Report a quantitative measure of the uncertainty of the estimates (eg, uncertainty intervals).	10-11, Table 2
17	Interpret results in light of existing evidence. If updating a previous set of estimates, describe the reasons for changes in estimates.	14-15
18	Discuss limitations of the estimates. Include a discussion of any modelling assumptions or data limitations that affect interpretation of the estimates.	15-16
