# Supplementary material for: Associated factors and global adherence of cervical cancer screening in 2019: a systematic analysis and modelling study
Source: Global Health. 2022 Dec 9;18:101. doi: 10.1186/s12992-022-00890-w (PMC9733311; doi:10.1186/s12992-022-00890-w)
Supplement: Supplementary file 3 — Additional file 3: Table S1. Searching strategy to identify studies focusing on adherence to cervical cancer screening. Table S2. The time lag between investigation and publication in the included studies reporting the adherence rate of cervical cancer screening (Mean = 5.40 years). Table S3. Quality assessment scale for rating the risk of bias. Table S4. Univariable and multivariable meta-regression models of cluster-level factors related to the adherence rate of cervical cancer screening (logit form). Table S5. Reference list of the included studies. Table S6. Basic characteristics of the included studies. Table S7. Quality scores for assessing the risk of bias for the included studies. Table S8. Meta-analyses of associated factors for adherence to cervical cancer screening. Table S9. Pooled or reported adherence rate of cervical cancer screening in 28 countries with available data. Fig. S1. Geographic distribution of the included studies. Fig. S2. The estimated relation between age and adherence rates of cervical cancer screening based on informative data points from the included articles, by SDI. Fig. S3. Adherence rate of cervical cancer screening by country. [file 12992_2022_890_MOESM3_ESM.pdf]

Table S1 Searching strategy to identify studies focusing on adherence to cervical cancer screening

Table S2 The time lag between investigation and publication in the included studies reporting the adherence rate of cervical cancer screening (Mean=5.40 years)

Table S3 Quality assessment scale for rating the risk of bias

Table S4 Univariable and multivariable meta-regression models of cluster-level factors related to the adherence rate of cervical cancer screening (logit form)

Table S5 Reference list of the included studies

Table S6 Basic characteristics of the included studies

Table S7 Quality scores for assessing the risk of bias for the included studies

Table S8 Meta-analyses of associated factors for adherence to cervical cancer screening

Table S9 Pooled or reported adherence rate of cervical cancer screening in 28 countries with available data

Figure S1 Geographic distribution of the included studies

Figure S2 The estimated relation between age and adherence rates of cervical cancer screening based on informative data points from the included articles, by SDI

Figure S3 Adherence rate of cervical cancer screening by country

Table S1 Searching strategy to identify studies focusing on adherence to cervical cancer screening

Database	Accessed date	Search terms
PubMed	23th June, 2021	(cancer*[Title/Abstract] OR neoplas*[Title/Abstract] OR carcinoma*[Title/Abstract] OR tumor*[Title/Abstract] OR tumour*[Title/Abstract]) AND (cervi*[Title/Abstract] OR uterine neck[Title/Abstract] OR neck of uterus[Title/Abstract]) AND (screen*[Title/Abstract] OR early detection[Title/Abstract] OR Papanicolaou test*[Title/Abstract] OR Pap test*[Title/Abstract] OR Pap smear*[Title/Abstract] OR vaginal smear*[Title/Abstract] OR HPV test*[Title/Abstract] OR cervical cytology[Title/Abstract] OR colposcop*[Title/Abstract]) AND (adhere*[Title/Abstract] OR complianc*[Title/Abstract] OR participation*[Title/Abstract] OR attendan*[Title/Abstract]) Filters: from 1000/1/1-2021/6/23
Web of Science	23th June, 2021	TOPIC: (Cancer* OR neoplas* OR carcinoma* OR tumor* OR tumour*) AND TOPIC: (cervi* OR uterine neck OR neck of uterus) AND TOPIC: (screen* OR early detection OR Papanicolaou test* OR Pap test* OR Pap smear* OR vaginal smear* OR HPV test* OR cervical cytology OR colposcop*) AND TOPIC: (adhere* OR complianc* OR participation* OR attendan*)
Embase	23th June, 2021	#1 (cancer*:ab,ti OR neoplas*:ab,ti OR carcinoma*:ab,ti OR tumor*:ab,ti OR tumour*:ab,ti) AND (cervi*:ab,ti OR 'uterine neck':ab,ti OR 'neck of uterus':ab,ti) AND (screen*:ab,ti OR 'early detection':ab,ti OR 'papanicolaou test*':ab,ti OR 'pap test*':ab,ti OR 'pap smear*':ab,ti OR 'vaginal smear*':ab,ti OR 'hpv test*':ab,ti OR 'cervical cytology':ab,ti OR colposcop*:ab,ti) AND (adhere*:ab,ti OR complianc*:ab,ti OR participation*:ab,ti OR attendan*:ab,ti) AND [1966-2021]/py<br>#1 AND ('article'/it OR 'article in press'/it OR 'review'/it)
China National Knowledge Infrastructure	23th June, 2021	TOPIC: (癌 OR 瘤) AND (宫颈 OR 子宫颈) AND (筛查 OR 筛检 OR 巴氏试验 OR 巴氏涂片 OR 细胞学检查 OR HPV 检查) AND (依从 OR 参与 OR 行为)
Wanfang data	23th June, 2021	TOPIC: (癌 OR 瘤) AND (宫颈 OR 子宫颈) AND (筛查 OR 筛检 OR 巴氏试验 OR 巴氏涂片 OR 细胞学检查 OR HPV 检查) AND (依从 OR 参与 OR 行为)
Google Web	23th June, 2021	"cervical cancer" AND screening AND (adherence OR participation OR attendance) -pubmed
ProQuest Dissertations & Theses Global	23th June, 2021	noft("cervical cancer") AND noft(screening) AND noft(adherence OR participation OR attendance)

Table S2 The time lag between investigation and publication in the included studies reporting the adherence rate of cervical cancer screening (Mean = 5.40 years)

Study ID	Study	Year published	Year investigated	Time-lag (year)
1	Cohen MM, et al. (1992)	1992	1983	9
2	Lantz PM, et al. (1997)	1997	1993	4
3	Risendal B, et al. (1999)	1999	1996	3
4	Fernandez ME, et al. (1999)	1999	1996	3
5	Rohlfs I, et al. (1999)	1999	1992	7
6	Taylor VM, et al. (1999)	1999	1998	1
7	Maxwell AE, et al. (2000)	2000	1996	4
8	Ramirez AG, et al. (2000)	2000	1994	6
9	Maxwell CJ, et al. (2001)	2001	1997	4
10	Do HH, et al. (2001)	2001	1999	2
11	Jirojwong S, et al. (2001)	2001	1998	3
12	Lee J, et al. (2002)	2002	1998	4
13	Cyrus-David MS, et al. (2002)	2002	1996	6
14	Amonkar MM, et al. (2002)	2002	1997	5
15	Fernandez-Esquer ME, et al. (2003)	2003	1992	11
16	Selvin E, et al. (2003)	2003	1992	11
17	Juon HS, et al. (2003)	2003	2000	3
18	Miedema BB, et al. (2003)	2003	2002	1
19	Hou SI, et al. (2003)	2003	1999	4
20	Luengo Matos S, et al. (2003)	2003	2000	3
21	Taylor VM, et al. (2004)	2004	2002	2
22	Coronado GD, et al. (2004)	2004	1998	6
23	Insinga RP, et al. (2004)	2004	2000	4
24	Dettenborn L, et al. (2004)	2004	NA	NA
25	Borrayo EA, et al. (2004)	2004	NA	NA
26	Bazargan M, et al. (2004)	2004	1999	5
27	Cho IS, et al. (2004)	2004	2000	4
28	Hoyo C, et al. (2005)	2005	2001	4
29	De Alba I，et al(2005)	2005	2001	4
30	Kwak MS, et al. (2005)	2005	2004	1
31	Giorgi Rossi P, et al. (2006)	2006	2004	2
32	Carruth AK, et al. (2006)	2006	NA	NA

Study ID	Study	Year published	Year investigated	Time-lag (year)
33	Liao CC, et al. (2006)	2006	2000	6
34	Lee-Lin F, et al. (2007)	2007	NA	NA
35	Chen LS, et al. (2007)	2007	2002	5
36	Gao W, et al. (2008)	2008	2007	1
37	Tsui J et al. (2008)	2008	2003	5
38	Puig-Tintore LM, et al. (2008)	2008	2005	3
39	Ginde AA, et al. (2008)	2008	2007	1
40	Tung WC, et al. (2008)	2008	NA	NA
41	Nelson W, et al. (2009)	2009	2005	4
42	Ma GX, et al. (2009)	2009	2006	3
43	Dimitrakaki C, et al. (2009)	2009	2006	3
44	Amankwah E, et al. (2009)	2009	2003	6
45	Taylor VM, et al. (2009)	2009	2005	4
46	Sun YG, et al. (2009)	2009	NA	NA
47	Martin-Lopez R, et al. (2010)	2010	2007	3
48	Zhou J, et al. (2010)	2010	2000	10
49	Byrne MM, et al. (2010)	2010	2007	3
50	Lopez-de-Andres A, et al. (2010)	2010	2005	5
51	Lim JW, et al. (2010)	2010	2007	3
52	Paskett ED, et al. (2010)	2010	2006	4
53	Lee K, et al. (2010)	2010	2005	5
54	Meng RL, et al. (2010)	2010	NA	NA
55	Wang CF, et al. (2010)	2010	2007	3
56	Lawsin C, et al. (2011)	2011	NA	NA
57	Cheung NW, et al. (2011)	2011	2004	7
58	Pons-Vigues M, et al. (2011)	2011	NA	NA
59	Tekkel M, et al. (2011)	2011	2004	7
60	Nuno T, et al. (2011)	2011	2006	5
61	Hansen BT, et al. (2011)	2011	2005	6
62	MacLaughlan SD, et al. (2011)	2011	2006	5
63	Vakfari A, et al. (2011)	2011	2007	4
64	Acikgoz A, et al. (2011)	2011	2009	2
65	Lee M, et al. (2011)	2011	2008	3
66	Liang Q, et al. (2011)	2011	NA	NA
67	Nguyen-Truong CK, et al. (2012)	2012	2010	2
68	Worthington C, et al. (2012)	2012	2000	12

Study ID	Study	Year published	Year investigated	Time-lag (year)
69	Gonzalez P, et al. (2012)	2012	2007	5
70	Damiani G, et al. (2012)	2012	2005	7
71	Martin-Lopez R, et al. (2012)	2012	2009	3
72	Muus KJ, et al. (2012)	2012	2005	7
73	Olesen SC, et al. (2012)	2012	NA	NA
74	Macedo A, et al. (2012)	2012	2010	2
75	Ko KD, et al. (2012)	2012	2005	7
76	Azerkan F, et al. (2012)	2012	1999	13
77	Aminisani N, et al. (2012)	2012	2003	9
78	Nuno T, et al. (2012)	2012	2008	4
79	Shelton RC, et al. (2012)	2012	NA	NA
80	Ashok M, et al. (2012)	2012	2008	4
81	Leone LA, et al. (2012)	2012	NA	NA
82	Shippee ND, et al. (2012)	2012	2007	5
83	Aminisani N, et al. (2012)	2012	2002	10
84	Raz R, et al. (2012)	2012	2007	5
85	Borges MF, et al. (2012)	2012	2008	4
86	Almeida CM, et al. (2013）	2013	2008	5
87	Morrison TB, et al. (2013）	2013	2007	6
88	Oussaid N, et al. (2013）	2013	2008	5
89	Cerigo H, et al. (2013）	2013	2005	8
90	Augusto EF, et al. (2013）	2013	2010	3
91	Khadilkar A, et al. (2013）	2013	2008	5
92A	Lee M, et al. (2013）	2013	1998	15
92B	Lee M, et al. (2013）	2013	2001	12
92C	Lee M, et al. (2013）	2013	2005	8
92D	Lee M, et al. (2013）	2013	2008	5
92E	Lee M, et al. (2013）	2013	2010	3
93	Suh M, et al. (2013）	2013	2012	1
94	Hernandez CM, et al. (2014)	2014	2011	3
95	Tsai RJ, et al. (2014)	2014	2010	4
96	Adonis L, et al. (2014)	2014	2009	5
97	Moudatsou MM, et al. (2014)	2014	2007	7
98	Kristensson JH, et al. (2014)	2014	2005	9

Study ID	Study	Year published	Year investigated	Time-lag (year)
99A	Arbyn M, et al. (2014)	2014	2005	9
99B	Arbyn M, et al. (2014)	2014	2003	11
99C	Arbyn M, et al. (2014)	2014	1999	15
99D	Arbyn M, et al. (2014)	2014	1997	17
100	Calo WA (2014)	2014	2010	4
101	Documet P, et al. (2015)	2015	2010	5
102	Guo F, et al. (2015)	2015	2013	2
103	Visanuyothin S, et al. (2015)	2015	2012	3
104	Ricardo-Rodrigues I, et al. (2015)	2015	2012	3
105	Miranda-Diaz C, et al. (2015)	2015	2012	3
106	Gyulai A, et al. (2015)	2015	2008	7
107	Richard A, et al. (2015)	2015	2012	3
108	Schoofs J, et al. (2015)	2015	2012	3
109	Ostensson E, et al. (2015)	2015	2013	2
110	Barbadoro P, et al. (2015)	2015	2005	10
111	Du MX, et al. (2015)	2015	NA	NA
112	Hirth JM, et al. (2016)	2016	2012	4
113	Crawford A, et al. (2016)	2016	2014	2
114	Shneyderman Y, et al. (2016)	2016	2003	13
114	Shneyderman Y, et al. (2016)	2016	2007	9
115	Valdovinos C, et al. (2016)	2016	2010	9
116	Urrutia MT, et al. (2016)	2016	NA	NA
117	Bianco A, et al. (2017)	2017	2012	6
118	Comparetto C, et al. (2017)	2017	2006	11
119A	Parekh N, et al. (2017)	2017	2007	10
119B	Parekh N, et al. (2017)	2017	2010	7
120	Leinonen MK, et al. (2017)	2017	2010	7
121	White A, et al. (2017)	2017	2015	2
122	Tung WC, et al. (2017)	2017	2015	2
123	Cadet TJ, et al. (2017)	2017	2008	9
124	Lofters AK, et al. (2017)	2017	2015	2
125	Zorogastua K, et al. (2017)	2017	NA	NA
126	Chang HK, et al. (2017)	2017	2011	6
127	Cofie LE, et al. (2018)	2018	2015	3
128	Luque JS, et al. (2018)	2018	2014	4
129	Rendle KA, et al. (2018)	2018	2013	5

Study ID	Study	Year published	Year investigated	Time-lag (year)
130	Petrelli A, et al. (2018)	2018	2013	5
131	Han J, et al. (2018)	2018	2017	1
132	Restivo V, et al. (2018)	2018	2016	2
133	Luque JS, et al. (2018)	2018	2016	2
134A	Petkeviciene J, et al. (2018)	2018	2006	12
134B	Petkeviciene J, et al. (2018)	2018	2008	10
134C	Petkeviciene J, et al. (2018)	2018	2010	8
134D	Petkeviciene J, et al. (2018)	2018	2012	6
134E	Petkeviciene J, et al. (2018)	2018	2014	4
135	Moreno PI, et al. (2019)	2019	2010	9
136	Lucas-Wright A, et al. (2019)	2019	2014	5
137	MacLaughlin KL, et al. (2019)	2019	2011	8
138	Kaso M, et al. (2019)	2019	2013	6
139	Perez CM, et al. (2020)	2020	NA	NA
140	Charkhchi P, et al. (2020)	2020	2016	4
141	Abar B, et al. (2020)	2020	NA	NA
142	Maxwell AE, et al. (2020)	2020	2017	3
143	Zamorano-Leon JJ, et al. (2020)	2020	2017	3
144A	Garrido CO, et al. (2020)	2020	2008	12
144B	Garrido CO, et al. (2020)	2020	2010	10
144C	Garrido CO, et al. (2020)	2020	2012	8
144D	Garrido CO, et al. (2020)	2020	2014	6
144E	Garrido CO, et al. (2020)	2020	2016	4
144F	Garrido CO, et al. (2020)	2020	2018	2
145	Wilding S, et al. (2020)	2020	NA	NA
146	Franck JE, et al. (2020)	2020	2014	6
147	Lee HY, et al. (2020)	2020	NA	NA
148	Barrera-Castillo M, et al. (2020)	2020	2012	8
149	Deguara M, et al. (2020)	2020	2017	3
150	dos Santos NLF, et al. (2020)	2020	2018	2
151	Malone C, et al. (2021)	2021	2015	6
152	Pelullo CP, et al. (2021)	2021	2019	2
153	Ishii K, et al. (2021)	2021	2016	5

Abbreviations: NA, not available.

Table S3 Quality assessment scale for rating the risk of bias

Bias type	Low risk (score=2)	Moderate risk (score=1)	High risk (score=0)
Selection (sample population)	1) Sample from the general population, not a select group; 2) Consecutive unselected population; 3) Rationale for case and control selection explained.	1) Sample selected from large population but selection criteria not defined; 2) Sample selection ambiguous but may be representative; 3) Rationale for cases and controls not explained; 4) Eligibility criteria not explained; 5) Analysis to adjust for sampling strategy bias.	1) Highly select population making it difficult to generalise finding; 2) Sample selection ambiguous and sample unlikely to be representative.
Selection (sample size)	1) Sample size calculation performed and adequate.	1) Sample size calculation performed and reasons for not meeting sample size given; 2) Sample size calculation not performed but all eligible persons studied.	1) Sample size estimation unclear or only sub-sample studied.
Selection (participation)	1) High response rate (>85%).	1) Moderate response rate (70-85%).	1) Low response rate (<70%); 2) Response rate not reported.
Performance bias (outcome assessment)	1) Assessment from independent objective assessment or individual secure records; 2) Assessment from administrative database or register (hospital record or linkage record).	1) Assessment from self-report	1) Assessment from non-validated data or generic estimate from the overall population.
Performance bias (analytical methods to control for bias)	1) Analysis appropriate for the type of sample (subgroup analysis / regression etc.).	1) Analysis does not account for common adjustment.	1) Data confusing.

Table S4 Univariable and multivariable meta-regression models of cluster-level factors related to the adherence rate of cervical cancer screening (logit form)

**Moderator**		**No. of studies**	**No. of data points**	**β [b]**	**95 % CI**	***P* value**
Age (per year increase)		87	327	-0.0070	(-0.0072, -0.0068)	<0.0001
Year investigated (per year increase) [a]		87	327	0.0427	(0.0419, 0.0436)	<0.0001
SDI (per 1% increase) [a]		87	327	0.1014	(0.0990, 0.1038)	<0.0001
Area [a]		87				
	Other		283	Reference		
	Urban		7	0.1487	(0.0960, 0.2014)	<0.0001
	Rural		37	-1.0555	(-1.0919, -1.0190)	<0.0001
WHO Region [a]		87				
	EUR		92	Reference		
	AMR		160	0.5669	(-0.2249, 1.3586)	0.1605
	WPR & SEAR		75	-0.5198	(-1.2091, 0.1695)	0.1394

Abbreviations: AMR, Region of the Americas; CI, confidence interval; EUR, European Region; SDI, Socio-demographic Index; SEAR, South-East Asia Region; WHO, World Health Organization; WPR, Western Pacific Region.

[a] adjusted for age; [b] coefficients represent log odds ratios (ORs).

Table S5 Reference list of the included studies

Study ID	Reference
1	Cohen MM, Roos NP, MacWilliam L, Wajda A. Assessing physicians' compliance with guidelines for Papanicolaou testing. Med Care. Jun 1992;30(6):514-28. doi:10.1097/00005650-199206000-00005.
2	Lantz PM, Weigers ME, House JS. Education and income differentials in breast and cervical cancer screening. Policy implications for rural women. Med Care. Mar 1997;35(3):219-36. doi:10.1097/00005650-199703000-00004.
3	Risendal B, DeZapien J, Fowler B, Papenfuss M, Giuliano A. Pap smear screening among urban Southwestern American Indian women. Prev Med. Dec 1999;29(6 Pt 1):510-8. doi:10.1006/pmed.1999.0565.
4	Fernandez ME, DeBor M, Candreia MJ, Wagner AK, Stewart KR. Evaluation of ENCOREplus. A community-based breast and cervical cancer screening program. Am J Prev Med. Apr 1999;16(3 Suppl):35-49. doi:10.1016/s0749-3797(98)00145-7.
5	Rohlfs I, Borrell C, Pasarin MI, Plasencia A. The role of sociodemographic factors in preventive practices - The case of cervical and breast cancer. Eur J Public Health. Dec 1999;9(4):278-284. doi:DOI 10.1093/eurpub/9.4.278.
6	Taylor VM, Schwartz SM, Jackson JC, et al. Cervical cancer screening among Cambodian-American women. Cancer Epidemiol Biomarkers Prev. Jun 1999;8(6):541-6.
7	Maxwell AE, Bastani R, Warda US. Demographic predictors of cancer screening among Filipino and Korean immigrants in the United States. Am J Prev Med. Jan 2000;18(1):62-8. doi:10.1016/s0749-3797(99)00110-5.
8	Ramirez AG, Suarez L, McAlister A, et al. Cervical cancer screening in regional Hispanic populations. Am J Health Behav. May-Jun 2000;24(3):181-192. doi:Doi 10.5993/Ajhb.24.3.3.
9	Maxwell CJ, Bancej CM, Snider J, Vik SA. Factors important in promoting cervical cancer screening among Canadian women: findings from the 1996-97 National Population Health Survey (NPHS). Can J Public Health. Mar-Apr 2001;92(2):127-33.
10	Do HH, Taylor VM, Yasui Y, Jackson JC, Tu SP. Cervical cancer screening among Chinese immigrants in Seattle, Washington. J Immigr Health. Jan 2001;3(1):15-21. doi:10.1023/A:1026606401164.
11	Jirojwong S, Maclennan R, Manderson L. Health beliefs and Pap smears among Thai women in Brisbane, Australia. Asia Pac J Public Health. 2001;13(1):20-3. doi:10.1177/101053950101300105.
12	Lee J, Seow A, Ling SL, Peng LH. Improving adherence to regular pap smear screening among Asian women: a population-based study in Singapore. Health Educ Behav. Apr 2002;29(2):207-18. doi:10.1177/109019810202900206.
13	Cyrus-David MS, Michielutte R, Paskett ED, D'Agostino R, Jr., Goff D. Cervical cancer risk as a predictor of Pap smear use in rural North Carolina. J Rural Health. Winter 2002;18(1):67-76. doi:10.1111/j.1748-0361.2002.tb00878.x.
14	Amonkar MM, Madhavan S. Compliance rates and predictors of cancer screening recommendations among Appalachian women. J Health Care Poor Underserved. Nov 2002;13(4):443-60. doi:10.1353/hpu.2010.0582.
15	Fernandez-Esquer ME, Espinoza P, Ramirez AG, McAlister AL. Repeated Pap smear screening among Mexican-American women. Health Educ Res. Aug 2003;18(4):477-87. doi:10.1093/her/cyf037.

Study ID	Reference
16	Selvin E, Brett KM. Breast and cervical cancer screening: sociodemographic predictors among White, Black, and Hispanic women. Am J Public Health. Apr 2003;93(4):618-23. doi:10.2105/ajph.93.4.618.
17	Juon HS, Seung-Lee C, Klassen AC. Predictors of regular Pap smears among Korean-American women. Prev Med. Dec 2003;37(6 Pt 1):585-92. doi:10.1016/j.ypmed.2003.09.006.
18	Miedema BB, Tatemichi S. Breast and cervical cancer screening for women between 50 and 69 years of age: what prompts women to screen? Womens Health Issues. Sep-Oct 2003;13(5):180-4. doi:10.1016/s1049-3867(03)00039-2.
19	Hou SI, Fernandez ME, Baumler E, Parcel GS, Chen PH. Correlates of cervical cancer screening among women in Taiwan. Health Care Women Int. May-Jun 2003;24(5):384-98. doi:10.1080/07399330390212171.
20	Luengo Matos S, Munoz van den Eynde A. Use of pap smear for cervical cancer screening and factors related with its use in Spain. Aten Primaria. Mar 31 2004;33(5):229-34. doi:10.1016/s0212-6567(04)79407-x.
21	Taylor VM, Yasui Y, Burke N, et al. Pap testing adherence among Vietnamese American women. Cancer Epidemiol Biomarkers Prev. Apr 2004;13(4):613-9.
22	Coronado GD, Thompson B, Koepsell TD, Schwartz SM, McLerran D. Use of Pap test among Hispanics and non-Hispanic whites in a rural setting. Prev Med. Jun 2004;38(6):713-22. doi:10.1016/j.ypmed.2004.01.009.
23	Insinga RP, Glass AG, Rush BB. Pap screening in a U.S. health plan. Cancer Epidemiol Biomarkers Prev. Mar 2004;13(3):355-60.
24	Dettenborn L, DuHamel K, Butts G, Thompson H, Jandorf L. Cancer fatalism and its demographic correlates among African American and Hispanic women: Effects on adherence to cancer screening. J Psychosoc Oncol. 2004;22(4):47-60. doi:10.1300/J077v22n04_03.
25	Borrayo EA, Thomas JJ, Lawsin C. Cervical cancer screening among Latinas: the importance of referral and participation in parallel cancer screening behaviors. Women Health. 2004;39(2):13-29. doi:10.1300/J013v39n02_02.
26	Bazargan M, Bazargan SH, Farooq M, Baker RS. Correlates of cervical cancer screening among underserved Hispanic and African-American women. Prev Med. Sep 2004;39(3):465-73. doi:10.1016/j.ypmed.2004.05.003
27	Cho IS, Park YS. A study on regular cervical cancer screening behavior among middle-aged women. Taehan Kanho Hakhoe Chi. Feb 2004;34(1):141-9. doi:10.4040/jkan.2004.34.1.141.
28	Hoyo C, Yarnall KS, Skinner CS, Moorman PG, Sellers D, Reid L. Pain predicts non-adherence to pap smear screening among middle-aged African American women. Prev Med. Aug 2005;41(2):439-45. doi:10.1016/j.ypmed.2004.11.021.
29	De Alba I, Ngo-Metzger Q, Sweningson JM, Hubbell FA. Pap smear use in California: are we closing the racial/ethnic gap? Prev Med. Jun 2005;40(6):747-55. doi:10.1016/j.ypmed.2004.09.018.
30	Kwak MS, Park EC, Bang JY, Sung NY, Lee JY, Choi KS. Factors associated with cancer screening participation, Korea. J Prev Med Public Health. Nov 2005;38(4):473-81.
31	Giorgi Rossi P, Esposito G, Brezzi S, Brachini A, Raggi P, Federici A. Estimation of Pap-test coverage in an area with an organised screening program: challenges for survey methods. BMC Health Serv Res. Mar 17 2006;6:36. doi:10.1186/1472-6963-6-36.

Study ID	Reference
32	Carruth AK, Browning S, Reed DB, Skarke L, Sealey L. The impact of farm lifestyle and health characteristics: cervical cancer screening among southern farmwomen. Nurs Res. Mar-Apr 2006;55(2):121-7. doi:10.1097/00006199-200603000-00007.
33	Liao CC, Wang HY, Lin RS, Hsieh CY, Sung FC. Addressing Taiwan's high incidence of cervical cancer: factors associated with the Nation's low compliance with Papanicolaou screening in Taiwan. Public Health. Dec 2006;120(12):1170-6. doi:10.1016/j.puhe.2006.07.028.
34	Lee-Lin F, Pett M, Menon U, et al. Cervical cancer beliefs and pap test screening practices among Chinese American immigrants. Oncol Nurs Forum. Nov 2007;34(6):1203-9. doi:10.1188/07.ONF.1203-1209.
35	Chen LS, Huang N, Tsay JH, et al. Screening for cervical cancer among female physicians and their relatives in Taiwan: a population-based comparative study. Prev Med. Jun 2007;44(6):531-5. doi:10.1016/j.ypmed.2007.02.001.
36	Gao W, Desouza R, Paterson J, Lu T. Factors affecting uptake of cervical cancer screening among Chinese women in New Zealand. Int J Gynaecol Obstet. Oct 2008;103(1):76-82. doi:10.1016/j.ijgo.2008.04.025.
37	Tsui J, Tanjasiri SP. Cervical cancer screening among Thai women in Northern California. J Womens Health (Larchmt). Apr 2008;17(3):393-401. doi:10.1089/jwh.2007.0427.
38	Puig-Tintore LM, Castellsague X, Torne A, et al. Coverage and factors associated with cervical cancer screening: results from the AFRODITA study: a population-based survey in Spain. J Low Genit Tract Dis. Apr 2008;12(2):82-9. doi:10.1097/LGT.0b013e3181599c16.
39	Ginde AA, Millen JC, Love JS, Pang JM, Camargo CA, Jr. Compliance with recommended cancer screening among emergency department patients: a multicenter survey. Acad Emerg Med. May 2008;15(5):483-6. doi:10.1111/j.1553-2712.2008.00103.x.
40	Tung WC, Nguyen DH, Tran DN. Applying the transtheoretical model to cervical cancer screening in Vietnamese-American women. Int Nurs Rev. Mar 2008;55(1):73-80. doi:10.1111/j.1466-7657.2007.00602.x.
41	Nelson W, Moser RP, Gaffey A, Waldron W. Adherence to cervical cancer screening guidelines for U.S. women aged 25-64: data from the 2005 Health Information National Trends Survey (HINTS). J Womens Health (Larchmt). Nov 2009;18(11):1759-68. doi:10.1089/jwh.2009.1430.
42	Ma GX, Toubbeh JI, Wang MQ, Shive SE, Cooper L, Pham A. Factors associated with cervical cancer screening compliance and noncompliance among Chinese, Korean, Vietnamese, and Cambodian women. J Natl Med Assoc. Jun 2009;101(6):541-51. doi:10.1016/s0027-9684(15)30939-1.
43	Dimitrakaki C, Boulamatsis D, Mariolis A, Kontodimopoulos N, Niakas D, Tountas Y. Use of cancer screening services in Greece and associated social factors: results from the nation-wide Hellas Health I survey. Eur J Cancer Prev. Jun 2009;18(3):248-57. doi:10.1097/CEJ.0b013e32832405d6.
44	Amankwah E, Ngwakongnwi E, Quan H. Why many visible minority women in Canada do not participate in cervical cancer screening. Ethn Health. Aug 2009;14(4):337-49. doi:10.1080/13557850802699122.
45	Taylor VM, Yasui Y, Nguyen TT, et al. Pap smear receipt among Vietnamese immigrants: the importance of health care factors. Ethn Health. Dec 2009;14(6):575-89. doi:10.1080/13557850903111589.

Study ID	Reference
46	YG S, SM Z, XP D, et al. Investigation on cervical cancer screening of women in Yuetan community (月坛社区妇女宫颈癌筛查状况调查). Chinese General Practice (中国全科医学). 2009;12:2092-2093.
47	Martin-Lopez R, Hernandez-Barrera V, De Andres AL, Garrido PC, De Miguel AG, Garcia RJ. Breast and cervical cancer screening in Spain and predictors of adherence. Eur J Cancer Prev. May 2010;19(3):239-45. doi:10.1097/CEJ.0b013e3283372125.
48	Zhou J, Enewold L, Peoples GE, et al. Trends in cancer screening among Hispanic and white non-Hispanic women, 2000-2005. J Womens Health (Larchmt). Dec 2010;19(12):2167-74. doi:10.1089/jwh.2009.1909.
49	Byrne MM, Davila EP, Zhao W, et al. Cancer screening behaviors among smokers and non-smokers. Cancer Epidemiol. Oct 2010;34(5):611-7. doi:10.1016/j.canep.2010.06.017.
50	Lopez-de-Andres A, Martin-Lopez R, Hernandez-Barrera V, et al. Predictors of breast and cervical cancer screening in a Spanish metropolitan area. J Womens Health (Larchmt). Sep 2010;19(9):1675-81. doi:10.1089/jwh.2009.1828.
51	Lim JW. Linguistic and ethnic disparities in breast and cervical cancer screening and health risk behaviors among Latina and Asian American women. J Womens Health (Larchmt). Jun 2010;19(6):1097-107. doi:10.1089/jwh.2009.1614.
52	Paskett ED, McLaughlin JM, Reiter PL, et al. Psychosocial predictors of adherence to risk-appropriate cervical cancer screening guidelines: a cross sectional study of women in Ohio Appalachia participating in the Community Awareness Resources and Education (CARE) project. Prev Med. Jan-Feb 2010;50(1-2):74-80. doi:10.1016/j.ypmed.2009.09.001.
53	Lee K, Lim HT, Hwang SS, Chae DW, Park SM. Socio-economic disparities in behavioural risk factors for cancer and use of cancer screening services in Korean adults aged 30 years and older: the Third Korean National Health and Nutrition Examination Survey, 2005 (KNHANES III). Public Health. Dec 2010;124(12):698-704. doi:10.1016/j.puhe.2010.07.004.
54	RL M, WJ M, YJ X, et al. Screening rates of cervical cancer breast cancer and their influential factors in Guangdong Province (广东省妇女乳腺癌和宫颈癌筛查率及相关影响因素分析). South China Journal of Preventive Medicine (华南预防医学). 2010;36:8-10, 14.
55	CF W, Y Z, K G, et al. Coverage of Pap Smear Screening among Women in Shanghai and Its Influencing Factors (上海地区女性宫颈脱落细胞筛查工作的实施及其影响因素). Journal of Environmental & Occupational Medicine (环境与职业医学). 2010;27(06):337-341.
56	Lawsin C, Erwin D, Bursac Z, Jandorf L. Heterogeneity in breast and cervical cancer screening practices among female Hispanic immigrants in the United States. J Immigr Minor Health. Oct 2011;13(5):834-41. doi:10.1007/s10903-010-9378-9.
57	Cheung NW, Li S, Tang KC. Participation in cardiovascular risk factor and cancer screening among Australian Chinese. Health Promot J Austr. Aug 2011;22(2):147-52. doi:10.1071/he11147.

Study ID	Reference
58	Pons-Vigues M, Puigpinos-Riera R, Rodriguez-Sanz M, Serral G, Palencia L, Borrell C. Preventive control of breast and cervical cancer in immigrant and native women in Spain: the role of country of origin and social class. Int J Health Serv. 2011;41(3):483-99. doi:10.2190/HS.41.3.e.
59	Tekkel M, Veideman T, Rahu M. Use of mammography, Pap test and prostate examination by body mass index during the developmental period of cancer screening in Estonia. Public Health. Oct 2011;125(10):697-703. doi:10.1016/j.puhe.2011.06.013.
60	Nuno T, Castle PE, Harris R, Estrada A, Garcia F, Yuma Project Technical T. Breast and cervical cancer screening utilization among Hispanic women living near the United States-Mexico border. J Womens Health (Larchmt). May 2011;20(5):685-93. doi:10.1089/jwh.2010.2205.
61	Hansen BT, Hukkelberg SS, Haldorsen T, Eriksen T, Skare GB, Nygard M. Factors associated with non-attendance, opportunistic attendance and reminded attendance to cervical screening in an organized screening program: a cross-sectional study of 12,058 Norwegian women. BMC Public Health. Apr 26 2011;11:264. doi:10.1186/1471-2458-11-264.
62	MacLaughlan SD, Lachance JA, Gjelsvik A. Correlation between smoking status and cervical cancer screening: a cross-sectional study. J Low Genit Tract Dis. Apr 2011;15(2):114-9. doi:10.1097/LGT.0b013e3181f58d0d.
63	Vakfari A, Gavana M, Giannakopoulos S, Smyrnakis E, Benos A. Participation rates in cervical cancer screening: experience in rural Northern Greece. Hippokratia. Oct 2011;15(4):346-52.
64	Acikgoz A, Ergor G. Cervical cancer risk levels in Turkey and compliance to the national cervical cancer screening standard. Asian Pac J Cancer Prev. 2011;12(4):923-7.
65	Lee M, Chang HS, Park EC, Yu SH, Sohn M, Lee SG. Factors associated with participation of Korean women in cervical cancer screening examination by age group. Asian Pac J Cancer Prev. 2011;12(6):1457-62.
66	Q L, X Q, L W, et al. Study on determinants of cervical cancer screening service utilization in urban communities in Shanghai,China (上海市社区宫颈癌筛查服务利用影响因素研究). Chinese Health Resources (中国卫生资源). 2011;14(02):111-113.
67	Nguyen-Truong CK, Lee-Lin F, Leo MC, et al. A community-based participatory research approach to understanding pap testing adherence among Vietnamese American immigrants. J Obstet Gynecol Neonatal Nurs. Nov-Dec 2012;41(6):E26-40. doi:10.1111/j.1552-6909.2012.01414.x.
68	Worthington C, McLeish K, Fuller-Thomson E. Adherence over time to cervical cancer screening guidelines: insights from the Canadian National Population Health Survey. J Womens Health (Larchmt). Feb 2012;21(2):199-208. doi:10.1089/jwh.2010.2090.
69	Gonzalez P, Castaneda SF, Mills PJ, Talavera GA, Elder JP, Gallo LC. Determinants of breast, cervical and colorectal cancer screening adherence in Mexican-American women. J Community Health. Apr 2012;37(2):421-33. doi:10.1007/s10900-011-9459-2.
70	Damiani G, Federico B, Basso D, et al. Socioeconomic disparities in the uptake of breast and cervical cancer screening in Italy: a cross sectional study. BMC Public Health. Feb 3 2012;12:99. doi:10.1186/1471-2458-12-99.

Study ID	Reference
71	Martin-Lopez R, Hernandez-Barrera V, de Andres AL, Carrasco-Garrido P, de Miguel AG, Jimenez-Garcia R. Trend in cervical cancer screening in Spain (2003-2009) and predictors of adherence. Eur J Cancer Prev. Jan 2012;21(1):82-8. doi:10.1097/CEJ.0b013e32834a7e46.
72	Muus KJ, Baker-Demaray TB, Bogart TA, et al. Physical activity and cervical cancer testing among American Indian women. J Rural Health. Summer 2012;28(3):320-6. doi:10.1111/j.1748-0361.2011.00394.x.
73	Olesen SC, Butterworth P, Jacomb P, Tait RJ. Personal factors influence use of cervical cancer screening services: epidemiological survey and linked administrative data address the limitations of previous research. BMC Health Serv Res. Feb 14 2012;12:34. doi:10.1186/1472-6963-12-34.
74	Macedo A, Waller J, Patnick J, Marlow L. Cervical screening uptake, political interest and voter turnout: a population-based survey of women in England. J Med Screen. Dec 2012;19(4):189-94. doi:10.1258/jms.2012.012073.
75	Ko KD, Park SM, Lee K. Factors associated with the use of uterine cervical cancer screening services in korean elderly women. Korean J Fam Med. May 2012;33(3):174-81. doi:10.4082/kjfm.2012.33.3.174.
76	Azerkan F, Sparen P, Sandin S, Tillgren P, Faxelid E, Zendehdel K. Cervical screening participation and risk among Swedish-born and immigrant women in Sweden. Int J Cancer. Feb 15 2012;130(4):937-47. doi:10.1002/ijc.26084.
77	Aminisani N, Armstrong BK, Canfell K. Cervical cancer screening in Middle Eastern and Asian migrants to Australia: a record linkage study. Cancer Epidemiol. Dec 2012;36(6):e394-400. doi:10.1016/j.canep.2012.08.009.
78	Nuno T, Gerald JK, Harris R, Martinez ME, Estrada A, Garcia F. Comparison of breast and cervical cancer screening utilization among rural and urban Hispanic and American Indian women in the Southwestern United States. Cancer Causes Control. Aug 2012;23(8):1333-41. doi:10.1007/s10552-012-0012-0.
79	Shelton RC, Jandorf L, King S, Thelemaque L, Erwin DO. Cervical cancer screening among immigrant Hispanics: an analysis by country of origin. J Immigr Minor Health. Aug 2012;14(4):715-20. doi:10.1007/s10903-011-9541-y.
80	Ashok M, Berkowitz Z, Hawkins NA, Tangka F, Saraiya M. Recency of Pap testing and future testing plans among women aged 18-64: analysis of the 2007 Health Information National Trends Survey. J Womens Health (Larchmt). Jul 2012;21(7):705-12. doi:10.1089/jwh.2012.3562.
81	Leone LA, Allicock M, Pignone MP, Johnson LS, Walsh JF, Campbell MK. Cancer screening patterns by weight group and gender for urban African American church members. J Community Health. Apr 2012;37(2):299-306. doi:10.1007/s10900-011-9445-8.
82	Shippee ND, Mullan RJ, Nabhan M, et al. Adherence to preventive recommendations: experience of a cohort presenting for executive health care. Popul Health Manag. Apr 2012;15(2):65-70. doi:10.1089/pop.2011.0029.
83	Aminisani N, Armstrong BK, Canfell K. Participation in cervical screening by older asian and middle eastern migrants in new South wales, australia. Health Promot Perspect. 2012;2(2):274-86. doi:10.5681/hpp.2012.033.
84	Raz R, Shavit O, Stein M, et al. Uptake of Pap smears among women in a large Israeli HMO between 2006 and 2008. Public Health. Jul 2012;126(7):594-9. doi:10.1016/j.puhe.2012.04.005.
85	Borges MF, Dotto LM, Koifman RJ, Cunha Mde A, Muniz PT. Prevalence of uterine cervical cancer testing in Rio Branco, Acre State, Brazil, and factors associated with non-participation in screening. Cad Saude Publica. Jun 2012;28(6):1156-66. doi:10.1590/s0102-311x2012000600014.

Study ID	Reference
86	Almeida CM, Rodriguez MA, Skootsky S, Pregler J, Steers N, Wenger NS. Cervical cancer screening overuse and underuse: patient and physician factors. Am J Manag Care. Jun 2013;19(6):482-9.
87	Morrison TB, Flynn PM, Weaver AL, Wieland ML. Cervical cancer screening adherence among Somali immigrants and refugees to the United States. Health Care Women Int. 2013;34(11):980-8. doi:10.1080/07399332.2013.770002.
88	Oussaid N, Lutringer-Magnin D, Barone G, Haesebaert J, Lasset C. Factors associated with Pap smear screening among French women visiting a general practitioner in the Rhone-Alpes region. Rev Epidemiol Sante Publique. Oct 2013;61(5):437-45. doi:10.1016/j.respe.2013.04.009.
89	Cerigo H, Coutlee F, Franco EL, Brassard P. Factors associated with cervical cancer screening uptake among Inuit women in Nunavik, Quebec, Canada. BMC Public Health. May 3 2013;13:438. doi:10.1186/1471-2458-13-438.
90	Augusto EF, Rosa ML, Cavalcanti SM, Oliveira LH. Barriers to cervical cancer screening in women attending the Family Medical Program in Niteroi, Rio de Janeiro. Arch Gynecol Obstet. Jan 2013;287(1):53-8. doi:10.1007/s00404-012-2511-3.
91	Khadilkar A, Chen Y. Rate of cervical cancer screening associated with immigration status and number of years since immigration in Ontario, Canada. J Immigr Minor Health. Apr 2013;15(2):244-8. doi:10.1007/s10903-012-9711-6.
92	Lee M, Park EC, Chang HS, Kwon JA, Yoo KB, Kim TH. Socioeconomic disparity in cervical cancer screening among Korean women: 1998-2010. BMC Public Health. Jun 6 2013;13:553. doi:10.1186/1471-2458-13-553.
93	Suh M, Choi KS, Lee YY, Park B, Jun JK. Cancer screening in Korea, 2012: results from the Korean National Cancer Screening Survey. Asian Pac J Cancer Prev. 2013;14(11):6459-63. doi:10.7314/apjcp.2013.14.11.6459.
94	Hernandez CM, Wallace D. A profile of Mexican-born women who adhere to national cervical cancer screening recommendations. J Community Health Nurs. 2014;31(3):157-66. doi:10.1080/07370016.2014.926678.
95	Tsai RJ, Luckhaupt SE, Sweeney MH, Calvert GM. Shift work and cancer screening: do females who work alternative shifts undergo recommended cancer screening? Am J Ind Med. Mar 2014;57(3):265-75. doi:10.1002/ajim.22285.
96	Adonis L, Basu D, Luiz J. Predictors of adherence to screening guidelines for chronic diseases of lifestyle, cancers, and HIV in a health-insured population in South Africa. Glob Health Action. 2014;7:23807. doi:10.3402/gha.v7.23807.
97	Moudatsou MM, Kritsotakis G, Alegakis AK, Koutis A, Philalithis AE. Social capital and adherence to cervical and breast cancer screening guidelines: a cross-sectional study in rural Crete. Health Soc Care Community. Jul 2014;22(4):395-404. doi:10.1111/hsc.12096.
98	Kristensson JH, Sander BB, von Euler-Chelpin M, Lynge E. Predictors of non-participation in cervical screening in Denmark. Cancer Epidemiol. Apr 2014;38(2):174-80. doi:10.1016/j.canep.2013.12.007.
99	Arbyn M, Fabri V, Temmerman M, Simoens C. Attendance at cervical cancer screening and use of diagnostic and therapeutic procedures on the uterine cervix assessed from individual health insurance data (Belgium, 2002-2006). PLoS One. 2014;9(4):e92615. doi:10.1371/journal.pone.0092615.
100	Calo WA. Place matters: Associations between contextual characteristics and health. The University of Texas School of Public Health; 2014.

Study ID	Reference
101	Documet P, Bear TM, Flatt JD, Macia L, Trauth J, Ricci EM. The association of social support and education with breast and cervical cancer screening. Health Educ Behav. Feb 2015;42(1):55-64. doi:10.1177/1090198114557124.
102	Guo F, Hirth JM, Berenson AB. Effects of Cardiovascular Disease on Compliance with Cervical and Breast Cancer Screening Recommendations Among Adult Women. J Womens Health (Larchmt). Aug 2015;24(8):641-7. doi:10.1089/jwh.2014.5129.
103	Visanuyothin S, Chompikul J, Mongkolchati A. Determinants of cervical cancer screening adherence in urban areas of Nakhon Ratchasima Province, Thailand. J Infect Public Health. Nov-Dec 2015;8(6):543-52. doi:10.1016/j.jiph.2015.04.018.
104	Ricardo-Rodrigues I, Jimenez-Garcia R, Hernandez-Barrera V, Carrasco-Garrido P, Jimenez-Trujillo I, Lopez de Andres A. Social disparities in access to breast and cervical cancer screening by women living in Spain. Public Health. Jul 2015;129(7):881-8. doi:10.1016/j.puhe.2015.02.021.
105	Miranda-Diaz C, Betancourt E, Ruiz-Candelaria Y, Hunter-Mellado RF. Barriers for Compliance to Breast, Colorectal, and Cervical Screening Cancer Tests among Hispanic Patients. Int J Environ Res Public Health. Dec 22 2015;13(1):ijerph13010021. doi:10.3390/ijerph13010021.
106	Gyulai A, Nagy A, Pataki V, Tonte D, Adany R, Voko Z. Survey of Participation in Organised Cervical Cancer-Screening Programme in Hungary. Cent Eur J Public Health. Dec 2015;23(4):360-4. doi:10.21101/cejph.a4068.
107	Richard A, Rohrmann S, Schmid SM, et al. Lifestyle and health-related predictors of cervical cancer screening attendance in a Swiss population-based study. Cancer Epidemiol. Dec 2015;39(6):870-6. doi:10.1016/j.canep.2015.09.009.
108	Schoofs J, Krijger K, Vandevoorde J, Rossem IV, Devroey D. Health-related factors associated with the participation in cervical cancer screening. J Res Health Sci. Winter 2015;15(1):11-6.
109	Ostensson E, Alder S, Elfstrom KM, et al. Barriers to and facilitators of compliance with clinic-based cervical cancer screening: population-based cohort study of women aged 23-60 years. PLoS One. 2015;10(5):e0128270. doi:10.1371/journal.pone.0128270.
110	Barbadoro P, Ricciardi A, Di Tondo E, Vallorani S, Mazzarini G, Prospero E. Utilization patterns of cervical cancer screening in Italy. Eur J Cancer Prev. Mar 2015;24(2):135-40. doi:10.1097/CEJ.0000000000000068.
111	MX D, LH R, HJ W, et al. Cervical cancer screening service utilization and its determinants among rural women. (农村适龄妇女宫颈癌筛查服务利用行为现状及其影响因素分析). Modern Preventive Medicine (现代预防医学). 2015;42:1604-1606.
112	Hirth JM, Laz TH, Rahman M, Berenson AB. Racial/Ethnic Differences Affecting Adherence to Cancer Screening Guidelines Among Women. J Womens Health (Larchmt). Apr 2016;25(4):371-80. doi:10.1089/jwh.2015.5270.
113	Crawford A, Benard V, King J, Thomas CC. Understanding Barriers to Cervical Cancer Screening in Women With Access to Care, Behavioral Risk Factor Surveillance System, 2014. Prev Chronic Dis. Nov 10 2016;13:E154. doi:10.5888/pcd13.160225.

Study ID	Reference
114	Shneyderman Y, Rutten LJ, Arheart KL, Byrne MM, Kornfeld J, Schwartz SJ. Health Information Seeking and Cancer Screening Adherence Rates. J Cancer Educ. Mar 2016;31(1):75-83. doi:10.1007/s13187-015-0791-6.
115	Valdovinos C, Penedo FJ, Isasi CR, et al. Perceived discrimination and cancer screening behaviors in US Hispanics: the Hispanic Community Health Study/Study of Latinos Sociocultural Ancillary Study. Cancer Causes Control. Jan 2016;27(1):27-37. doi:10.1007/s10552-015-0679-0.
116	Urrutia MT, Gajardo M. Factors affecting compliance with pap smear screening. Rev Med Chil. Dec 2016;144(12):1553-1560. doi:10.4067/S0034-98872016001200006.
117	Bianco A, Larosa E, Pileggi C, Nobile CGA, Pavia M. Cervical and breast cancer screening participation and utilisation of maternal health services: a cross-sectional study among immigrant women in Southern Italy. BMJ Open. Oct 15 2017;7(10):e016306. doi:10.1136/bmjopen-2017-016306.
118	Comparetto C, Epifani C, Manca MC, et al. Uptake of cervical cancer screening among the migrant population of Prato Province, Italy. Int J Gynaecol Obstet. Mar 2017;136(3):309-314. doi:10.1002/ijgo.12067.
119	Parekh N, Donohue JM, Men A, Corbelli J, Jarlenski M. Cervical Cancer Screening Guideline Adherence Before and After Guideline Changes in Pennsylvania Medicaid. Obstet Gynecol. Jan 2017;129(1):66-75. doi:10.1097/AOG.0000000000001804.
120	Leinonen MK, Campbell S, Klungsoyr O, Lonnberg S, Hansen BT, Nygard M. Personal and provider level factors influence participation to cervical cancer screening: A retrospective register-based study of 1.3 million women in Norway. Prev Med. Jan 2017;94:31-39. doi:10.1016/j.ypmed.2016.11.018.
121	White A, Thompson TD, White MC, et al. Cancer Screening Test Use - United States, 2015. MMWR Morb Mortal Wkly Rep. Mar 3 2017;66(8):201-206. doi:10.15585/mmwr.mm6608a1.
122	Tung WC, Granner M, Lu M, Qiu X. Predictors of cervical cancer screening for Chinese American women. Eur J Cancer Care (Engl). Jul 2017;26(4)doi:10.1111/ecc.12552.
123	Cadet TJ, Burke SL, Stewart K, Howard T, Schonberg M. Cultural and emotional determinants of cervical cancer screening among older Hispanic women. Health Care Women Int. Dec 2017;38(12):1289-1312. doi:10.1080/07399332.2017.1364740.
124	Lofters AK, Schuler A, Slater M, et al. Using self-reported data on the social determinants of health in primary care to identify cancer screening disparities: opportunities and challenges. BMC Fam Pract. Feb 28 2017;18(1):31. doi:10.1186/s12875-017-0599-z.
125	Zorogastua K, Sriphanlop P, Reich A, Aly S, Cisse A, Jandorf L. Breast and Cervical Cancer Screening among US and non US Born African American Muslim Women in New York City. AIMS Public Health. 2017;4(1):78-93. doi:10.3934/publichealth.2017.1.78.
126	Chang HK, Myong JP, Byun SW, et al. Factors associated with participation in cervical cancer screening among young Koreans: a nationwide cross-sectional study. BMJ Open. Apr 3 2017;7(4):e013868. doi:10.1136/bmjopen-2016-013868.
127	Cofie LE, Hirth JM, Wong R. Chronic comorbidities and cervical cancer screening and adherence among US-born and foreign-born women. Cancer Causes Control. Nov 2018;29(11):1105-1113. doi:10.1007/s10552-018-1084-2.

Study ID	Reference
128	Luque JS, Tarasenko YN, Chen C. Correlates of Cervical Cancer Screening Adherence Among Women in the U.S.: Findings from HINTS 2013-2014. J Prim Prev. Aug 2018;39(4):329-344. doi:10.1007/s10935-018-0513-z.
129	Rendle KA, Schiffman M, Cheung LC, et al. Adherence patterns to extended cervical screening intervals in women undergoing human papillomavirus (HPV) and cytology cotesting. Prev Med. Apr 2018;109:44-50. doi:10.1016/j.ypmed.2017.12.023.
130	Petrelli A, Giorgi Rossi P, Francovich L, et al. Geographical and socioeconomic differences in uptake of Pap test and mammography in Italy: results from the National Health Interview Survey. BMJ Open. Sep 19 2018;8(9):e021653. doi:10.1136/bmjopen-2018-021653.
131	Han J, Jungsuwadee P, Abraham O, Ko D. Shared Decision-Making and Women's Adherence to Breast and Cervical Cancer Screenings. Int J Environ Res Public Health. Jul 17 2018;15(7). doi:10.3390/ijerph15071509.
132	Restivo V, Costantino C, Marras A, et al. Pap Testing in a High-Income Country with Suboptimal Compliance Levels: A Survey on Acceptance Factors among Sicilian Women. Int J Environ Res Public Health. Aug 22 2018;15(9). doi:10.3390/ijerph15091804.
133	Luque JS, Tarasenko YN, Li H, Davila CB, Knight RN, Alcantar RE. Utilization of Cervical Cancer Screening Among Hispanic Immigrant Women in Coastal South Carolina. J Racial Ethn Health Disparities. Jun 2018;5(3):588-597. doi:10.1007/s40615-017-0404-7.
134	Petkeviciene J, Ivanauskiene R, Klumbiene J. Sociodemographic and lifestyle determinants of non-attendance for cervical cancer screening in Lithuania, 2006-2014. Public Health. Mar 2018;156:79-86. doi:10.1016/j.puhe.2017.12.014.
135	Moreno PI, Yanez B, Schuetz SJ, et al. Cancer fatalism and adherence to national cancer screening guidelines: Results from the Hispanic Community Health Study/Study of Latinos (HCHS/SOL). Cancer Epidemiol. Jun 2019;60:39-45. doi:10.1016/j.canep.2019.03.003.
136	Lucas-Wright A, Duran P, Bazargan M, Vargas C, Maxwell AE. Cancer-related Knowledge, Attitudes and Behaviors within the Latino Faith Community in South Los Angeles. Ethn Dis. Spring 2019;29(2):239-246. doi:10.18865/ed.29.2.239.
137	MacLaughlin KL, Jacobson RM, Radecki Breitkopf C, et al. Trends Over Time in Pap and Pap-HPV Cotesting for Cervical Cancer Screening. J Womens Health (Larchmt). Feb 2019;28(2):244-249. doi:10.1089/jwh.2018.7380.
138	Kaso M, Takahashi Y, Nakayama T. Factors related to cervical cancer screening among women of childrearing age: a cross-sectional study of a nationally representative sample in Japan. Int J Clin Oncol. Mar 2019;24(3):313-322. doi:10.1007/s10147-018-1350-z.
139	Perez CM, Gonzalez-Barrios D, Ramos-Cartagena JM, Ortiz AP. Association of Body Mass Index With Adherence to Cervical Cancer Screening Recommendations in Hispanic Women. J Low Genit Tract Dis. Jan 2020;24(1):14-20. doi:10.1097/LGT.0000000000000504.
140	Charkhchi P, Schabath MB, Carlos RC. Breast, Cervical, and Colorectal Cancer Screening Adherence: Effect of Low Body Mass Index in Women. J Womens Health (Larchmt). Jul 2020;29(7):996-1006. doi:10.1089/jwh.2019.7739.

Study ID	Reference
141	Abar B, Dalawari P, Ogedegbe C, Santoro-Terray L, Adler D, Bradley K. Identifying Cancer Screening Adherence in the Emergency Department Utilizing Research Associates. J Emerg Med. Dec 2020;59(6):894-899. doi:10.1016/j.jemermed.2020.07.013.
142	Maxwell AE, Lucas-Wright A, Gatson J, et al. Community health advisors assessing adherence to national cancer screening guidelines among African Americans in South Los Angeles. Prev Med Rep. Jun 2020;18:101096. doi:10.1016/j.pmedr.2020.101096.
143	Zamorano-Leon JJ, Lopez-de-Andres A, Alvarez-Gonzalez A, et al. Reduction from 2011 to 2017 in adherence to breast cancer screening and non-improvement in the uptake of cervical cancer screening among women living in Spain. Maturitas. May 2020;135:27-33. doi:10.1016/j.maturitas.2020.02.007.
144	Garrido CO, Coskun RA, Lent AB, Calhoun E, Harris RB. Use of cervical cancer preventive services among US women aged 21-29: an assessment of the 2010 Affordable Care Act rollout through 2018. Cancer Causes Control. Sep 2020;31(9):839-850. doi:10.1007/s10552-020-01325-w.
145	Wilding S, Wighton S, Halligan D, West R, Conner M, O'Connor DB. What factors are most influential in increasing cervical cancer screening attendance? An online study of UK-based women. Health Psychol Behav. Jan 1 2020;8(1):314-328. doi:10.1080/21642850.2020.1798239.
146	Franck JE, Ringa V, Rigal L, et al. Patterns of gynaecological check-up and their association with body mass index within the CONSTANCES cohort. J Med Screen. Mar 2020;28(1):10-17. doi:10.1177/0969141320914323.
147	Lee HY, Choi YJ, Shin J, Yoon YJ, An S. Adherence to Cervical Cancer Screening in Korean American Immigrant Women: Identifying Malleable Variables for Intervention Development. J Transcult Nurs. May 2020;32(3):230-238. doi:10.1177/1043659620914693.
148	Barrera-Castillo M, Fernandez-Pena R, Del Valle-Gomez MDO, Fernandez-Feito A, Lana A. Social integration and gynecologic cancer screening of immigrant women in Spain. Gac Sanit. Sep - Oct 2020;34(5):468-473. doi:10.1016/j.gaceta.2019.01.002.
149	Deguara M, Calleja N, England K. Cervical cancer and screening: knowledge, awareness and attitudes of women in Malta. J Prev Med Hyg. Dec 2020;61(4):E584-E592. doi:10.15167/2421-4248/jpmh2020.61.4.1521.
150	dos Santos NLF, Valadares LR, de Melo AT, et al. Receptivity and Factors Associated with Cervical Cancer Preventive Measures in a Capital City of Northern Brazil. Mundo Saude. Jan-Dec 2020;44(1):454-464. doi:10.15343/0104-7809.202044454464.
151	Malone C, Buist DSM, Tiro J, et al. Out of reach? Correlates of cervical cancer underscreening in women with varying levels of healthcare interactions in a United States integrated delivery system. Prev Med. Apr 2021;145:106410. doi:10.1016/j.ypmed.2020.106410.
152	Pelullo CP, Cantore F, Lisciotto A, Di Giuseppe G, Pavia M. Organized Breast and Cervical Cancer Screening: Attendance and Determinants in Southern Italy. Cancers (Basel). Mar 30 2021;13(7). doi:10.3390/cancers13071578.
153	Ishii K, Tabuchi T, Iso H. Combined patterns of participation in cervical, breast, and colorectal cancer screenings and factors for non-participation in each screening among women in Japan. Prev Med. May 18 2021:106627. doi:10.1016/j.ypmed.2021.106627.

Table S6 Basic characteristics of the included studies

Study ID	Author	Year published/ Year investigated	Country	WHO/WB region	Place	SDI	Data source [a]	Study design [b]	Definition of adherence	Screening method	Screening interval	Sample size [c]	Adherent women	Age range (year)
**1**	Cohen MM, et al.	1992/1983	Canada	AMR/HIC	Both	0.771	2	1	Pap test every 5 years	Pap	5 years	22287	12414	27-66
**2**	Lantz PM, et al.	1997/1993	USA	AMR/HIC	Rural	0.778	1	1	Pap test in 3 years	Pap	3 years	1168	816	≥40
**3**	Risendal B, et al.	1999/1996	USA	AMR/HIC	Urban	0.788	1	1	Pap test every year	Pap	1 year	519	257	≥18
**4**	Fernandez ME, et al.	1999/1996	USA	AMR/HIC	NA	0.788	1	1	Pap test every year	Pap	1 year	15427	5627	≥18
**5**	Rohlfs I, et al.	1999/1992	Spain	EUR/HIC	NA	0.662	1	1	Pap test every 3 or 5 years	Pap	3/5 years	1667	776	≥30
**6**	Taylor VM, et al.	1999/1998	USA	AMR/HIC	NA	0.791	1	1	Pap test every year	Pap	1 year	406	190	≥18
**7**	Maxwell AE, et al.	2000/1996	USA	AMR/HIC	NA	0.788	1	1	Pap test every year or every 2 years	Pap	1/2 years	447	199	50-83
**8**	Ramirez AG, et al.	2000/1994	USA	AMR/HIC	NA	0.782	1	1	Pap test every 3 years	Pap	3 years	3928	3081	≥18
**9**	Maxwell CJ, et al.	2001/1997	Canada	AMR/HIC	NA	0.812	1	1	Pap test every 3 years	Pap	3 years	33817	24348	≥18
**10**	Do HH, et al.	2001/1999	USA	AMR/HIC	Urban	0.794	1	1	Pap test every 2 years	Pap	2 years	647	387	≥20
**11**	Jirojwong S, et al.	2001/1998	Australia	WPR/HIC	NA	0.770	1	1	Biennial cervical screening every 5 years	Pap	5 years (twice)	134	52	NA

Study ID	Author	Year published/ Year investigated	Country	WHO/WB region	Place	SDI	Data source [a]	Study design [b]	Definition of adherence	Screening method	Screening interval	Sample size [c]	Adherent women	Age range (year)
**12**	Lee J, et al.	2002/1998	Singapore	WPR/HIC	NA	0.756	1	1	Pap test every 3 years	Pap	3 years	726	302	30-59
**13**	Cyrus-David MS, et al.	2002/1996	USA	AMR/HIC	Rural	0.788	1	1	At least 3 Pap tests for high-risk women and 1 Pap test for low-risk women every 3 years	Pap	1 year (high-risk women)/3 years (low-risk women)	614	254	≥40
**14**	Amonkar MM, et al.	2002/1997	USA	AMR/HIC	Both	0.789	1	1	Pap test every 3 years	Pap	3 years	23498	20366	≥18
**15**	Fernandez-Esquer ME, et al.	2003/1992	USA	AMR/HIC	Urban	0.775	1	1	Biennial Pap test every 5 years	Pap	5 years (twice)	1804	1020	18-91
**16**	Selvin E, et al.	2003/1998	USA	AMR/HIC	NA	0.791	1	1	Pap test every 3 years	Pap	3 years	5509	4572	40-64
**17**	Juon HS, et al.	2003/2000	USA	AMR/HIC	NA	0.797	1	1	Pap test every 2 years	Pap	2 years	459	177	≥40
**18**	Miedema BB, et al.	2003/2002	Canada	AMR/HIC	Rural	0.832	1	1	Pap test every 3 years	Pap	3 years	518	471	50-69
**19**	Hou SI, et al.	2003/1999	China	WPR/HIC	NA	0.747	1	1	Pap test every 3 years	Pap	3 years	125	73	NA
**20**	Luengo Matos S, et al.	2003/2000	Spain	EUR/HIC	NA	0.705	1	1	Pap test every 5 years	Pap	5 years	2409	1196	40-70

Study ID	Author	Year published/ Year investigated	Country	WHO/WB region	Place	SDI	Data source [a]	Study design [b]	Definition of adherence	Screening method	Screening interval	Sample size [c]	Adherent women	Age range (year)
**21**	Taylor VM, et al.	2004/2002	USA	AMR/HIC	NA	0.806	1	1	Pap test every 3 years	Pap	3 years	352	240	18-64
**22**	Coronado GD, et al.	2004/1998	USA	AMR/HIC	Rural	0.791	1	1	Pap test every 3 years	Pap	3 years	767	612	18-64
**23**	Insinga RP, et al.	2004/2000	USA	AMR/HIC	NA	0.797	2	1	Pap test every 3 years	Pap	3 years	28692	19238	≥15
**24**	Dettenborn L, et al.	2004/1999	USA	AMR/HIC	Urban	0.794	1	1	Pap test every 1 year	Pap	1 year	181	119	≥20
**25**	Borrayo EA, et al.	2004/1999	USA	AMR/HIC	NA	0.794	1	1	Pap test every 1 year	Pap	1 year	153	100	22-84
**26**	Bazargan M, et al.	2004/1999	USA	AMR/HIC	Urban	0.794	1	1	Pap test every 2 years	Pap	2 years	230	144	18-88
**27**	Cho IS, et al.	2004/2000	South Korea	WPR/HIC	Urban	0.784	1	1	Pap test every 2 years	Pap	2 years	200	109	40-60
**28**	Hoyo C, et al.	2005/2001	USA	AMR/HIC	NA	0.802	1	1	Pap test every 3 years	Pap	3 years	144	119	45-65
**29**	De Alba I， et al.	2005/2001	USA	AMR/HIC	NA	0.802	1	1	Pap test every 3 years	Pap	3 years	25228	25027	≥18
**30**	Kwak MS, et al.	2005/2004	South Korea	WPR/HIC	NA	0.811	1	1	Pap test every 2 years	Pap	2 years	1545	901	≥30
**31**	Giorgi Rossi P, et al.	2006/2004	Italy	EUR/HIC	Rural	0.766	2	1	Pap test every 3 years	Pap	3 years	641	554	25-64
**32**	Carruth AK, et al.	2006/2001	USA	AMR/HIC	Rural	0.802	1	1	Pap test every 3 years	Pap	3 years	2324	1742	>18
**33**	Liao CC, et al.	2006/2000	China	WPR/HIC	Both	0.754	1	1	Pap test every 1 year	Pap	1 year	1021	240	≥20

Study ID	Author	Year published/ Year investigated	Country	WHO/WB region	Place	SDI	Data source [a]	Study design [b]	Definition of adherence	Screening method	Screening interval	Sample size [c]	Adherent women	Age range (year)
**34**	Lee-Lin F, et al.	2007/2002	USA	AMR/HIC	Urban	0.806	1	1	Pap test every 3 years	Pap	3 years	100	68	40-91
**35**	Chen LS, et al.	2007/2002	China	WPR/HIC	Both	0.772	2	1	Pap test every 3 years	Pap	3 years	5815781	3034631	≥30
**36**	Gao W, et al.	2008/2007	New Zealand	WPR/HIC	NA	0.800	1	1	Pap test every 3 years	Pap	3 years	234	131	20-69
**37**	Tsui J, et al.	2008/2003	USA	AMR/HIC	NA	0.809	1	1	Pap test every 3 years	Pap	3 years	322	198	≥18
**38**	Puig-Tintore LM, et al.	2008/2005	Spain	EUR/HIC	Both	0.725	1	1	Pap test every 3 years	Pap	3 years	5789	4376	18-65
**39**	Ginde AA, et al.	2008/2007	USA	AMR/HIC	Urban	0.814	1	1	Pap test every 3 years	Pap	3 years	202	135	≥18
**40**	Tung WC, et al.	2008/2003	USA	AMR/HIC	NA	0.809	1	1	Pap test every 1 year	Pap	1 year	80	37	20-65
**41**	Nelson W, et al.	2009/2005	USA	AMR/HIC	NA	0.812	1	1	Cervical screening every 3 years	Pap	3 years	2070	1739	25-64
**42**	Ma GX, et al.	2009/2006	USA	AMR/HIC	NA	0.811	1	1	Pap test every 1 year	Pap	1 year	1049	404	18-65
**43**	Dimitrakaki C, et al.	2009/2006	Greece	EUR/HIC	Both	0.765	1	1	Pap test every 3 years	Pap	3 years	424	252	21-69
**44**	Amankwah E, et al.	2009/2003	Canada	AMR/HIC	NA	0.835	1	1	Pap test every 3 years	Pap	3 years	76214	65442	18-65
**45**	Taylor VM, et al.	2009/2005	USA	AMR/HIC	NA	0.812	1	1	Pap test every 3 years	Pap	3 years	1516	1228	20-79

Study ID	Author	Year published/ Year investigated	Country	WHO/WB region	Place	SDI	Data source [a]	Study design [b]	Definition of adherence	Screening method	Screening interval	Sample size [c]	Adherent women	Age range (year)
**46**	Sun YG, et al.	2009/2004	China	WPR/LMIC	Urban	0.561	1	1	Cervical screening every 2 years	NA	2 years	1490	790	25-65
**47**	Martin-Lopez R, et al.	2010/2007	Spain	EUR/HIC	NA	0.730	1	1	Pap test every 3 years	Pap	3 years	9725	6555	25-64
**48A**	Zhou J, et al.	2010/2000	USA	AMR/HIC	NA	0.797	1	1	Pap test every 1 year for women aged 18-29 years; every 3 years for women aged 30-69 years	Pap	1 year (18-29 years old)/3 years (30-69 years old)	10773	8565	18-69
**48B**	Zhou J, et al.	2010/2005	USA	AMR/HIC	NA	0.812	1	1	Pap test every 1 year for women aged 18-29 years; every 3 years for women aged 30-69 years	Pap	1 year (18-29 years old)/3 years (30-69 years old)	10392	8037	18-69
**49**	Byrne MM, et al.	2010/2007	USA	AMR/HIC	NA	0.814	1	1	Pap test every 3 years	Pap	3 years	21081	16371	≥21
**50**	Lopez-de-Andres A, et al.	2010/2005	Spain	EUR/HIC	Urban	0.725	1	1	Pap test every 3 years	Pap	3 years	3200	2451	18-69
**51**	Lim JW	2010/2007	USA	AMR/HIC	NA	0.814	1	1	Pap test every 3 years	Pap	3 years	6051	4885	≥18
**52**	Paskett ED, et al.	2010/2006	USA	AMR/HIC	NA	0.811	2	1	Pap test in 13 months for high-risk women; Pap test in 37 months for low-risk women	Pap	13 months (high-risk women)/37	562	380	≥18

Study ID	Author	Year published/ Year investigated	Country	WHO/WB region	Place	SDI	Data source [a]	Study design [b]	Definition of adherence	Screening method	Screening interval	Sample size [c]	Adherent women	Age range (year)
											months (low-risk women)			
**53**	Lee K, et al.	2010/2005	South Korea	WPR/HIC	Both	0.817	1	1	Pap test every 2 years	Pap	2 years	3413	1457	≥30
**54**	Meng RL, et al.	2010/2005	China	WPR/LMIC	Both	0.571	1	1	Pap test every 3 years	Pap	3 years	3457	415	15-68
**55**	Wang CF, et al.	2010/2007	China	WPR/LMIC	Both	0.591	1	1	Pap test every 3 years	Pap	3 years	10520	3407	15-69
**56**	Lawsin C, et al.	2011/2006	USA	AMR/HIC	NA	0.811	1	1	Pap test every 3 years	Pap	3 years	236	190	NA
**57**	Cheung NW, et al.	2011/2004	Australia	WPR/HIC	NA	0.795	1	1	Pap test every 2 years	Pap	2 years	249	206	18-70
**58**	Pons-Vigues M, et al.	2011/2006	Spain	EUR/HIC	NA	0.730	1	1	Pap test every 5 years	Pap	5 years	10093	6250	25-65
**59**	Tekkel M, et al.	2011/2004	Estonia	EUR/HIC	Both	0.759	1	1	Pap test every 2 years	Pap	2 years	3226	1205	25-64
**60**	Nuno T, et al.	2011/2006	USA	AMR/HIC	Both	0.811	1	2/1	Pap test every 3 years	Pap	3 years	289	212	50-
**61**	Hansen BT, et al.	2011/2005	Norway	EUR/HIC	NA	0.874	2	1	Pap test every 4 years	Pap	4 years	12058	10444	18-45

Study ID	Author	Year published/ Year investigated	Country	WHO/WB region	Place	SDI	Data source [a]	Study design [b]	Definition of adherence	Screening method	Screening interval	Sample size [c]	Adherent women	Age range (year)
**62**	MacLaughlan SD, et al.	2011/2006	USA	AMR/HIC	NA	0.811	1	1	Pap test every 3 years	Pap	3 years	150786	126509	≥18
**63**	Vakfari A, et al.	2011/2007	Greece	EUR/HIC	Rural	0.768	1	1	Pap test every 3 years	Pap	3 years	214	92	20-64
**64**	Acikgoz A, et al.	2011/2009	Turkey	EUR/LMIC	NA	0.671	1	1	Pap test every 5 years	Pap	5 years	227	89	35-69
**65**	Lee M, et al.	2011/2008	South Korea	WPR/HIC	Both	0.833	1	1	Pap test every 2 years	Pap	2 years	6964	3448	≥30
**66**	Liang Q, et al.	2011/2006	China	WPR/LMIC	Urban	0.581	1	1	Cervical screening every 3 years	NA	3 years	4703	1195	30-65
**67**	Nguyen-Truong, CK, et al.	2012/2010	USA	AMR/HIC	NA	0.832	1	1	Pap test every 3 years	Pap	3 years	211	145	21-87
**68**	Worthington C, et al.	2012/2000	Canada	AMR/HIC	Both	0.824	1	1	Pap test every 3 years	Pap	3 years	4949	3039	20-70
**69**	Gonzalez P, et al.	2012/2007	USA	AMR/HIC	NA	0.814	1	1	Pap test every 3 years	Pap	3 years	319	274	40-56
**70**	Damiani G, et al.	2012/2005	Italy	EUR/HIC	NA	0.768	1	1	Pap test every 3 years	Pap	3 years	35349	18417	25-64

Study ID	Author	Year published/ Year investigated	Country	WHO/WB region	Place	SDI	Data source [a]	Study design [b]	Definition of adherence	Screening method	Screening interval	Sample size [c]	Adherent women	Age range (year)
**71**	Martin-Lopez R, et al.	2012/2009	Spain	EUR/HIC	NA	0.739	1	1	Pap test every 3 years	Pap	3 years	7634	5048	25-64
**72**	Muus KJ, et al.	2012/2005	USA	AMR/HIC	Both	0.812	1	1	Pap test every 3 years	Pap	3 years	1979	1187	≥18
**73**	Olesen SC, et al.	2012/2007	Australia	WPR/HIC	NA	0.799	1	1	Pap test every 2 years	Pap	2 years	1685	1019	44-48/64-68
**74**	Macedo A, et al.	2012/2010	UK	EUR/HIC	NA	0.820	1	1	Pap test every 3 years for women aged 26-49 years; every 5 years for women aged 50–64 years	Pap	3 years (26-49 years old)/5 years (30-65 years old)	890	717	26-64
**75**	Ko KD, et al.	2012/2005	South Korea	WPR/HIC	NA	0.817	1	1	Pap test every 2 years	Pap	2 years	654	111	≥65
**76**	Azerkan F, et al.	2012/1999	Sweden	EUR/HIC	NA	0.819	2	1	Pap test every 3 years for women aged 23-50 years; every 5 years for women aged 51-60 years	Pap	3 years (23-50 years old)/5 years (51-60 years old)	2621802	1567596	23-60
**77**	Aminisani N, et al.	2012/2002	Australia	WPR/HIC	NA	0.787	2	1	Pap tests every 2 years	Pap	2 years	24506	10494	40-64
**78**	Nuno T, et al.	2012/2008	USA	AMR/HIC	Both	0.819	1	1	Pap test every 3 years	Pap	3 years	10487	8787	40-

Study ID	Author	Year published/ Year investigated	Country	WHO/WB region	Place	SDI	Data source [a]	Study design [b]	Definition of adherence	Screening method	Screening interval	Sample size [c]	Adherent women	Age range (year)
**79**	Shelton RC, et al.	2012/2007	USA	AMR/HIC	NA	0.814	1	1	Pap test every 3 years	Pap	3 years	1305	968	18-
**80**	Ashok M, et al.	2012/2008	USA	AMR/HIC	NA	0.819	1	1	Pap test every 2 or 3 years	Pap	2/3 years	2915	2387	18-64
**81**	Leone LA, et al.	2012/2007	USA	AMR/HIC	Urban	0.814	1	2/1	Pap test every 2 years	Pap	2 years	668	503	≥50
**82**	Shippee ND, et al.	2012/2007	USA	AMR/HIC	NA	0.814	1	1	Pap test every 3 years	Pap	3 years	1893	1731	21-65
**83**	Aminisani N, et al.	2012/2003	Australia	WPR/HIC	NA	0.791	2	2	Pap test every 2 to 3 years	Pap	2-3 years	22239	13713	20-54
**84**	Raz R, et al.	2012/2007	Israel	EUR/HIC	NA	0.778	2	1	Pap test every 3 years	Pap	3 years	489663	225795	21-59
**85**	Borges MF, et al.	2012/2008	Brazil	AMR/LMIC	Urban	0.577	1	1	Pap test every 3 years	Pap	3 years	772	586	18-69
**86**	Almeida CM, et al.	2013/2008	USA	AMR/HIC	NA	0.819	2	1	Pap test every 3 years	Pap	3 years	8018	2686	30-91
**87**	Morrison TB, et al.	2013/2007	USA	AMR/HIC	NA	0.814	2	1	Pap test every 3 years	Pap	3 years	310	158	18-65
**88**	Oussaid N, et al.	2013/2008	France	EUR/HIC	Both	0.801	1	1	Pap test every 3 years	Pap	3 years	1186	1057	≥25
**89**	Cerigo H, et al.	2013/2005	Canada	AMR/HIC	NA	0.840	1	1	Pap test every 3 years	Pap	3 years	403	307	21-69

Study ID	Author	Year published/ Year investigated	Country	WHO/WB region	Place	SDI	Data source [a]	Study design [b]	Definition of adherence	Screening method	Screening interval	Sample size [c]	Adherent women	Age range (year)
**90**	Augusto EF, et al.	2013/2010	Brazil	AMR/LMIC	Urban	0.590	1	1	Pap test every 3 years	Pap	3 years	351	282	17-79
**91**	Khadilkar A, et al.	2013/2008	Canada	AMR/HIC	NA	0.845	1	1	Pap test every 3 years	Pap	3 years	16706	12429	20-69
**92A**	Lee M, et al.	2013/1998	South Korea	WPR/HIC	NA	0.767	1	1	Pap test every 2 years	Pap	2 years	2725	1065	≥30
**92B**	Lee M, et al.	2013/2001	South Korea	WPR/HIC	NA	0.791	1	1	Pap test every 2 years	Pap	2 years	1622	646	≥30
**92C**	Lee M, et al.	2013/2005	South Korea	WPR/HIC	NA	0.817	1	1	Pap test every 2 years	Pap	2 years	2596	1166	≥30
**92D**	Lee M, et al.	2013/2008	South Korea	WPR/HIC	NA	0.833	1	1	Pap test every 2 years	Pap	2 years	2944	1362	≥30
**92E**	Lee M, et al.	2013/2010	South Korea	WPR/HIC	NA	0.842	1	1	Pap test every 2 years	Pap	2 years	2737	1408	≥30
**93**	Suh M, et al.	2013/2012	South Korea	WPR/HIC	Both	0.851	1	1	Pap test every 2 years	Pap	2 years	2395	1626	≥30
**94**	Hernandez CM, et al	2014/2011	USA	AMR/HIC	NA	0.835	1	1	Biennial Pap tests every 5 years	Pap	5 years (twice)	95	86	20-71
**95**	Tsai RJ, et al.	2014/2010	USA	AMR/HIC	NA	0.832	1	1	Pap test every 3 years	Pap	3 years	6238	5264	21-65
**96**	Adonis L, et al.	2014/2009	South Africa	AFR/LMIC	NA	0.637	1	1	Pap test every 3 years	Pap	3 years	2529	296	≥16

Study ID	Author	Year published/ Year investigated	Country	WHO/WB region	Place	SDI	Data source [a]	Study design [b]	Definition of adherence	Screening method	Screening interval	Sample size [c]	Adherent women	Age range (year)
**97**	Moudatsou MM, et al.	2014/2007	Greece	EUR/HIC	Rural	0.768	1	1	Pap test every 3 years	Pap	3 years	125	34	35-75
**98**	Kristensson JH, et al.	2014/2005	Denmark	EUR/HIC	NA	0.860	2	1	Pap test every 5 years	Pap	5 years	1052447	868957	25-54
**99A**	Arbyn M, et al.	2014/2005	Belgium	EUR/HIC	Both	0.805	2	1	Pap test every 3 years	Pap	3 years	2785516	1706043	25-64
**99B**	Arbyn M, et al.	2014/2003	Belgium	EUR/HIC	Both	0.799	2	1	Pap test every 3 years	Pap	3 years	2755113	1671840	25-64
**99C**	Arbyn M, et al.	2014/1999	Belgium	EUR/HIC	Both	0.784	2	1	Pap test every 3 years	Pap	3 years	2716933	1591255	25-64
**99D**	Arbyn M, et al.	2014/1997	Belgium	EUR/HIC	Both	0.779	2	1	Pap test every 3 years	Pap	3 years	2703047	1511864	25-64
**100**	Calo WA	2014/2010	USA	AMR/HIC	NA	0.832	1	1	Pap test every 3 years	Pap	3 years	2124	1708	21-65
**101**	Documet P, et al.	2015/2010	USA	AMR/HIC	NA	0.832	1	1	Pap test every year for women aged 21-29 years; every 3 years for women aged 30-65 years	Pap	1 year (21-29 years old)/3 years (30-65 years old)	2123	1753	21-65
**102**	Guo F, et al.	2015/2013	USA	AMR/HIC	Both	0.842	1	1	Pap test every 3 years	Pap	3 years	11788	9654	21-65
**103**	Visanuyothin S, et al.	2015/2012	Thailand	SEAR/LMIC	Urban	0.649	1	1	Pap test every 5 years	Pap	5 years	595	389	30-60
**104**	Ricardo-Rodrigues I, et al.	20152012	Spain	EUR/HIC	NA	0.749	1	1	Pap test every 5 years	Pap	3 years	7022	4929	25-65

Study ID	Author	Year published/ Year investigated	Country	WHO/WB region	Place	SDI	Data source [a]	Study design [b]	Definition of adherence	Screening method	Screening interval	Sample size [c]	Adherent women	Age range (year)
**105**	Miranda-Diaz C, et al.	20152012	USA	AMR/HIC	Urban	0.839	1	1	Pap test every year	Pap	1 year	122	90	21-85
**106**	Gyulai A, et al.	2015/2008	Hungary	EUR/HIC	NA	0.763	1	1	Cervical screening every 3 years	Complex screenings and Pap	3 years	3306	2446	25-65
**107**	Richard A, et al.	2015/2012	Switzerland	EUR/HIC	NA	0.917	1	1	Cervical screening every 3 years	NA	3 years	7319	5336	20-69
**108**	Schoofs J, et al.	2015/2012	Belgium	EUR/HIC	NA	0.829	1	1	Pap test every 3 years	Pap	3 years	408	269	25-64
**109**	Oestensson E, et al.	2015/2013	Sweden	EUR/HIC	Urban	0.858	1	1	Cervical screening every 3 years for women aged 23-50 years; every 5 years for women aged 51-60 years	NA	3 years (21-50 years old)/5 years (51-60 years old)	1510	763	23-60
**110**	Barbadoro P, et al.	2015/2005	Italy	EUR/HIC	NA	0.768	1	1	Pap test every 3 years	Pap	3 years	20920	2867	25-64
**111**	Du MX, et al.	2015/2010	China	WPR/LMIC	Rural	0.621	1	1	Cervical screening every 3 years	NA	3 years	1013	292	25-55
**112**	Hirth JM, et al.	2016/2012	USA	AMR/HIC	NA	0.839	1	1	Pap test every 3 years	Pap	3 years	4992	4098	21-69

Study ID	Author	Year published/ Year investigated	Country	WHO/WB region	Place	SDI	Data source [a]	Study design [b]	Definition of adherence	Screening method	Screening interval	Sample size [c]	Adherent women	Age range (year)
**113**	Crawford A, et al.	2016/2014	USA	AMR/HIC	NA	0.845	1	1	Pap test every 3 years; Pap test and HPV test every 5 years	Pap	3 years	66402	60371	40-65
**114A**	Shneyderman Y, et al.	2016/2003	USA	AMR/HIC	NA	0.809	1	1	Pap test every 3 years	Pap	3 years	3052	2458	18-65
**114B**	Shneyderman Y, et al.	2016/2007	USA	AMR/HIC	NA	0.814	1	1	Pap test every 3 years	Pap	3 years	3386	2759	18-65
**115**	Valdovinos C, et al.	2016/2010	USA/	AMR/HIC	NA	0.832	1	1	Pap test every year	Pap	1 year	3095	2222	18-74
**116**	Urrutia MT, et al.	2016/2011	Chile	AMR/HIC	Urban	0.712	1	1	Pap test every 3 years	Pap	3 years	1042	800	25-64
**117**	Bianco A, et al.	2017/2012	Italy	EUR/HIC	NA	0.784	1	1	Pap test every 3 years	Pap	3 years	419	164	25-64
**118**	Comparetto C, et al.	2017/2006	Italy	EUR/HIC	Urban	0.780	2	1	Pap test every 3 years	Pap	3 years	45680	24061	25-64
**119A**	Parekh N, et al.	2017/2007	USA	AMR/HIC	NA	0.814	2	1	Pap test every year for women aged 21-30 years; every 2-3 years for women aged ≥30 years	Pap	1 year (<30 years old)/2-3 years (≥30 years old)	14864	5657	18-64
**119B**	Parekh N, et al.	2017/2010	USA	AMR/HIC	NA	0.832	2	1	Pap test every 2 years for women aged <30 years;	Pap	2 years (<30 years old)/3	14786	1299	18-64

Study ID	Author	Year published/ Year investigated	Country	WHO/WB region	Place	SDI	Data source [a]	Study design [b]	Definition of adherence	Screening method	Screening interval	Sample size [c]	Adherent women	Age range (year)
									every 3 years for women aged ≥30 years		years (≥30 years old)			
**120**	Leinonen MK, et al.	2017/2010	Norway	EUR/HIC	NA	0.885	2	1	Pap test every 5 years	Pap	5 years	1309679	863042	25-69
**121**	White A, et al.	2017/2015	USA	AMR/HIC	NA	0.849	1	1	Pap test every 3 years; Pap test and HPV test every 5 years	Pap/Pap and HPV test	3 years/5 years	10477	8696	21-65
**122**	Tung WC, et al.	2017/2015	USA	AMR/HIC	NA	0.849	1	1	Pap test every 3 years	Pap	3 years	121	79	21-65
**123**	Cadet TJ, et al.	2017/2008	USA	AMR/HIC	NA	0.819	1	1	Pap test every 2 years	Pap	2 years	243	141	≥54
**124**	Lofters AK, et al.	2017/2015	Canada	AMR/HIC	NA	0.864	1	1	Pap test every 3 years	Pap	3 years	3997	3146	21-69
**125**	Zorogastua K, et al.	2017/2012	USA	AMR/HIC	NA	0.839	1	1	Pap test every 3 years	Pap	3 years	130	94	21-
**126**	Chang H K, et al.	2017/2011	South Korea	WPR/HIC	NA	0.846	1	1	Pap test every 2 years	Pap	2 years	3734	1730	15-39
**127**	Cofie LE, et al.	2018/2015	USA	AMR/HIC	NA	0.849	1	1	Pap test every 3 years	Pap	3 years	20080	16867	21-65
**128**	Luque JS, et al.	2018/2014	USA	AMR/HIC	NA	0.845	1	1	Pap test every 3 years	Pap	3 years	2822	2307	21-65
**129**	Rendle KA, et al.	2018/2013	USA	AMR/HIC	NA	0.842	2	1	Cervical screening every 2.5-3.5 years	HPV test and Pap	2.5-3.5 years	491588	278918	30-64

Study ID	Author	Year published/ Year investigated	Country	WHO/WB region	Place	SDI	Data source [a]	Study design [b]	Definition of adherence	Screening method	Screening interval	Sample size [c]	Adherent women	Age range (year)
**130**	Petrelli A, et al.	2018/2013	Italy	EUR/HIC	NA	0.787	1	1	Pap test every 3 years	Pap	3 years	32831	20388	25-64
**131**	Han J, et al.	2018/2017	USA	AMR/HIC	NA	0.856	1	1	Pap test every 3 years	Pap	3 years	742	632	40-64
**132**	Restivo V, et al.	2018/2016	Italy	EUR/HIC	NA	0.794	1	1	Pap test every 3 years	Pap	3 years	365	243	25-64
**133**	Luque JS, et al.	2018/2016	USA	AMR/HIC	NA	0.853	1	1	Pap test every 3 years	Pap	3 years	196	166	21-64
**134A**	Petkeviciene J, et al.	2018/2006	Lithuania	EUR/HIC	Both	0.765	1	1	Pap test every 3 years	Pap	3 years	820	492	25-64
**134B**	Petkeviciene J, et al.	2018/2008	Lithuania	EUR/HIC	Both	0.782	1	1	Pap test every 3 years	Pap	3 years	807	503	25-64
**134C**	Petkeviciene J, et al.	2018/2010	Lithuania	EUR/HIC	Both	0.797	1	1	Pap test every 3 years	Pap	3 years	1008	715	25-64
**134D**	Petkeviciene J, et al.	2018/2012	Lithuania	EUR/HIC	Both	0.808	1	1	Pap test every 3 years	Pap	3 years	857	606	25-64
**134E**	Petkeviciene J, et al.	2018/2014	Lithuania	EUR/HIC	Both	0.817	1	1	Pap test every 3 years	Pap	3 years	756	561	25-64

Study ID	Author	Year published/ Year investigated	Country	WHO/WB region	Place	SDI	Data source [a]	Study design [b]	Definition of adherence	Screening method	Screening interval	Sample size [c]	Adherent women	Age range (year)
**135**	Moreno PI, et al.	2019/2010	USA	AMR/HIC	NA	0.832	1	1	Pap test every 2 years	Pap	2 years	2960	2190	18-74
**136**	Lucas-Wright A, et al.	2019/2014	USA	AMR/HIC	NA	0.845	1	1	Pap test every 3 years	Pap	3 years	257	226	18-65
**137**	MacLaughlin KL, et al.	2019/2011	USA	AMR/HIC	Rural	0.835	2	1	Cervical screening every 3 years for women aged 25-49 years; every 5 years for women aged 50–64 years	Pap/Pap and HPV test	3 years (Pap)/5 years (Pap and HPV test)	37123	22933	21-65
**138**	Kaso M, et al.	2019/2013	Japan	WPR/HIC	NA	0.855	1	1	Cervical screening every 2 years	NA	2 years	49217	19545	20-39
**139**	Perez CM, et al.	2020/2015	USA	AMR/HIC	Rural	0.849	1	1	Pap test every 3 years	Pap	3 years	536	421	21-64
**140**	Charkhchi P, et al.	2020/2016	USA	AMR/HIC	NA	0.853	1	1	Pap test every 3 years; Pap and HPV test every 5 years	Pap/Pap and HPV test (30-65 years old)	3 years/5 years (30-65 years old)	113883	92815	21-64
**141**	Abar B, et al.	2020/2015	USA	AMR/HIC	Both	0.849	1	1	Pap test every 3 years; Pap and HPV test every 5 years	Pap/Pap and HPV test	3 years (21-65 years old)/5 years (30-65 years old)	10128	8920	21-65

Study ID	Author	Year published/ Year investigated	Country	WHO/WB region	Place	SDI	Data source [a]	Study design [b]	Definition of adherence	Screening method	Screening interval	Sample size [c]	Adherent women	Age range (year)
**142**	Maxwell AE, et al.	2020/2017	USA	AMR/HIC	NA	0.856	1	1	Pap test every 3 years; Pap and HPV test every 5 years	Pap/Pap and HPV test	3years/5years	137	109	50-65
**143**	Zamorano-Leon JJ, et al.	2020/2017	Spain	EUR/HIC	NA	0.761	1	1	Pap test every 3 years	Cervical cytology	3 years	7855	5734	25-65
**144A**	Garrido CO, et al.	2020/2008	USA	MR/HIC	NA	0.819	1	1	Pap test every 3 years	Pap	3 years	6505	5873	21-29
**144B**	Garrido CO, et al.	2020/2010	USA	AMR/HIC	NA	0.832	1	1	Pap test every 3 years	Pap	3 years	5084	4485	21-29
**144C**	Garrido CO, et al.	2020/2012	USA	AMR/HIC	NA	0.839	1	1	Pap test every 3 years	Pap	3 years	8403	7134	21-29
**144D**	Garrido CO, et al.	2020/2014	USA	AMR/HIC	NA	0.845	1	1	Pap test every 3 years	Pap	3 years	7221	5856	21-29
**144E**	Garrido CO, et al.	2020/2016	USA	AMR/HIC	NA	0.853	1	1	Pap test every 3 years	Pap	3 years	6507	4988	21-29
**144F**	Garrido CO, et al.	2020/2018	USA	AMR/HIC	NA	0.858	1	1	Pap test every 3 years	Pap	3 years	2455	1730	21-29
**145**	Wilding S, et al.	2020/2015	UK	EUR/HIC	NA	0.837	1	1	Cervical screening every 3 years for women aged 25-49 years; every 5 years for women aged 50–64 years	NA	3 years (25-49 years old)/5 years (30-65 years old)	145	98	25-65
**146**	Franck JE, et al.	2020/2014	France	EUR/HIC	NA	0.819	1	1	Pap test every 4 years	Pap	4 years	6182	4595	54-65
**147**	Lee HY, et al.	2020/2015	USA	AMR/HIC	NA	0.849	1	1	Pap test every 3 years	Pap	3 years	230	105	21-85

Study ID	Author	Year published/ Year investigated	Country	WHO/WB region	Place	SDI	Data source [a]	Study design [b]	Definition of adherence	Screening method	Screening interval	Sample size [c]	Adherent women	Age range (year)
**148**	Barrera-Castillo M, et al.	2020/2012	Spain	EUR/HIC	NA	0.749	1	1	Pap test every 3 years	Pap	3 years	8944	6297	18-75
**149**	Deguara M, et al.	2020/2017	Malta	EUR/HIC	NA	0.793	1	1	Pap test every 3 years	Pap	3 years	407	281	25-64
**150**	dos Santos NLF, et al.	2020/2018	Brazil	AMR/LMIC	Urban	0.636	1	1	Pap test every 3 years	Pap	3 years	502	336	18-60
**151**	Malone C, et al.	2021/2015	USA	AMR/HIC	NA	0.849	2	1	Pap test every 3.4 years	Pap	3.4 years	48711	45359	30-64
**152**	Pelullo CP, et al.	2021/2019	Italy	EUR/HIC	Urban	0.801	1	1	Pap test every 3 years	Pap	3 years	706	394	28-67
**153**	Ishii K, et al.	2021/2016	Japan	WPR/HIC	NA	0.862	1	1	Pap test every 2 years	Pap	2 years	115254	51823	40-69

Abbreviations: AFR, African Region; AMR, Region of the Americas; EUR, European Region; HIC, High-income country; HPV, human papillomavirus; LMIC, Low-income and middle-income country; NA, not available; Pap, Papanicolaou smear; SDI, Socio-Demographic Index; SEAR, South-East Asia Region; WB, World Bank; WHO, World Health Organization; WPR, Western Pacific Region.

[a] 1: self-reporting, 2: objective-recording. [b] 1: retrospective, 2: prospective. [c] The number of women who were recommended for cervical cancer screening in each study.

Table S7 Quality scores for assessing the risk of bias for the included studies

Study ID	Study	Quality score					
		Sample population	Sample size	Participation	Outcome assessment	Analytical methods	Total scores
1	Cohen MM, et al. (1992)	2	1	1	2	2	8
2	Lantz PM, et al. (1997)	2	1	1	1	1	6
3	Risendal B, et al. (1999)	1	1	2	1	2	7
4	Fernandez ME, et al. (1999)	2	1	0	1	1	5
5	Rohlfs I, et al. (1999)	2	2	1	1	2	8
6	Taylor VM, et al. (1999)	1	1	1	1	2	6
7	Maxwell AE, et al. (2000)	1	1	0	1	2	5
8	Ramirez AG, et al. (2000)	2	1	1	1	2	7
9	Maxwell CJ, et al. (2001)	2	1	2	1	2	8
10	Do HH, et al. (2001)	1	1	0	1	2	5
11	Jirojwong S, et al. (2001)	1	1	2	1	1	6
12	Lee J, et al. (2002)	1	1	0	1	2	5
13	Cyrus-David MS, et al. (2002)	2	1	0	1	2	6
14	Amonkar MM, et al. (2002)	2	1	0	1	2	6
15	Fernandez-Esquer ME, et al. (2003)	2	1	2	1	1	7
16	Selvin E, et al. (2003)	2	1	1	1	2	7
17	Juon HS, et al. (2003)	1	1	2	1	2	7
18	Miedema BB, et al. (2003)	1	2	0	1	1	5
19	Luengo Matos S, et al. (2003)	2	2	1	1	2	8
20	Hou SI, et al. (2003)	1	1	1	1	1	5
21	Taylor VM, et al. (2004)	1	1	2	1	2	7
22	Coronado GD, et al. (2004)	2	1	1	1	1	6
23	Insinga RP, et al. (2004)	2	1	0	2	1	6
24	Dettenborn L, et al. (2004)	2	1	0	1	1	5
25	Borrayo EA, et al. (2004)	1	1	1	1	1	5
26	Bazargan M, et al. (2004)	2	1	1	1	1	6
27	Cho IS, et al. (2004)	1	1	0	1	2	5
28	Hoyo C, et al. (2005)	1	1	1	1	1	5
29	De Alba I, et al. (2005)	2	1	0	1	2	6
30	Kwak MS, et al. (2005)	2	1	0	1	2	6
31	Giorgi Rossi P, et al. (2006)	2	2	0	1	1	6

Study ID	Study	Quality score					
		Sample population	Sample size	Participation	Outcome assessment	Analytical methods	Total scores
32	Carruth AK, et al. (2006)	2	1	2	1	2	8
33	Liao CC, et al. (2006)	2	1	2	1	1	7
34	Lee-Lin F, et al. (2007)	1	2	2	1	1	7
35	Chen LS, et al. (2007)	2	1	0	2	2	7
36	Gao W, et al. (2008)	1	1	2	1	1	6
37	Tsui J, et al. (2008)	1	1	2	1	2	7
38	Puig-Tintore LM, et al. (2008)	2	1	0	1	2	6
39	Ginde AA, et al. (2008)	2	1	1	1	1	6
40	Tung WC, et al. (2008)	1	1	0	1	1	4
41	Nelson W, et al. (2009)	2	1	0	1	2	6
42	Ma GX, et al. (2009)	2	2	0	1	1	6
43	Dimitrakaki C, et al. (2009)	2	1	1	1	2	7
44	Amankwah E, et al. (2009)	2	1	1	1	1	6
45	Taylor VM, et al. (2009)	1	1	1	1	2	6
46	Sun YG, et al. (2009)	2	1	2	1	1	7
47	Martin-Lopez R, et al. (2010)	2	1	0	1	2	6
48	Zhou J, et al. (2010)	2	1	1	1	1	6
49	Byrne MM, et al. (2010)	2	1	0	1	2	6
50	Lopez-de-Andres A, et al. (2010)	2	2	0	1	2	7
51	Lim JW. (2010)	2	1	0	1	2	6
52	Paskett ED, et al. (2010)	2	1	1	1	2	7
53	Lee K, et al. (2010)	2	1	0	1	1	5
54	Meng RL, et al. (2010)	2	1	0	1	2	6
55	Wang CF, et al. (2010)	2	1	0	1	2	6
56	Lawsin C, et al. (2011)	1	1	0	1	1	4
57	Cheung NW, et al. (2011)	1	1	0	1	1	4
58	Pons-Vigues M, et al. (2011)	2	1	0	1	1	5
59	Tekkel M, et al. (2011)	2	1	0	1	2	6
60	Nuno T, et al. (2011)	2	1	1	1	2	7
61	Hansen BT, et al. (2011)	2	1	0	1	2	6
62	MacLaughlan SD, et al. (2011)	2	1	0	1	2	6
63	Vakfari A, et al. (2011)	1	1	0	1	1	4
64	Acikgoz A, et al. (2011)	1	2	2	1	1	7

Study ID	Study	Quality score					
		Sample population	Sample size	Participation	Outcome assessment	Analytical methods	Total scores
65	Lee M, et al. (2011)	2	2	1	1	2	8
66	Liang Q, et al. (2011)	2	1	2	1	2	8
67	Nguyen-Truong CK, et al. (2012)	1	1	2	1	2	7
68	Worthington C, et al. (2012)	2	1	2	1	1	7
69	Gonzalez P, et al. (2012)	1	1	0	1	1	4
70	Damiani G, et al. (2012)	2	1	0	1	2	6
71	Martin-Lopez R, et al. (2012)	2	1	0	1	2	6
72	Muus KJ, et al. (2012)	2	1	1	1	1	6
73	Olesen SC, et al. (2012)	2	1	0	2	2	7
74	Macedo A, et al. (2012)	2	1	0	1	2	6
75	Ko KD, et al. (2012)	2	1	0	1	2	6
76	Azerkan F, et al. (2012)	2	2	2	2	2	10
77	Aminisani N, et al. (2012)	2	1	2	2	2	9
78	Nuno T, et al. (2012)	2	1	0	1	2	6
79	Shelton RC, et al. (2012)	1	1	0	1	2	5
80	Ashok M, et al. (2012)	2	1	0	1	2	6
81	Leone LA, et al. (2012)	1	1	0	1	2	5
82	Shippee ND, et al. (2012)	1	1	0	2	2	6
83	Aminisani N, et al. (2012)	2	1	1	2	2	8
84	Raz R, et al. (2012)	2	1	1	2	2	8
85	Borges MF, et al. (2012)	2	2	2	1	2	9
86	Almeida CM, et al. (2013)	2	1	1	2	2	8
87	Morrison TB, et al. (2013)	2	1	0	2	1	6
88	Oussaid N, et al. (2013)	2	1	1	1	2	7
89	Cerigo H, et al. (2013)	2	1	2	1	2	8
90	Augusto EF, et al. (2013)	2	2	2	1	2	9
91	Khadilkar A, et al. (2013)	2	1	1	1	2	7
92	Lee M, et al. (2013)	2	1	0	1	2	6
93	Suh M, et al. (2013)	2	1	0	1	2	6
94	Hernández CM, et al. (2014)	0	1	2	1	2	6
95	Tsai RJ, et al. (2014)	2	1	0	1	2	6
96	Adonis L, et al. (2014)	2	1	0	1	2	6
97	Moudatsou MM, et al. (2014)	2	2	2	1	2	9

Study ID	Study	Quality score					
		Sample population	Sample size	Participation	Outcome assessment	Analytical methods	Total scores
98	Kristensson JH, et al. (2014)	2	2	2	2	2	10
99	Arbyn M, et al. (2014)	2	1	0	2	1	6
100	Calo WA (2014)	2	1	0	1	2	6
101	Documet P, et al. (2015)	2	1	0	1	2	6
102	Guo F, et al. (2015)	2	1	1	1	1	6
103	Visanuyothin S, et al. (2015)	2	2	2	1	2	9
104	Ricardo-Rodrigues I, et al. (2015)	2	1	0	1	2	6
105	Miranda-Diaz C, et al. (2015)	2	1	0	1	2	6
106	Gyulai A, et al. (2015)	1	1	2	1	1	6
107	Richard A, et al. (2015)	1	1	0	1	2	5
108	Schoofs J, et al. (2015)	2	1	0	1	2	6
109	Oestensson E, et al. (2015)	1	1	0	1	2	5
110	Barbadoro P, et al. (2015)	1	2	2	1	2	8
111	Du MX, et al. (2015)	1	1	2	1	2	7
112	Hirth JM, et al. (2016)	2	1	0	1	2	6
113	Crawford A, et al. (2016)	2	1	0	1	2	6
114	Shneyderman Y, et al. (2016)	2	1	0	1	2	6
115	Valdovinos C, et al. (2016)	2	1	0	1	2	6
116	Urrutia MT, et al. (2016)	1	2	0	1	1	5
117	Bianco A, et al. (2017)	1	0	2	1	2	6
118	Comparetto C, et al. (2017)	2	1	0	2	1	6
119	Parekh N, et al. (2017)	2	1	0	2	1	6
120	Leinonen MK, et al. (2017)	2	1	2	2	2	9
121	White A, et al. (2017)	2	1	0	1	1	5
122	Tung WC, et al. (2017)	1	1	0	1	1	4
123	Cadet TJ, et al. (2017)	2	1	0	1	2	6
124	Lofters AK, et al. (2017)	2	1	0	1	1	5
125	Zorogastua K, et al. (2017)	1	0	0	1	1	3
126	Chang HK, et al. (2017)	2	1	1	1	2	7
127	Cofie LE, et al. (2018)	2	1	0	1	2	6
128	Luque JS, et al. (2018)	2	1	0	1	2	6
129	Rendle KA, et al. (2018)	2	1	0	2	2	7
130	Petrelli A, et al. (2018)	2	1	0	1	2	6

Study ID	Study	Quality score					
		Sample population	Sample size	Participation	Outcome assessment	Analytical methods	Total scores
131	Han J, et al. (2018)	2	1	0	1	2	6
132	Restivo V, et al. (2018)	2	1	1	1	2	7
133	Luque JS, et al. (2018)	1	1	0	1	1	4
134	Petkeviciene J, et al. (2018)	2	1	0	1	2	6
135	Moreno PI, et al. (2019)	2	1	0	1	2	6
136	Lucas-Wright A, et al. (2019)	1	1	0	1	1	4
137	MacLaughlin KL, et al. (2019)	2	1	2	2	2	9
138	Kaso M, et al. (2019)	2	1	0	1	2	6
139	Perez CM, et al. (2020)	2	1	2	1	2	8
140	Charkhchi P, et al. (2020)	2	1	0	1	2	6
141	Abar B, et al. (2020)	0	1	2	1	1	5
142	Maxwell AE, et al. (2020)	1	1	0	1	1	4
143	Zamorano-Leon JJ, et al. (2020)	2	1	0	1	2	6
144	Garrido CO, et al. (2020)	2	1	0	1	2	6
145	Wilding S, et al. (2020)	1	1	0	1	2	5
146	Franck JE, et al. (2020)	2	1	0	1	2	6
147	Lee HY, et al. (2020)	2	2	0	1	2	7
148	Barrera-Castillo M, et al. (2020)	2	1	0	1	2	6
149	Deguara M, et al. (2020)	2	2	2	1	2	9
150	dos Santos NLF, et al. (2020)	2	1	0	1	1	5
151	Malone C, et al. (2021)	2	1	0	1	2	6
152	Pelullo CP, et al. (2021)	2	1	0	1	2	6
153	Ishii K, et al. (2021)	2	1	1	1	1	6

Table S9 Meta-analyses of associated factors for adherence to cervical cancer screening

Associated factor	Study ID	Study	Pooled adherence rate
**Associated factor 1: Marital status**			
**Married vs Not Married (n=15)**			
	1	Cohen MM, et al.	
	7	Maxwell AE, et al.	
	16	Selvin E, et al. (Non-Hispanic White)	
	16	Selvin E, et al. (Non-Hispanic Black)	
	16	Selvin E, et al. (Hispanic)	
	17	Juon HS, et al.	
	32	Carruth AK, et al.	
	43	Dimitrakaki C, et al.	
	60	Nuno T, et al.	
	82	Shippee ND, et al.	
	98	Kristensson JH, et al.	
	120	Leinonen MK, et al. (<45 years old)	
	120	Leinonen MK, et al. (≥45 years old)	
	117	Bianco A, et al.	
	132	Restivo V, et al.	

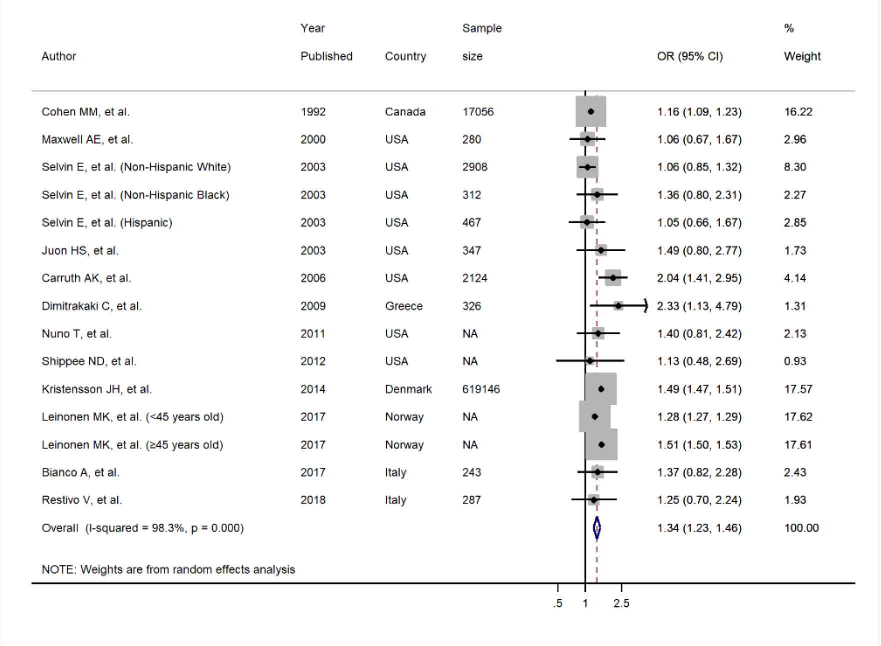

Associated factor	Study ID	Study	Pooled adherence rate
**Married vs Single (n=12)**			
	5	Rohlfs I, et al.	
	12	Lee J, et al.	
	13	Cyrus-David MS, et al.	
	47	Martin-Lopez R, et al.	

Associated factor	Study ID	Study	Pooled adherence rate
	70	Damiani G, et al.	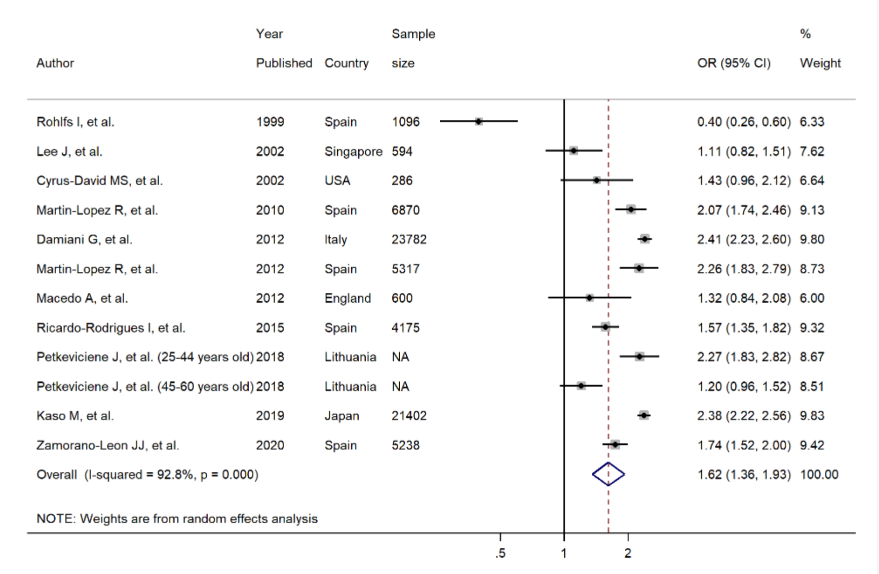
	71	Martin-Lopez R, et al.	
	74	Macedo A, et al.	
	104	Ricardo-Rodrigues I, et al.	
	134	Petkeviciene J, et al. (25-44 years old)	
	134	Petkeviciene J, et al. (45-60 years old)	
	138	Kaso M, et al.	
	143	Zamorano-Leon JJ, et al.	
**Partnered vs No partnered (n=9)**			
	37	Tsui J, et al.	
	75	Ko KD, et al.	
	79	Shelton RC, et al. (Mexico)	
	79	Shelton RC, et al. (Puerto Rico)	
	79	Shelton RC, et al. (Central and South America)	
	103	Visanuyothin S, et al.	
	106	Gyulai A, et al.	
	128	Luque JS, et al.	
	140	Charkhchi P, et al.	

Associated factor	Study ID	Study	Pooled adherence rate
			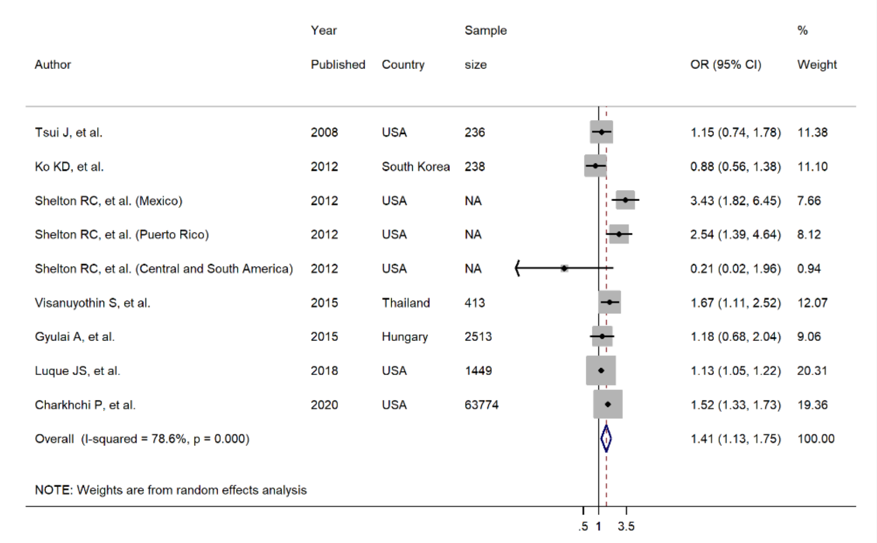
**Currently married vs Never married (n=3)**			
	10	Do HH, et al.	
	21	Taylor VM, et al.	
	45	Taylor VM, et al.	

Associated factor	Study ID	Study	Pooled adherence rate
**Previously married vs Never married (n=3)**			
	10	Do HH, et al.	
	45	Taylor VM, et al.	
	21	Taylor VM, et al.	

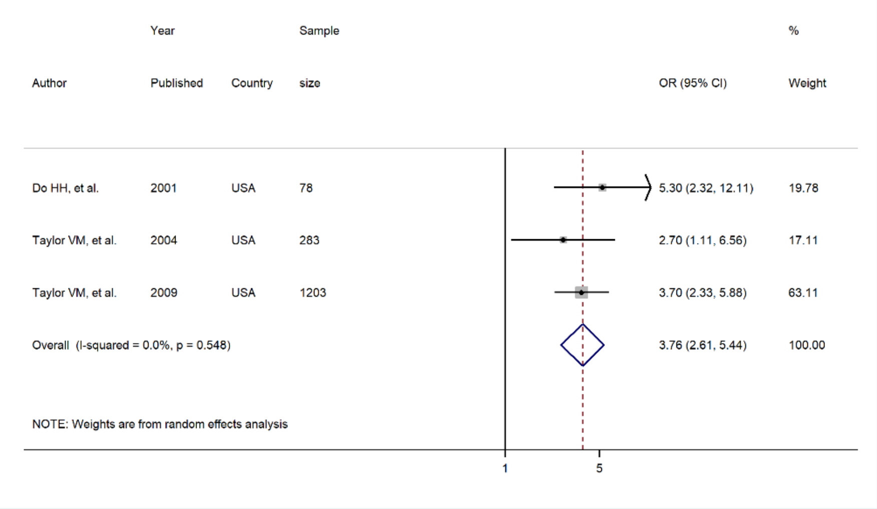

Associated factor	Study ID	Study	Pooled adherence rate
			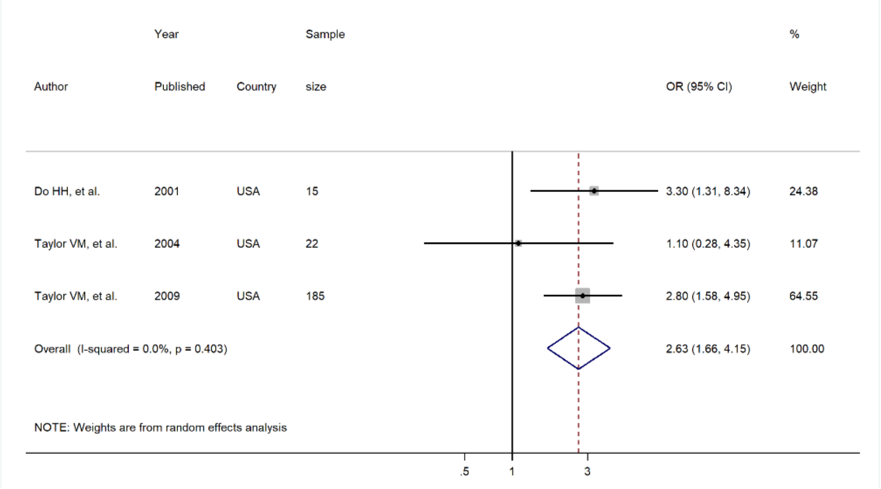
**Married vs Divorced/Widowed/Separated (n=5)**	14<br>66<br>74<br>120<br>120	Amonkar MM, et al.<br>Liang Q, et al.<br>Macedo A, et al.<br>Leinonen MK, et al. (<45 years old)<br>Leinonen MK, et al. (≥45 years old)	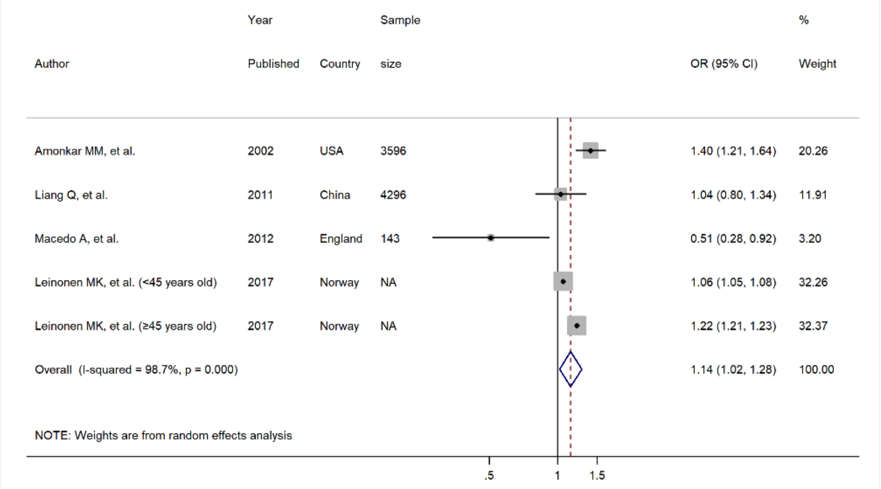

Associated factor	Study ID	Study	Pooled adherence rate
**Divorced/Widowed/Separated vs Single (n=5)**			
	47	Martin-Lopez R, et al.	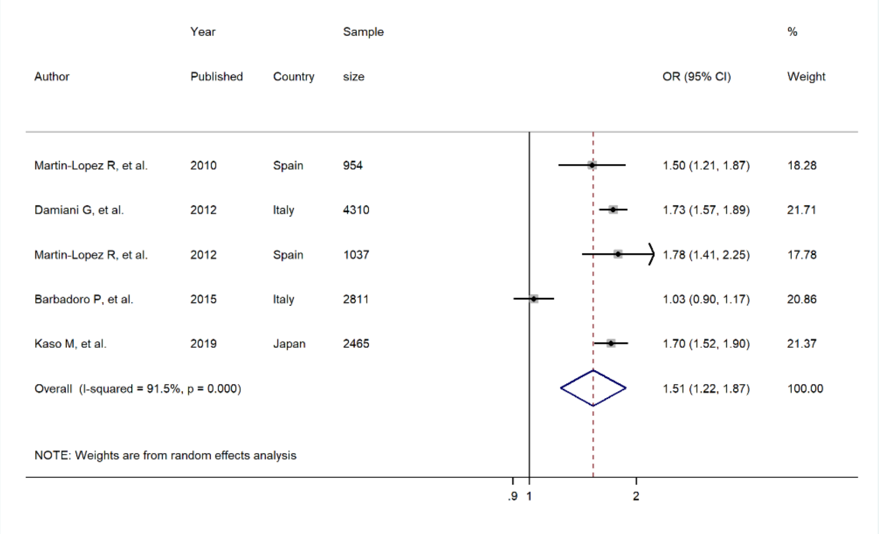
	70	Damiani G, et al.	
	71	Martin-Lopez R, et al.	
	110	Barbadoro P, et al.	
	138	Kaso M, et al.	
**Associated factor 2: Education level**			
**High school vs less than high school (n=53)**			
	5	Rohlfs I, et al.	
	7	Maxwell AE, et al.	
	8	Ramirez AG, et al. (<40 years old)	
	8	Ramirez AG, et al. (≥40 years old)	
	9	Maxwell CJ, et al.	
	13	Cyrus-David MS, et al.	
	14	Amonkar MM, et al.	
	16	Selvin E, et al. (Non-Hispanic White)	
	16	Selvin E, et al. (Non-Hispanic Black)	
	16	Selvin E, et al. (Hispanic)	

Associated factor	Study ID	Study	Pooled adherence rate
	27	Cho IS, et al.	
	29	De Alba I, et al.	
	30	Kwak MS, et al.	
	32	Carruth AK, et al.	
	37	Tsui J, et al.	
	41	Nelson W, et al.	
	43	Dimitrakaki C, et al.	
	47	Martin-Lopez R, et al.	
	50	Lopez-de-Andres A, et al.	
	51	Lim JW. (Latina)	
	51	Lim JW. (Asian)	
	60	Nuno T, et al.	
	61	Hansen BT, et al.	
	66	Liang Q, et al.	
	70	Damiani G, et al.	
	71	Martin-Lopez R, et al.	
	85	Borges MF, et al.	
	89	Cerigo H, et al.	
	91	Khadilkar A, et al.	
	92	Lee M, et al. (1998)	
	92	Lee M, et al. (2001)	
	92	Lee M, et al. (2005)	
	92	Lee M, et al. (2008)	
	92	Lee M, et al. (2010)	
	98	Kristensson JH, et al.	
	104	Ricardo-Rodrigues I, et al.	
	106	Gyulai A, et al.	

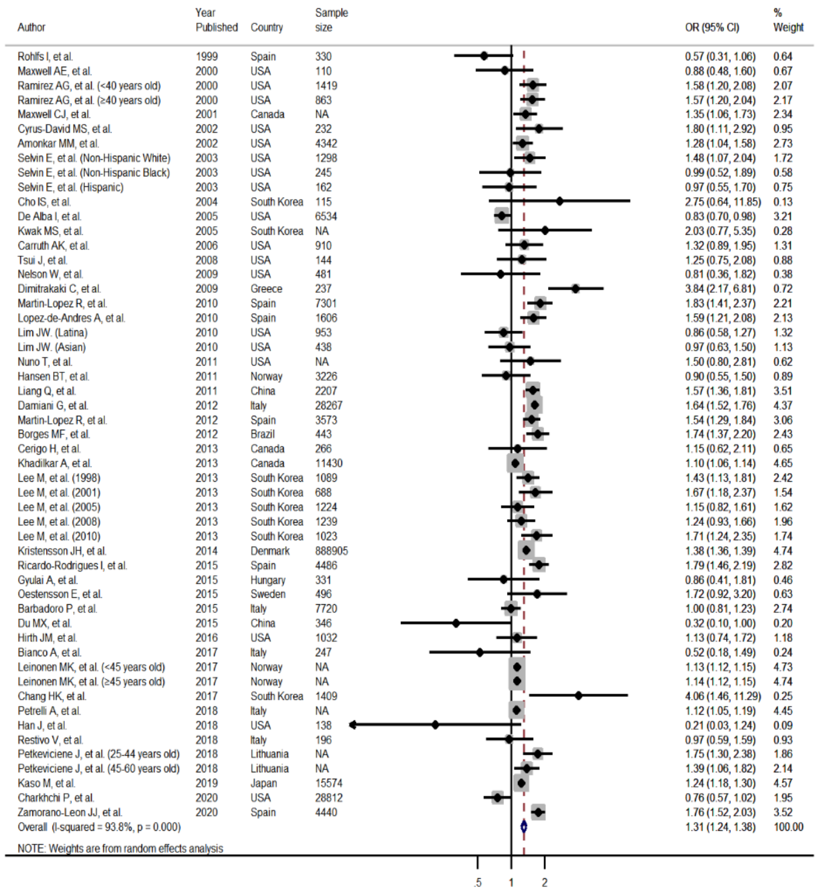

Associated factor	Study ID	Study	Pooled adherence rate
	109	Oestensson E, et al.	
	110	Barbadoro P, et al.	
	111	Du MX, et al.	
	112	Hirth JM, et al.	
	117	Bianco A, et al.	
	120	Leinonen MK, et al. (<45 years old)	
	120	Leinonen MK, et al. (≥45 years old)	
	126	Chang HK, et al.	
	130	Petrelli A, et al.	
	131	Han J, et al.	
	132	Restivo V, et al.	
	134	Petkeviciene J, et al. (25-44 years old)	
	134	Petkeviciene J, et al. (45-60 years old)	
	138	Kaso M, et al.	
	140	Charkhchi P, et al.	
	143	Zamorano-Leon JJ, et al.	
**Higher than high school vs less than high school (n=47)**			
	5	Rohlfs I, et al.	
	7	Maxwell AE, et al.	
	9	Maxwell CJ, et al.	
	12	Lee J, et al.	
	13	Cyrus-David MS, et al.	
	14	Amonkar MM, et al.	
	16	Selvin E, et al. (Non-Hispanic White)	
	16	Selvin E, et al. (Non-Hispanic Black)	
	16	Selvin E, et al. (Hispanic)	

Associated factor	Study ID	Study	Pooled adherence rate
	29	De Alba I, et al.	
	30	Kwak MS, et al.	
	32	Carruth AK, et al.	
	37	Tsui J, et al.	
	41	Nelson W, et al.	
	43	Dimitrakaki C, et al.	
	47	Martin-Lopez R, et al.	
	50	Lopez-de-Andres A, et al.	
	51	Lim JW. (Latina)	
	51	Lim JW. (Asian)	
	60	Nuno T, et al.	
	61	Hansen BT, et al.	
	71	Martin-Lopez R, et al.	
	92	Lee M, et al. (1998)	
	92	Lee M, et al. (2001)	
	92	Lee M, et al. (2005)	
	92	Lee M, et al. (2008)	
	92	Lee M, et al. (2010)	
	98	Kristensson JH, et al.	
	101	Documet P, et al.	
	104	Ricardo-Rodrigues I, et al.	
	106	Gyulai A, et al.	
	109	Oestensson E, et al.	
	110	Barbadoro P, et al.	
	111	Du MX, et al.	
	112	Hirth JM, et al.	
	117	Bianco A, et al.	

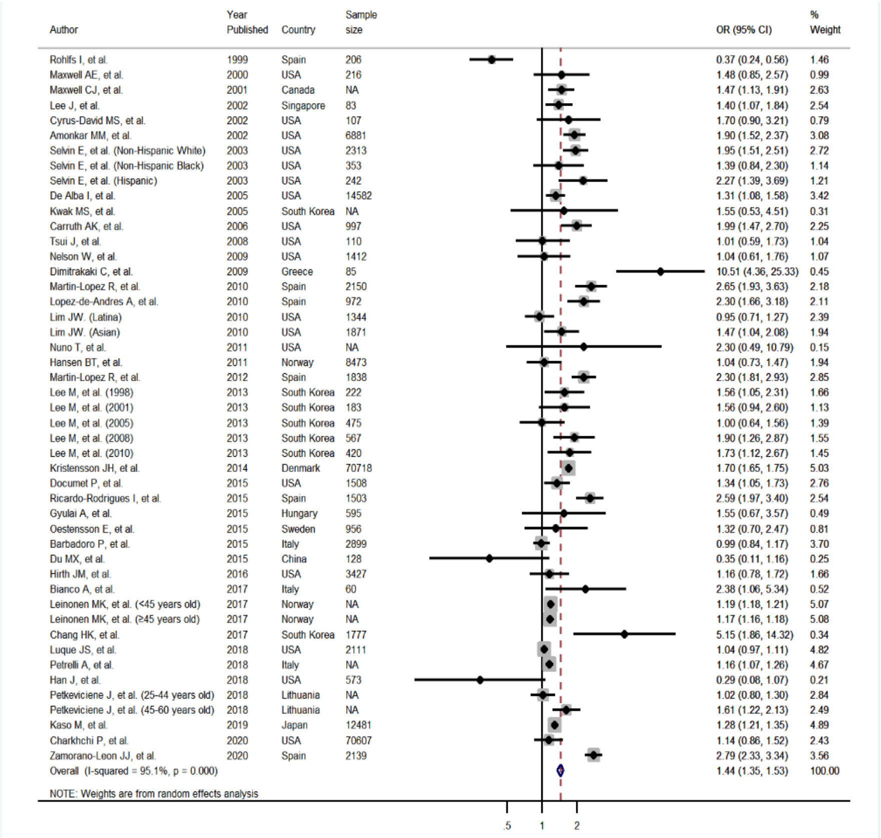

Associated factor	Study ID	Study	Pooled adherence rate
	120	Leinonen MK, et al. (<45 years old)	
	120	Leinonen MK, et al. (≥45 years old)	
	126	Chang HK, et al.	
	128	Luque JS, et al.	
	130	Petrelli A, et al.	
	131	Han J, et al.	
	134	Petkeviciene J, et al. (25-44 years old)	
	134	Petkeviciene J, et al. (45-60 years old)	
	138	Kaso M, et al.	
	140	Charkhchi P, et al.	
	143	Zamorano-Leon JJ, et al.	
**Associated factor 3: Healthcare**			
**Healthcare coverage (n=4)**			
	8	Ramirez AG, et al. (<40 years old)	
	8	Ramirez AG, et al. (≥40 years old)	
	14	Amonkar MM, et al.	
	49	Byrne MM, et al.	

Associated factor	Study ID	Study	Pooled adherence rate
			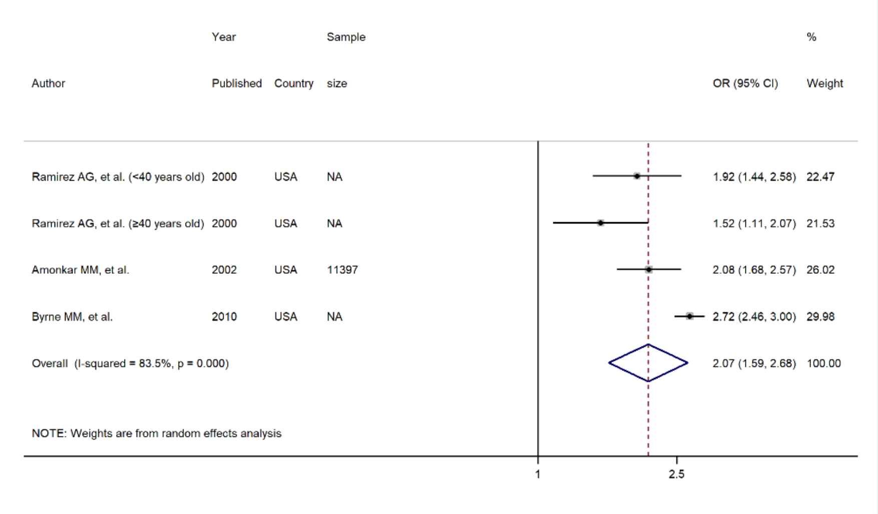
**Insurance (n=8)**			
	7	Maxwell AE, et al.	
	30	Kwak MS, et al.	
	45	Taylor VM, et al.	
	51	Lim JW. (Latina)	
	51	Lim JW. (Asian)	
	60	Nuno T, et al.	
	111	Du MX, et al.	
	131	Han J, et al.	

Associated factor	Study ID	Study	Pooled adherence rate
			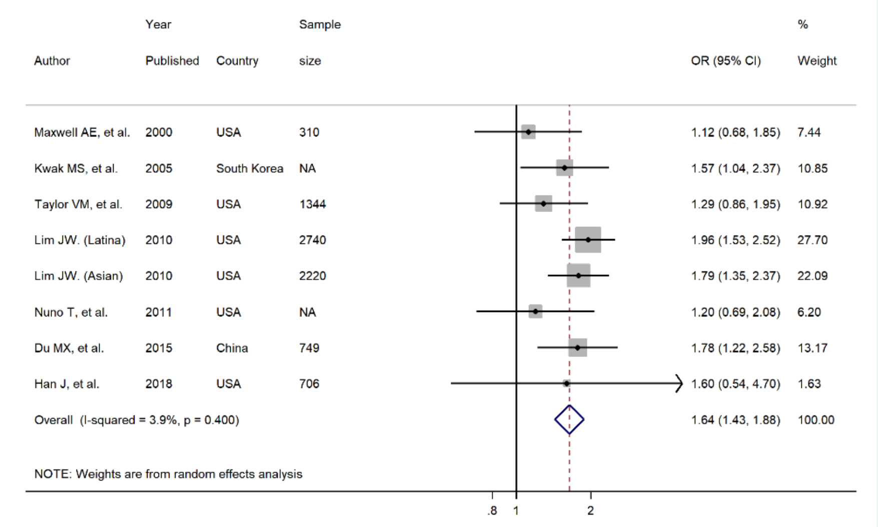
**Associated factor 4: Employment status (Employed vs Unemployed) (n=20)**			
	5	Rohlfs I, et al.	
	13	Cyrus-David MS, et al.	
	17	Juon HS, et al.	
	51	Lim JW. (Latina)	
	51	Lim JW. (Asian)	
	60	Nuno T, et al.	
	70	Damiani G, et al.	
	73	Olesen, SC, et al.	
	85	Borges MF, et al.	
	88	Oussaid N, et al.	
	103	Visanuyothin S, et al.	
	106	Gyulai A, et al.	

Associated factor	Study ID	Study	Pooled adherence rate
	109	Oestensson E, et al.	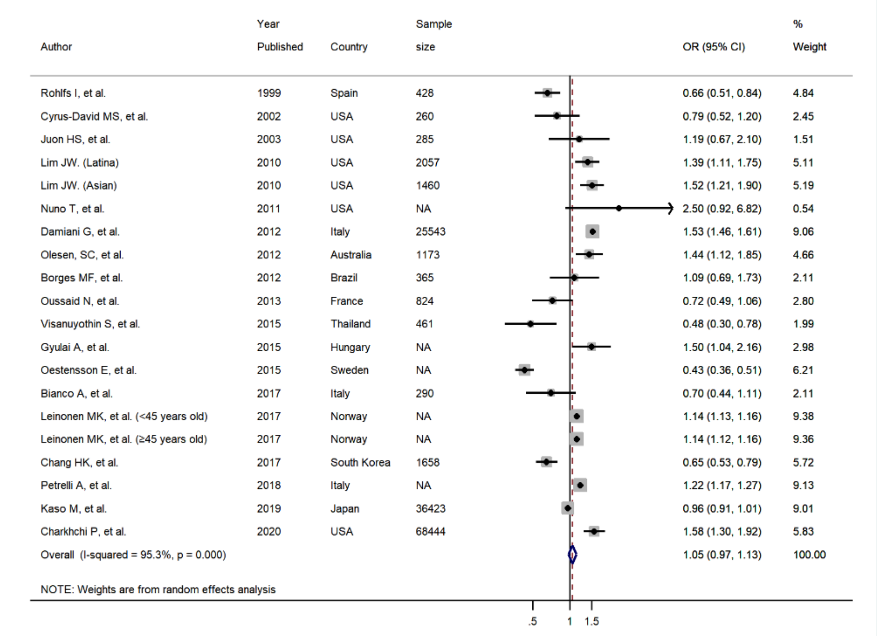
	117	Bianco A, et al.	
	120	Leinonen MK, et al. (<45 years old)	
	120	Leinonen MK, et al. (≥45 years old)	
	126	Chang HK, et al.	
	130	Petrelli A, et al.	
	138	Kaso M, et al.	
	140	Charkhchi P, et al.	
**Associated factor 5: Body-mass index**			
**Obesity: BMI ≥30.0 kg/m² vs Non-obesity (n=9)**			
	14	Amonkar MM, et al.	
	47	Martin-Lopez R, et al.	
	50	Lopez-de-Andres A, et al.	
	71	Martin-Lopez R, et al.	
	73	Olesen, SC, et al.	
	104	Ricardo-Rodrigues I, et al.	
	134	Petkeviciene J, et al. (25-44 years old)	
	134	Petkeviciene J, et al. (45-60 years old)	

Associated factor	Study ID	Study	Pooled adherence rate
	143	Zamorano-Leon JJ, et al.	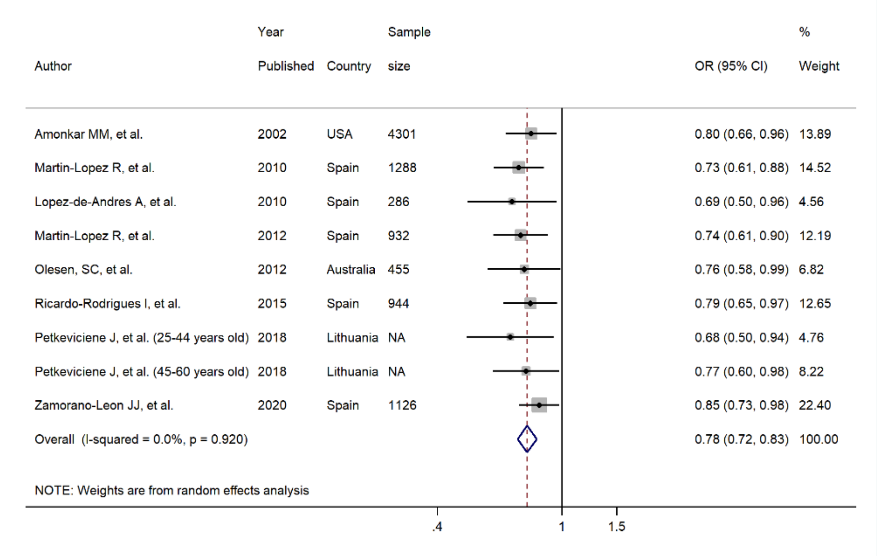
**Underweight: BMI <18.5 kg/m² vs Normal: BMI 18.5-24.9 kg/m² (n=8)**			
	41	Nelson W, et al.	
	51	Lim JW. (Latina)	
	51	Lim JW. (Asian)	
	70	Damiani G, et al.	
	107	Richard A, et al.	
	127	Cofie LE, et al.	
	139	Perez CM, et al.	
	140	Charkhchi P, et al.	

Associated factor	Study ID	Study	Pooled adherence rate
			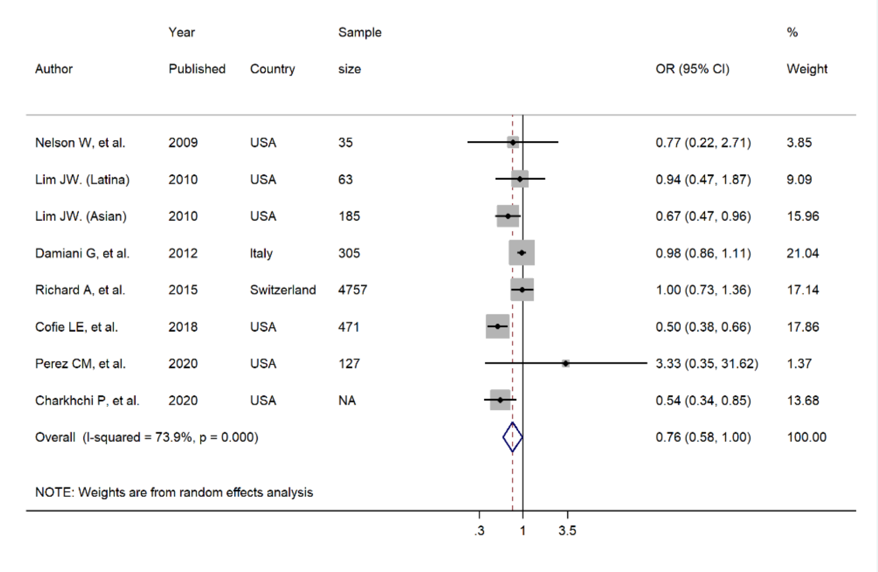
**Overweight: BMI 25.0-30.0 kg/m² vs Normal: BMI 18.5-24.9 kg/m² (n=12)**			
	41	Nelson W, et al.	
	51	Lim JW. (Latina)	
	51	Lim JW. (Asian)	
	59	Tekkel M, et al.	
	70	Damiani G, et al.	
	81	Leone LA, et al.	
	107	Richard A, et al.	
	112	Hirth JM, et al.	
	127	Cofie LE, et al.	
	128	Luque JS, et al.	
	139	Perez CM, et al.	

Associated factor	Study ID	Study	Pooled adherence rate
	140	Charkhchi P, et al.	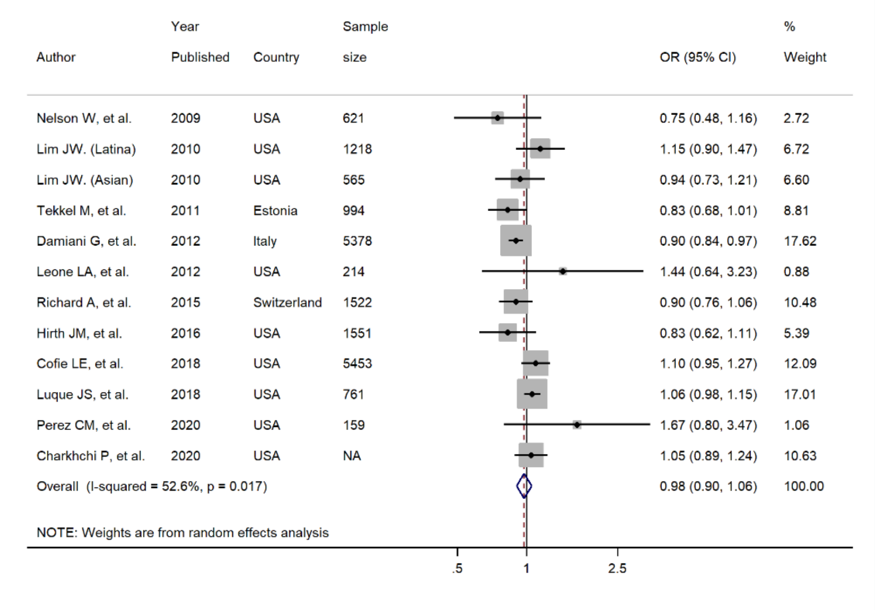
**Obesity: BMI ≥30.0 kg/m² vs Normal: BMI 18.5-24.9 kg/m² (n=12)**			
	41	Nelson W, et al.	
	51	Lim JW. (Latina)	
	51	Lim JW. (Asian)	
	59	Tekkel M, et al.	
	70	Damiani G, et al.	
	81	Leone LA, et al.	
	107	Richard A, et al.	
	112	Hirth JM, et al.	
	127	Cofie LE, et al.	

Associated factor	Study ID	Study	Pooled adherence rate
	128	Luque JS, et al.	
	139	Perez CM, et al.	
	140	Charkhchi P, et al.	

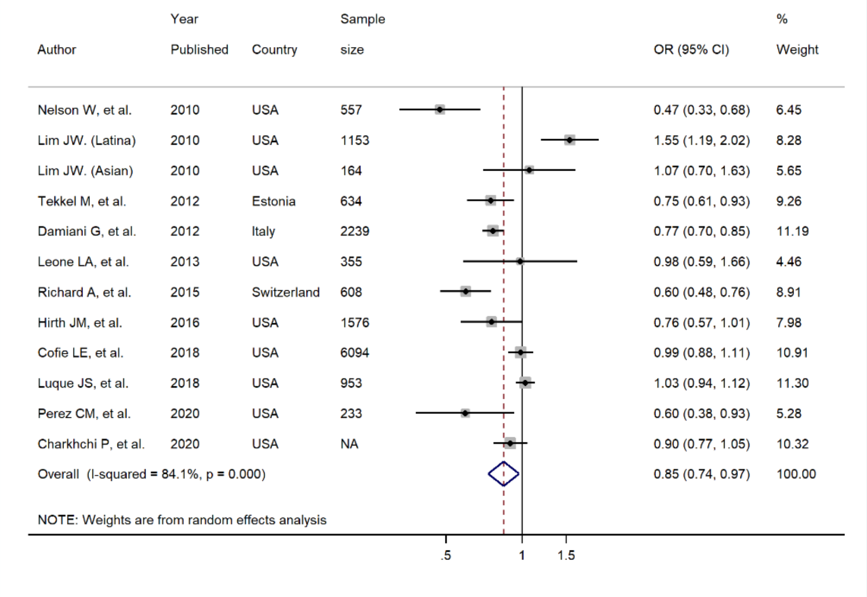

Associated factor 6: Smoking

Associated factor	Study ID	Study	Pooled adherence rate
**Smoker vs Non-smoker (n=14)**			
	14	Amonkar MM, et al.	
	30	Kwak MS, et al.	
	43	Dimitrakaki C, et al.	
	49	Byrne MM, et al.	
	47	Martin-Lopez R, et al.	
	75	Ko KD, et al.	
	82	Shippee ND, et al.	
	88	Oussaid N, et al.	
	106	Gyulai A, et al.	

Associated factor	Study ID	Study	Pooled adherence rate
	110	Barbadoro P, et al.	
	134	Petkeviciene J, et al. (25-44 years old)	
	134	Petkeviciene J, et al. (45-60 years old)	
	143	Zamorano-Leon JJ, et al.	
	140	Charkhchi P, et al.	

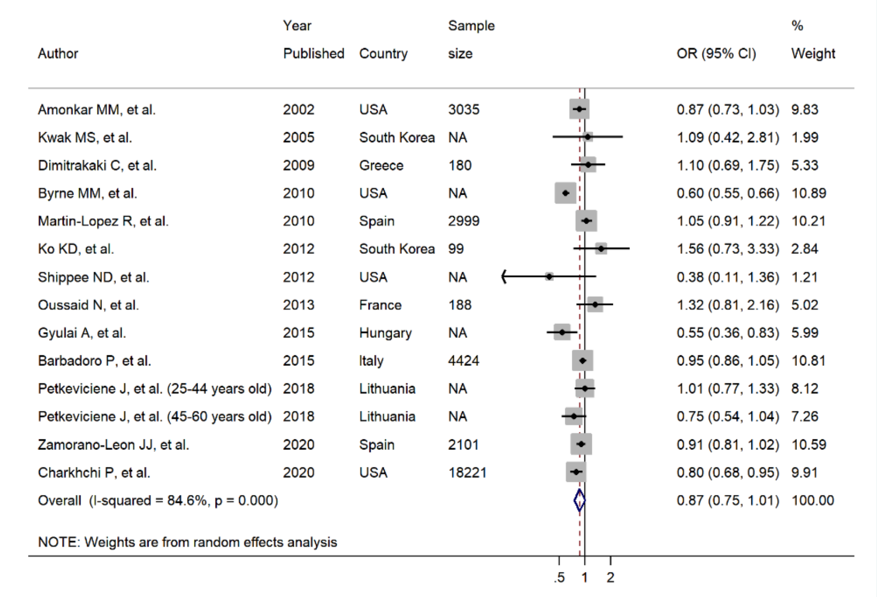

Associated factor	Study ID	Study	Pooled adherence rate
**Current vs Former/Never (n= 5)**			
	9	Maxwell CJ, et al.	
	16	Selvin E, et al. (Non-Hispanic White)	
	16	Selvin E, et al. (Non-Hispanic Black)	
	16	Selvin E, et al. (Hispanic)	
	73	Olesen, SC, et al.	

Associated factor	Study ID	Study	Pooled adherence rate

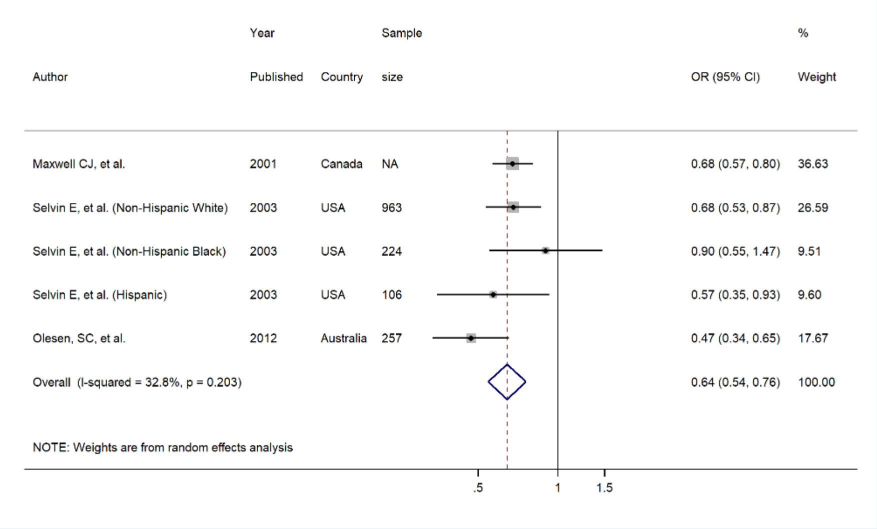

Associated factor	Study ID	Study	Pooled adherence rate
**Current vs Never (n=11)**			
	41	Nelson W, et al.	
	51	Lim JW. (Latina)	
	51	Lim JW. (Asian)	
	61	Hansen BT, et al.	
	62	MacLaughlan SD, et al.	
	70	Damiani G, et al.	
	71	Martin-Lopez R, et al.	
	107	Richard A, et al.	
	126	Chang HK, et al.	
	128	Luque JS, et al.	
	130	Petrelli A, et al.	

Associated factor	Study ID	Study	Pooled adherence rate
			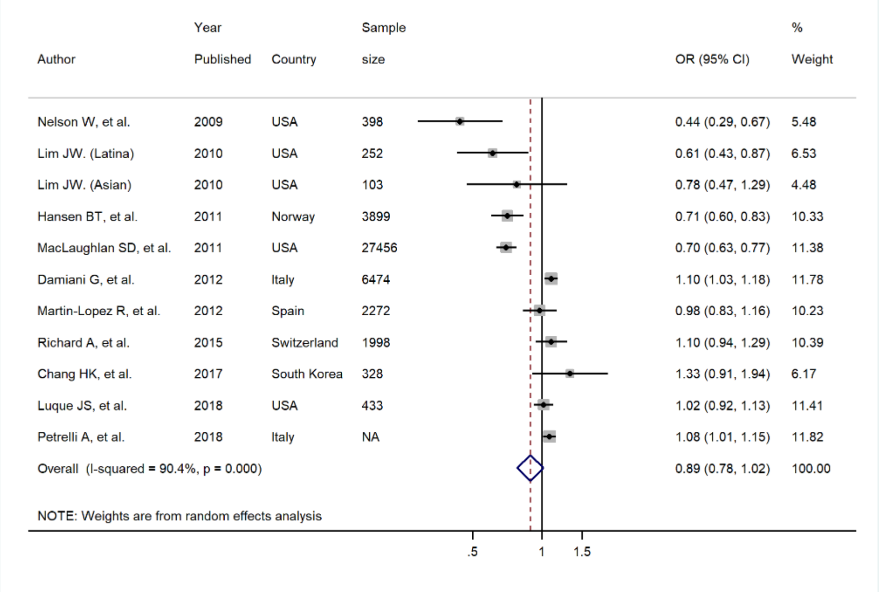
**Former vs Never (n=12)**			
	13	Cyrus-David MS, et al.	
	41	Nelson W, et al.	
	51	Lim JW. (Latina)	
	51	Lim JW. (Asian)	
	61	Hansen BT, et al.	
	62	MacLaughlan SD, et al.	
	70	Damiani G, et al.	
	71	Martin-Lopez R, et al.	
	107	Richard A, et al.	
	126	Chang HK, et al.	

Associated factor	Study ID	Study	Pooled adherence rate
	128	Luque JS, et al.	
	130	Petrelli A, et al.	

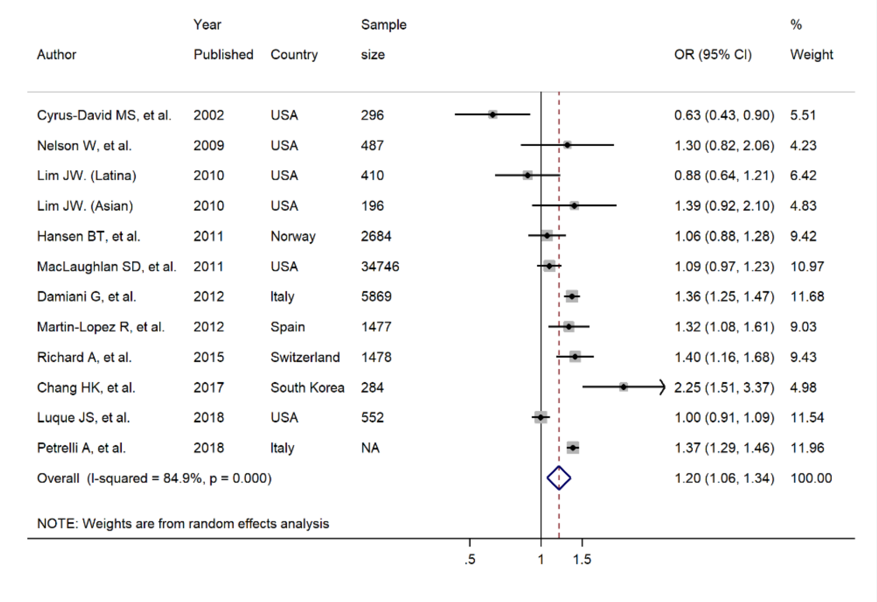

Associated factor 7: Alcohol drinking (n=12)

Associated factor	Study ID	Study	Pooled adherence rate
	30	Kwak MS, et al.	
	51	Lim JW. (Latina)	
	51	Lim JW. (Asian)	
	61	Hansen BT, et al.	
	82	Shippee ND, et al.	
	104	Ricardo-Rodrigues I, et al.	
	107	Richard A, et al.	
	117	Bianco A, et al.	
	134	Petkeviciene J, et al. (25-44 years old)	
	134	Petkeviciene J, et al. (45-60 years old)	

Associated factor	Study ID	Study	Pooled adherence rate
	143	Zamorano-Leon JJ, et al.	
	152	Pelullo CP, et al.	

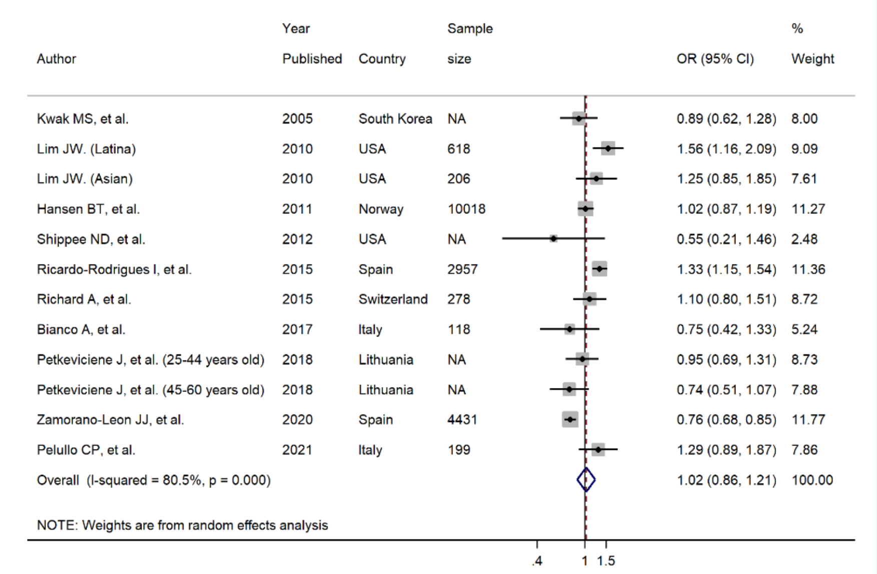

Associated factor 8: Physical activity (n=11)

Associated factor	Study ID	Study	Pooled adherence rate
	30	Kwak MS, et al.	
	47	Martin-Lopez R, et al.	
	49	Byrne MM, et al.	
	50	Lopez-de-Andres A, et al.	
	51	Lim JW. (Latina)	
	51	Lim JW. (Asian)	
	104	Ricardo-Rodrigues I, et al.	
	117	Bianco A, et al.	
	130	Petrelli A, et al.	
	143	Zamorano-Leon JJ, et al.	
	152	Pelullo CP, et al.	

Associated factor	Study ID	Study	Pooled adherence rate
			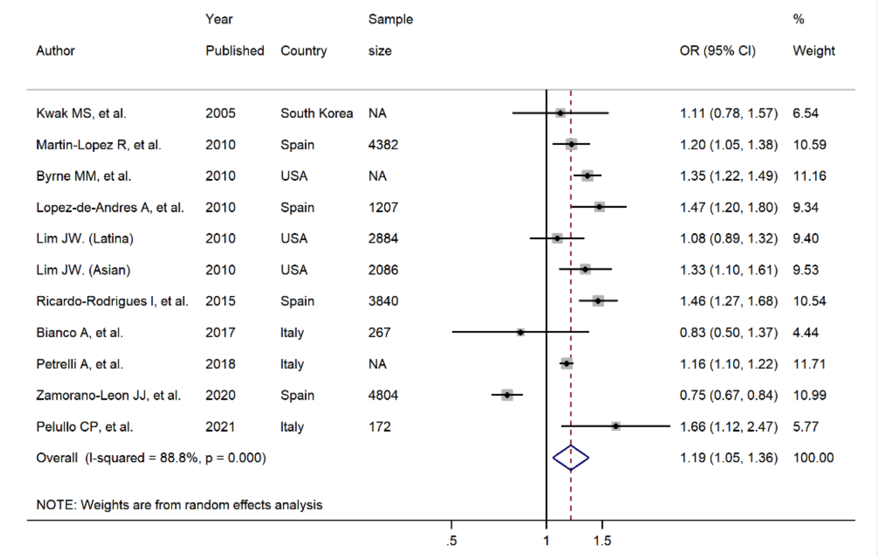
**Associated factor 9: Parity (n=11)**			
	1	Cohen MM, et al.	
	12	Lee J, et al.	
	61	Hansen BT, et al.	
	73	Olesen, SC, et al.	
	88	Oussaid N, et al.	
	89	Cerigo H, et al.	
	103	Visanuyothin S, et al.	
	110	Barbadoro P, et al.	
	120	Leinonen MK, et al. (<45 years old)	
	120	Leinonen MK, et al. (≥45 years old)	
	149	Deguara M, et al.	

Associated factor	Study ID	Study	Pooled adherence rate
			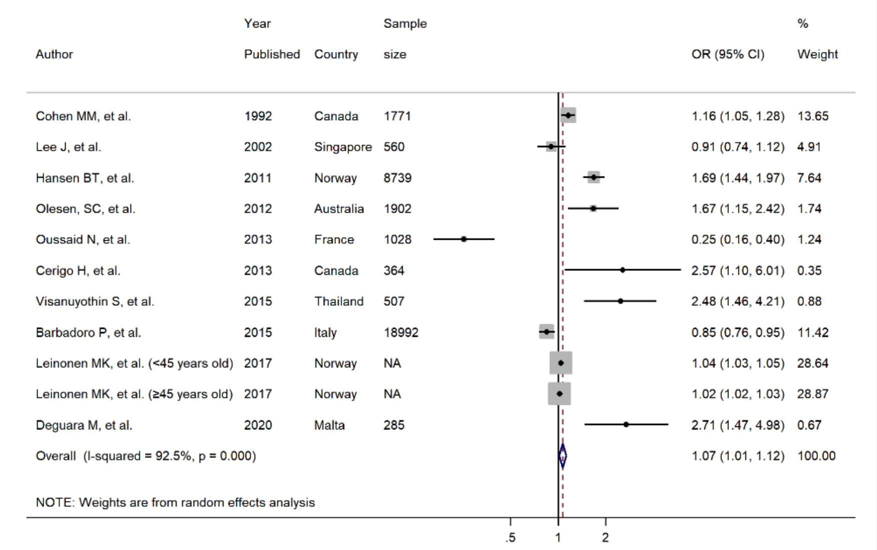
**Associated factor 10: Mental illness (n=3)**			
	35	Chen LS, et al.	
	47	Martin-Lopez R, et al.	
	138	Kaso M, et al.	

Associated factor	Study ID	Study	Pooled adherence rate
			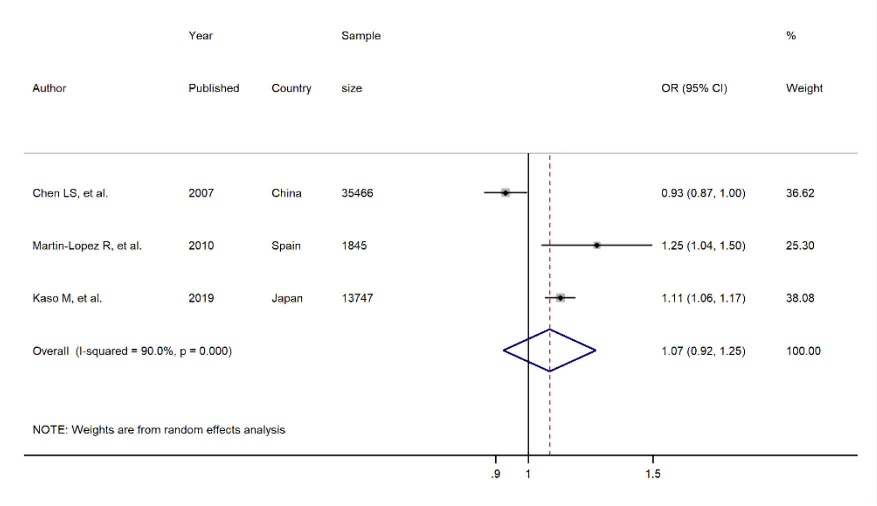
**Associated factor 11: Chronic disease (n=9)**			
	47	Martin-Lopez R, et al.	
	50	Lopez-de-Andres A, et al.	
	71	Martin-Lopez R, et al.	
	104	Ricardo-Rodrigues I, et al.	
	107	Richard A, et al.	
	117	Bianco A, et al.	
	127	Cofie LE, et al.	
	143	Zamorano-Leon JJ, et al.	
	140	Charkhchi P, et al.	

Associated factor	Study ID	Study	Pooled adherence rate

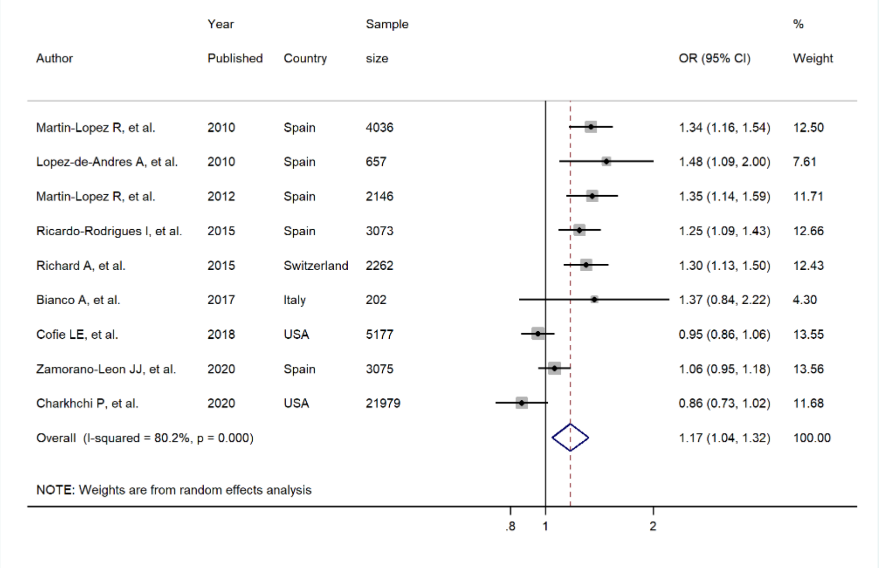

Abbreviations: BMI, body-mass index; CI, confidence interval. The definitions of some associated factors varied slightly across studies. Odds ratios for binary variable associated factors indicated better adherence to cervical cancer screening compared with those without the associated factor, except for married (vs not married), married (vs single), partnered (vs not partnered), currently married (vs never married), previously married (vs never married), married (vs divorced/widowed/separated), divorced/widowed/separated (vs single), high school (vs less than high school), higher than high school (vs less than high school), underweight/BMI <18.5 kg/m² (vs normal/BMI 18.5-24.9 kg/m²), overweight/BMI 25.0-30.0 kg/m²(vs normal/BMI 18.5-24.9 kg/m²), obesity/BMI≥30.0 kg/m² (vs normal/BMI 18.5-24.9 kg/m²), and smoker (vs non-smoker), current smoker (vs former/never), current smoker (vs never), former smoker (vs never).

Table S9 Pooled or reported adherence rate of cervical cancer screening in 28 countries with available data

Country	No. of articles	No. of data points	Sample size	Adherent women	Adherence rate (95%CI), %
Australia	5	5	48,813	25,484	57.91% (41.89-73.13)
Belgium	2	5	10,961,017	6,481,271	60.03% (57.24-62.79)
Brazil	3	3	1,625	1,204	74.53% (66.63-81.69)
Canada	8	8	158,891	121,596	75.16% (66.52-82.89)
Chile	1	1	1,042	800	76.78% (74.21-79.34)
China	8	8	5,838,110	3,041,043	34.69% (23.40-46.93)
Denmark	1	1	1,052,447	868,957	82.57% (82.49-82.64)
Estonia	1	1	3,226	1,205	37.35% (35.68-39.02)
France	2	2	7,368	5,652	82.30% (65.75-94.21)
Greece	3	3	763	378	43.27% (25.62-61.84)
Hungary	1	1	3,306	2,446	74.00% (72.50-75.50)
Israel	1	1	489,663	225,795	46.11% (45.97-46.25)
Italy	8	8	136,911	67,088	53.57% (37.53-69.25)
Japan	2	2	164,471	71,368	42.33% (37.23-47.51)
Lithuania	1	5	4,248	2,877	67.76% (62.32-72.97)
Malta	1	1	407	281	69.00% (64.50-73.50)
New Zealand	1	1	234	131	55.98% (49.61-62.35)
Norway	2	2	1,321,737	873,486	77.09% (54.22-93.63)
Singapore	1	1	726	302	41.60% (38.01-45.19)
South Africa	1	1	2,529	296	11.70% (10.45-12.95)
South Korea	8	12	31,529	15,029	46.25% (38.96-53.62)
Spain	10	10	64,338	43,612	66.04% (59.64-72.16)
Sweden	2	2	2,623,312	1,568,359	55.27% (46.15-64.22)
Switzerland	1	1	7,319	5,336	72.90% (71.88-73.92)
Thailand	1	1	595	389	65.40% (61.57-69.23)
Turkey	1	1	227	89	39.21% (32.84-45.57)
United Kingdom	2	2	1,035	815	74.79% (61.11-86.35)
United States of America	75	83	1,255,717	880,534	72.69% (68.93-76.29)

Abbreviations: CI, confidence interval.

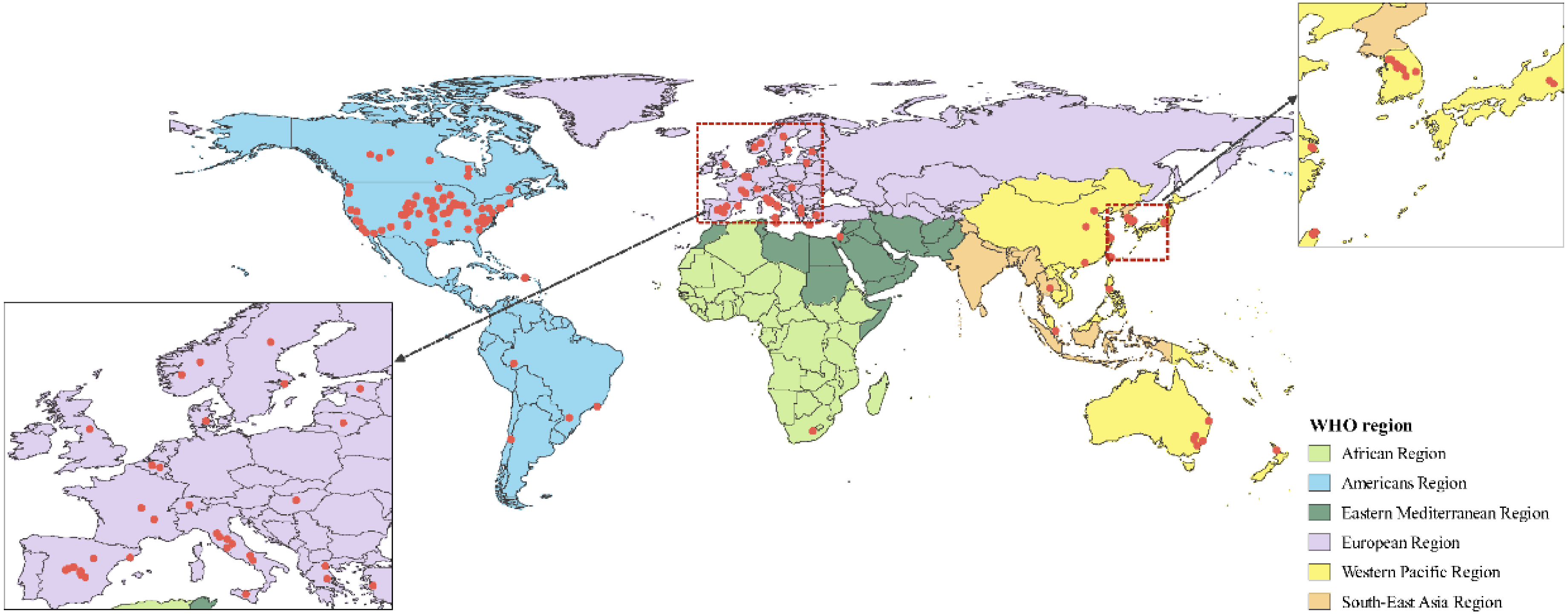

Figure S1 Geographic distribution of the included studies

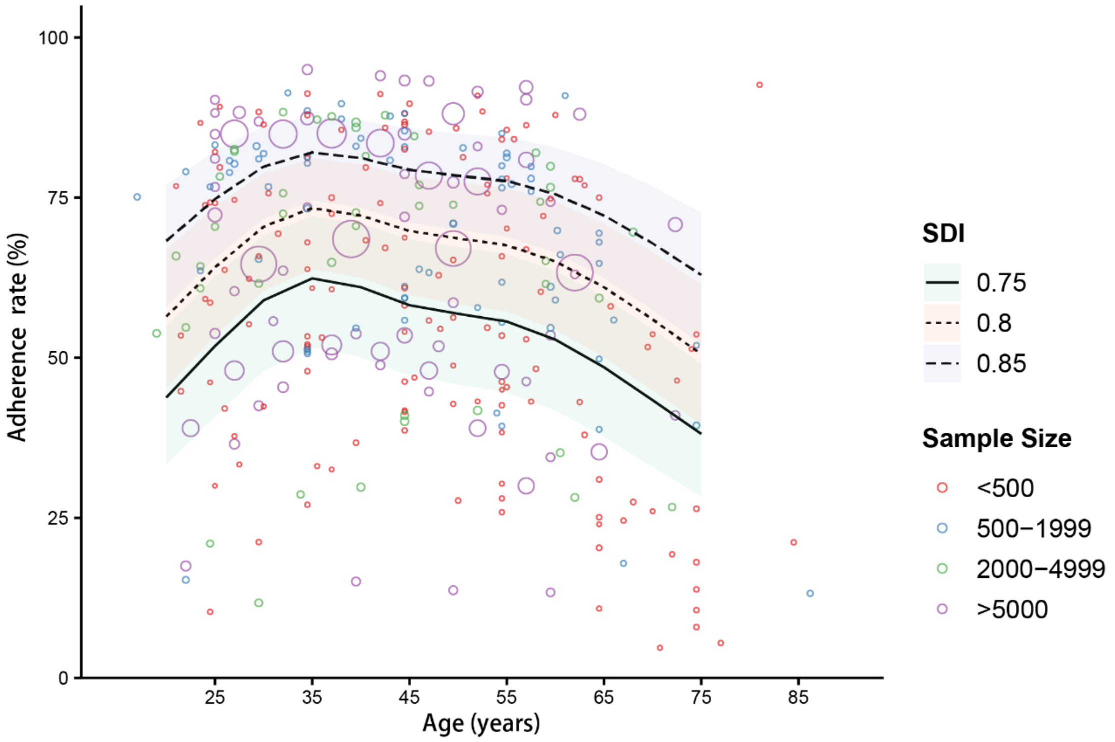

Figure S2 The estimated relation between age and adherence rates of cervical cancer screening based on informative data points from the included articles, by SDI

Abbreviations: SDI, Socio-demographic Index. The size of the bubble is proportional to the number of individuals in the sample.

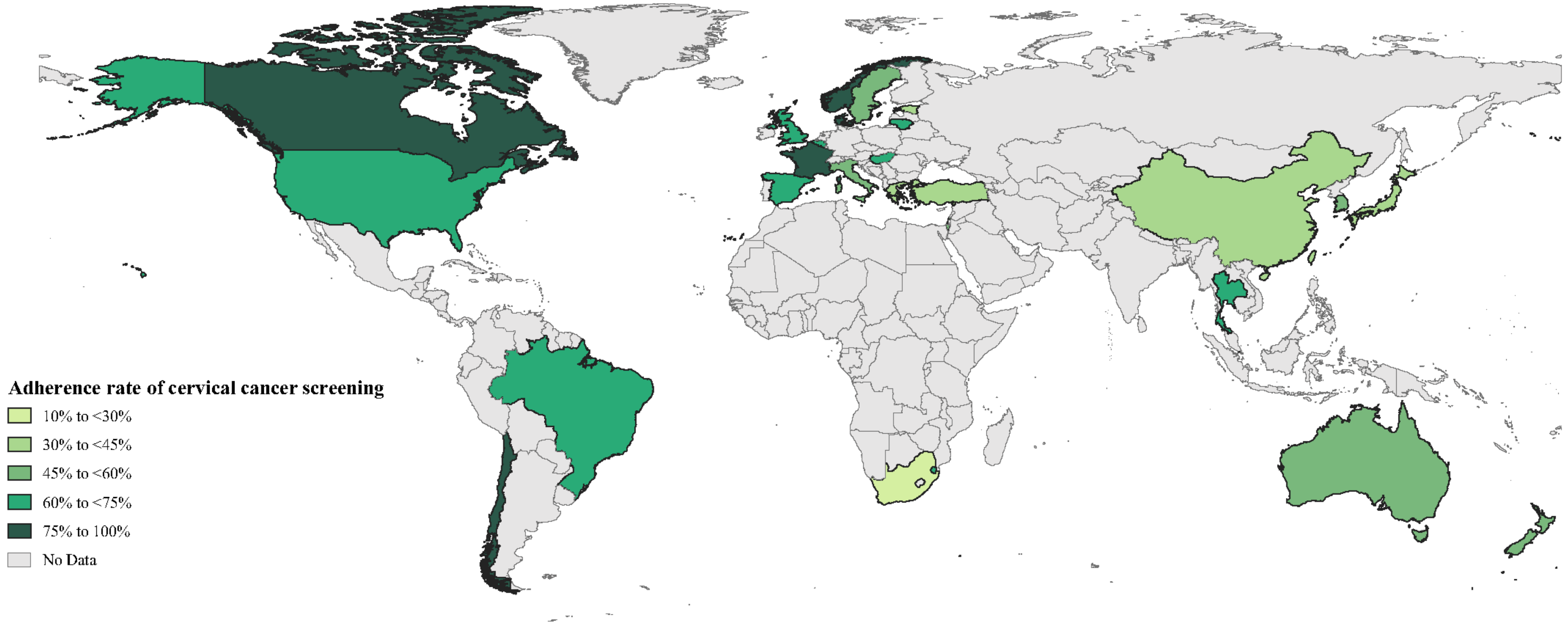

Figure S3 Adherence rate of cervical cancer screening by country
